# Enantioselective extraction of unprotected amino acids coupled with racemization

**DOI:** 10.1038/s41467-020-20402-x

**Published:** 2021-01-05

**Authors:** Haofei Huang, Yingji Jin, Mukesh E. Shirbhate, Dayoung Kang, Misun Choi, Qian Chen, Youngmee Kim, Sung-Jin Kim, Il-Suk Byun, Ming Wang, Jean Bouffard, Seong Kyu Kim, Kwan Mook Kim

**Affiliations:** 1 College of Chemistry and Chemical Engineering, Shandong University of Technology, Zibo, 255049 People’s Republic of China; 2 Department of Chemistry and Nanoscience, Ewha Womans University, Seoul, 03760 Korea; 3 Aminologics Co., R&D Center, Cheongju-si, Chungcheongbuk-do 28158 Korea; 4 Department of Chemistry, Sungkyunkwan University, Suwon, 16419 Korea

**Keywords:** Analytical chemistry, Catalysis, Synthetic chemistry methodology

## Abstract

Scalable and economical methods for the production of optically pure amino acids, both natural and unnatural, are essential for their use as synthetic building blocks. Currently, enzymatic dynamic kinetic resolution (DKR) underpins some of the most effective processes. Here we report the development of enantioselective extraction coupled with racemization (EECR) for the chirality conversion of underivatized amino acids. In this process, the catalytic racemization of amino acids in a basic aqueous solution is coupled with the selective extraction of one enantiomer into an organic layer. Back-extraction from the organic layer to an acidic aqueous solution then completes the deracemization of the amino acid. The automation of the EECR process in a recycling flow reactor is also demonstrated. Continuous EECR is made possible by the sterically hindered chiral ketone extractant **5**, which prevents the coextraction of the copper racemization catalyst because of its nonplanar geometry. Furthermore, the extractant **5** unexpectedly forms imines with amino acids faster and with greater enantioselectivity than less bulky derivatives, even though **5** cannot participate in intramolecular resonance-assisted hydrogen bonding. These features may allow EECR to challenge the preponderance of enzymatic DKR in the production of enantiomerically enriched amino acids.

## Abstract (web summary)

Dynamic kinetic resolution is a common approach for the preparation of optically pure amino acids using enzymes. Here the authors report an alternative method based on enantioselective extraction coupled with racemization, in which a bulky extractant affords hydrophobic extractable imines with amino acids rapidly, reversibly and enantioselectively.

## Introduction

Optically pure natural and unnatural amino acids (AAs) find applications as synthetic key precursors for pharmaceuticals, biomaterials, biosensors, and drug delivery systems^1–4^. Dynamic kinetic resolution (DKR), which couples an enantioselective irreversible reaction with a racemization step, has been touted as an ideal approach for the preparation of optically pure AAs using enzymatic catalysts^5–9^. However, it requires considerable optimization on a substrate-per-substrate basis to minimize interferences between racemization and resolution conditions, in conflict with time-to-market pressure, and, in many cases, degradation of the enzymes ultimately limits applicable scales. Numerous asymmetric methods have also been put forward as alternate ways to obtain enantiomerically enriched AAs^10–19^. Nevertheless, processes that are economically competitive on a production scale remain forthcoming.

Enantioselective liquid–liquid extraction (ELLE) operating at the interface of organic and aqueous layers has garnered attention because of the comparative ease of scaling up and the recyclability of the organic layers^20–24^. A serious drawback of ELLE is that the unwanted enantiomer is accumulated in one layer, limiting the yield to 50%. The yield may ideally approach to 100% if ELLE can be coupled with the racemization of the unextracted enantiomer. Such an ideal enantioselective extraction coupled with racemization (EECR) has neither been proposed nor realized to date. Another commonly encountered drawback of conventional ELLEs is the low enantioselectivity of extractants that bind substrates through noncovalent interactions. Our group has developed chiral carbonyls as extractants for the ELLE of underivatized AAs that rely on the reversible formation of covalent imine bonds, and afforded higher enantioselectivities than extractants based on noncovalent interactions^25–27^.

**Fig. 1:**
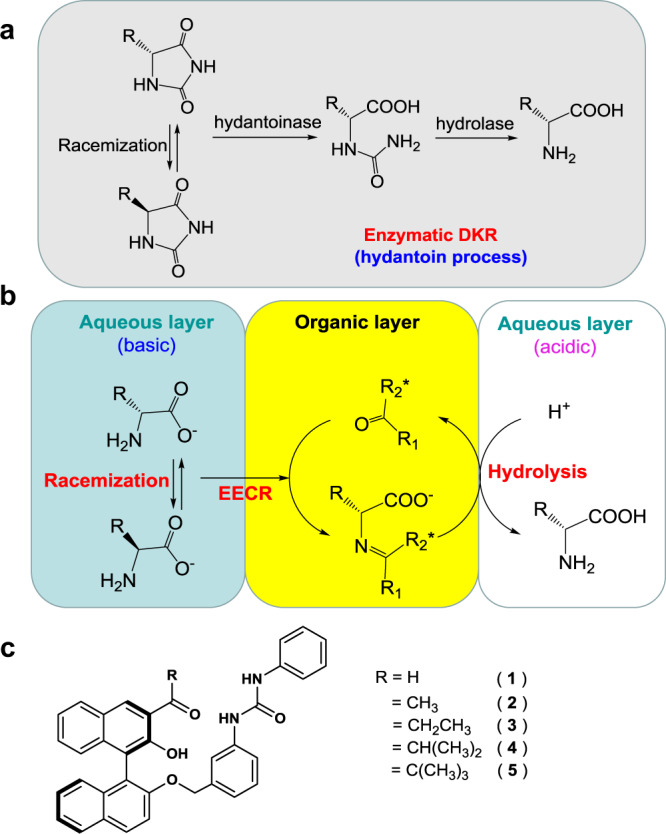
Comparison of enzymatic DKR and EECR. **a** Hydantoin process, an enzymatic DKR. **b** Enantioselective extraction coupled with racemization (EECR). **c** Carbonyl compounds studied for EECR in this work.

On the basis of these precedents, we have sought to develop the EECR of unprotected AAs with chiral carbonyl extractants. Figure 1a, b compares the hydantoin process^9^, a representative enzymatic DKR, and the EECR presented in this work. Among other advantages, the EECR uses non-derivatized AAs as feedstock, the final products are easily separated from the reactants, and the organic layer can be used repeatedly. Figure 1c shows the chiral carbonyl compounds tested as extractants for EECR in this work, which share common design elements: (i) an aldehyde or ketone group that reversibly forms imines with the amino group of AAs; (ii) an –OH moiety in *ortho* to the carbonyl group susceptible to stabilize imine formation through an intermolecular resonance-assisted hydrogen bond (RAHB), as proposed by Gilli et al.^28^; (iii) a tethered urea that increases the affinity and selectivity of the extractant for its AA substrate through secondary hydrogen-bonding interactions with the carboxylate group.

Accomplishing the EECR of AAs requires the tackling of independent problems. First, the racemization should be rapid at ambient conditions, and the racemization catalyst should be sequestered in the aqueous layer to avoid the racemization of the extracted AA. Second, the enantioselectivity of the extractant for the desired enantiomer needs to be as high as that of rival enzymatic catalysts. Carbonyl extractants previously developed in our group have shown high enantioselectivity in the ELLE of general AAs. However, additional upgrading processes were nevertheless required to meet the needs of customers, substantially increasing the cost of the commercial production of enantiomerically pure AAs. Finally, the rates of both imine formation and its hydrolysis to obtain the AAs must be sufficiently fast to enable a high-throughput EECR process. Previously developed carbonyl extractants capable of exclusive enantioselectivity through the formation of highly stable imine derivatives have failed this last criterion, requiring harsh conditions for hydrolysis^27^. Despite these competing requirements, here we report that a continuous EECR process could be developed, unexpectedly and paradoxically, using the highly sterically hindered ketone **5** as an extractant.

## Results

### Exclusive enantioselectivity of 5 in the ELLE of AAs

**Fig. 2:**
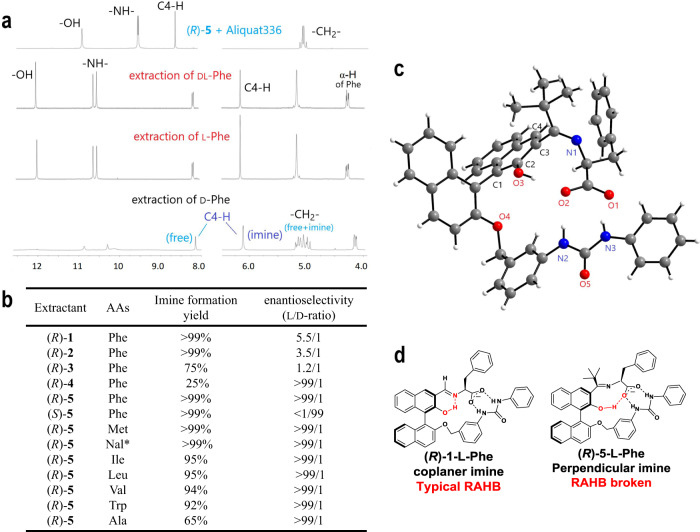
The result of ELLE and the imine crystal structure. **a** Representative ^1^H NMR spectra of the organic layer in the ELLE of Phe with (*R*)-**5**: (*R*)-**5** in the presence of Aliquat 336 (top); after ELLE of dl-Phe and separation of the organic layer (2nd row); after ELLE of l-Phe and separation of the organic layer (3rd row); after ELLE of d-Phe and separation of the organic layer (bottom). **b** The imine formation yields and enantioselectivities in the organic layers determined by ^1^H NMR studies after the ELLE of AAs with **1**–**5**. General ELLE conditions: organic layer, 0.10 M extractant and 1.05 equiv. Aliquat 336 in 1 mL CDCl_3_; aqueous layer, 1.0 M dl-AA in 1 mL H_2_O with pH 12.0 at 25 °C; 12 h stirring. *Nal stands for naphthylalanine, an unnatural amino acid. **c** The crystal structure of (*R*)-**5**-l-Phe. **d** Comparison of (*R*)-**1**-l-Phe and (*R*)-**5**-l-Phe.

Figure 2a shows the result of an ELLE experiment that was carried out with phenylalanine (Phe) by the reported method^26^ using (*R*)-**5** as an extractant. ^1^H NMR analysis of the organic layer after the extraction of L-Phe for 12 h at room temperature shows a signal centered at 4.26 parts per million (p.p.m.) that corresponds to the α-proton of Phe in the resulting imine, (*R*)-**5**-L-Phe, confirming that its formation is involved in the extraction. By contrast, the signal of the α-proton of the diastereomeric imine (*R*)-**5**-d-Phe appears at 4.13 p.p.m., allowing for the two diastereomers to be distinguished by proton nuclear magnetic resonance (^1^H NMR). Alternately, the signals of the C4-H of the imine appearing at 6.1–6.2 p.p.m. can also be a marker for the identification of the two diastereomers. Upon the attempted extraction of d-Phe by (*R*)-**5** under the same conditions, the complex benzyl –CH_2_– signals centered at 4.9–5.2 p.p.m. indicate the presence of both (*R*)-**5**-d-Phe and unreacted (*R*)-**5** in an approximate 65:35 ratio. Thus, it can be concluded that the yield of imine formation is much lower for the mismatched d-Phe/(*R*)-**5** pair. Extending the extraction to 24 h with d-Phe did not further depress the proportion of the free extractant (*R*)-**5**. By contrast, imine formation was complete within 4 h for the matched l-Phe/(*R*)-**5** pair.

Finally, Fig. 2a gives the surprising result that the ^1^H NMR spectrum obtained for the organic layer after the extraction of dl-Phe is identical to that for l-Phe, demonstrating the exclusive selectivity of (*R*)-**5** for the latter. The ELLE experiments for other AAs such as isoleucine (Ile), leucine (Leu), methionine (Met), valine (Val), alanine (Ala), tryptophan (Trp), and naphthylalanine (Nal) gave similar results. Especially in the cases of Val and Ala, the d-forms were not extracted at all (see the ^1^H NMR and high-performance liquid chromatography (HPLC) data in Supplementary information, Section 5.1).

Figure 2b compares the results of ELLE experiments with extractants **1**–**5** for Phe, and with extractant **5** for other representative AAs. The enantioselectivities for **1** (aldehyde), **2** (methyl ketone), and **3** (ethyl ketone) decreased with increased bulk, before increasing dramatically for **4** (isopropyl ketone) and **5** (*tert-*butyl ketone). Moreover, although the yield of imine formation decreased with steric demand in the order of **1** > **2** > **3** > **4**, it increased unexpectedly and abruptly for **5**.

(*R*)-**5** is highly selective for the AAs, particularly bearing hydrophobic side chains, including the unnatural AA naphthylalanine, an important pharmaceutical precursor^29^. As expected, the enantiomer (*S*)-**5** is selective in ELLE experiments for the extraction of d-AAs. Although most ELLE experiments were run in chloroform to simplify NMR analyses, comparable results were obtained using dichloromethane, 1,2-dichloroethane, and non-halogenated solvents, such as toluene, *tert*-butyl methyl ether, and 2-methyltetrahydrofuran (see data in Supplementary information, Sections 6.3.10–6.3.12).

The signal of the –OH group of (*R*)-**5**-l-Phe, as shown in Fig. 2a, appears at 12.1 p.p.m, which is shifted upfield compared to those of (*R*)-**1**-l-Phe, (*R*)-**2**-l-Phe, and (*R*)-**3**-l-Phe that appear between 16 and 18 p.p.m. (see Supplementary Fig. 10). This indicates that the imine of **5** has a different structure than the imines of **1**, **2**, and **3**. Indeed, the crystal structure of (*R*)-**5**-l-Phe displayed in Fig. 2c demonstrates that RAHB is broken; the –OH group of (*R*)-**5** is not hydrogen bonded to the imine nitrogen, but instead to the carboxylate group of l-Phe. The imine group is also twisted out of plane with respect to the naphthol ring, contrasting with the previously reported coplanar RAHB imine (*R*)-**1**-l-Phe as shown in Fig. 2d.

**Fig. 3:**
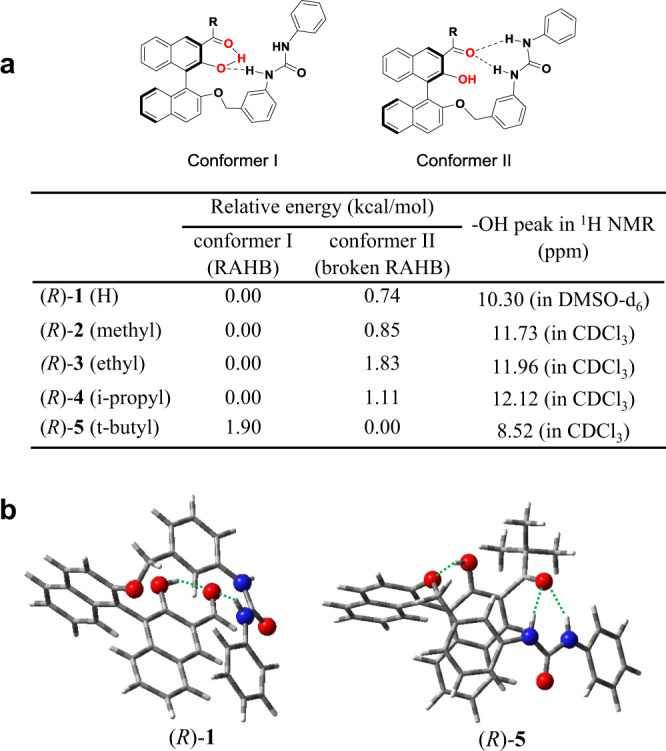
Calculated structures of (*R*)-1 and -5. **a** Conformers with RAHB (I) or broken RAHB (II), their calculated relative energies, and experimental ^1^H NMR chemical shift of the –OH resonances. **b** Energy-minimized structures showing a RAHB for (*R*)-**1** and a broken RAHB with perpendicular carbonyl for (*R*)-**5**.

Figure 3a compares the energies of two possible conformers of **1–5**, proposed by density functional theory (DFT) calculations with the BLYP functional and the 6-311G* basis set supplemented with D3 dispersion^30–33^. In conformer I, the carbonyl oxygen is hydrogen bonded to the hydroxy group, which is known as RAHB. In conformer II, the carbonyl group is involved in hydrogen bonding with the two uryl NH protons, that is, RAHB is broken, and the carbonyl plane is perpendicular to the binaphthol ring.

For compound **1**, conformer I is more stable than conformer II by 0.74 kcal/mol, which is consistent with the generally accepted idea that RAHB stabilizes this preferred conformation. By contrast, for **5**, the same calculation suggests that conformer I with RAHB interaction is less stable than conformer II by 1.90 kcal/mol, illustrating that bulkier carbonyl substituents raise the energy of the coplanar conformer. Figure 3b compares energy-minimized structures of **1** and **5**. A remarkable finding is that conformer II is favored only for **5**. The ^1^H NMR chemical shift of the phenolic hydrogens provides experimental validation for these DFT results: only for **5** is the OH signal consistent with a broken RAHB.

Energy calculations based on the crystal structure predicts that (*R*)-**5**-l-Phe is more stable than (*R*)-**5**-d-Phe by 5.77 kcal/mol, which rationalizes the exceptionally high stereoselectivity obtained in ELLE experiments (see details in Section 8.3 of the Supplementary information).

Pyridoxal-5′-phosphate (PLP) derivatives described by Breslow as artificial enzymes^34^ have been widely studied as receptors for amines^35,36^. The RAHB is a key feature of PLP-derived receptors, where it is generally considered to contribute to the stability of the resulting imines and to provide greater stereoselectivity in the recognition of chiral amines. It is thus paradoxical that the bulkiest carbonyl extractant **5** was found to rapidly form imines with exquisite enantioselectivity using AAs, even in the absence of RAHB. The experimental results for **5** demonstrate that RAHB assistance is not an indispensable element, and that even bulky carbonyl compounds do not have to be excluded in the design of receptors for AAs.

### Racemization of AAs, Cu(II) sequestration, and successful EECR

It has been reported that AAs were racemized by the catalytic mixture of Cu^2+^ (10 mol%)/pyridoxal (1 equiv.) or Cu^2+^ (10 mol%)/PLP (10 mol%) at a pH of ~9^37,38^. In an effort to find better conditions compatible with EECR, we, fortunately, observed that just 1 mol% of PLP and 1.0 mol% of Cu^2+^ were sufficient to completely racemize AAs, such as Ala, Phe, Leu, Trp, and Met within a few hours at room temperatures and at a pH of ~12. Under these conditions, the racemization of Phe occurred with a half-life of ca. 30 min, and neither catalyst deactivation nor the formation of undesired side products derived from the AAs was observed over extended periods (Supplementary Figs. 21, 22, and 38(c)). Higher temperatures are required to complete the racemization of bulkier AAs, such as Ile (40 °C) and Val (60 °C) on a comparable timescale (see Supplementary Table 2 in Supplementary information, Section 6.1).

In a typical EECR experiment, a solution of the chiral extractant **5** in an organic solvent immiscible with water was stirred with the above aqueous AA solution containing the racemization catalyst. Under these conditions, the EECR experiments could be performed with concentrations of AAs in the aqueous layer as high as 1–2 M. For Phe, proton NMR monitoring of the organic layers show that the formation of the imine is rapid (ca. 60% reached within 30 min), but that extending the mixing for an additional 2–6 h is beneficial to reach a steady state with an exclusive thermodynamic selectivity for the desired diastereomeric imine (see Supplementary Fig. 26).

Following phase separation, the organic layers were back-extracted with aqueous HCl to hydrolyze the imine, providing the liberated **5** and the enantioenriched (≥98:2 enantiomeric ratio (e.r.) by HPLC) AA as its hydrochloride salt. Hydrolysis is rapid, reaching completion within 1 h at room temperature in the presence of 2 equiv. of 2 N HCl (see Supplementary Fig. 20). NMR analysis of the organic layer following the hydrolysis shows the pristine **5**, which could be recycled over a minimum of 20 EECR cycles without showing any sign of degradation (see Supplementary Fig. 19). Neutralization of the aqueous layer allowed for the isolation of the AAs by precipitation. Isolated AA yields over four EECR cycles starting from the racemic AA could reach 76% (up to 305% with respect to **5**), demonstrating the superiority of the EECR over the theoretical limit of 50% imposed by the ELLE approach (Table 1 and Supplementary Figs. 27–33). More moderate isolated yields were obtained with hydrophilic AAs due to their greater solubility; however, the performance of the EECR process itself was not diminished. Comparable yields and selectivities were obtained using either halogenated solvents, toluene, 2-methyltetrahydrofuran, or *tert*-butyl methyl ether. Finally, it should be noted that the EECR was equally as effective in the deracemization of AAs (e.g., dl-to-l) as in the conversion of one enantiomer into another (e.g., d-to-l; see Supplementary information, Section 6.3).

**Table 1:** Isolated yields and enantiopurity obtained over four EECR cycles with (*R*)-**5** (see details in Supplementary information, Sections 6.3.3–6.3.12).

AAs^a^	Isolated yield (%)^b^	Isolated AA enantiopurity^c^
Phe	73.2 (293)	>98
Phe^d^	75.3 (301)	>98
Phe^e^	76.3 (305)	>98
Phe^f^	73.2 (293)	>98
Met	70.9 (278)	>98
Nal^g^	73.4 (293)	>98^h^
Ile	72.1 (288)	>98
Leu	75.8 (303)	>98
Val	75.1 (300)	>98
Trp	74.5 (298)	>98

^a^General EECR conditions: organic layer: (*R*)-5 (0.90 g, 1.5 mmol) and 1.2 equiv. of Aliquat 336 in CDCl_3_ (10 mL); aqueous layer: AAs (6.0 mmol), NaOH (0.26 g, 6.6 mmol), PLP (0.016 g, 0.060 mmol), and CuSO_4_ (0.010 g, 0.060 mmol) in H_2_O (5 ml) at pH 12.0 and 25 °C; 6 h stirring per cycle.

^b^Isolated yields of the AAs after four extraction/hydrolysis cycles based on the racemic AA, or based on (*R*)-**5** in parentheses.

^c^Enantiomeric purity (%) of the isolated AAs after four extraction cycles (HPLC).

^d^EECR conducted in PhMe.

^e^EECR conducted in *t*-BuOMe.

^f^EECR conducted in 2-MeTHF.

^g^Naphthylalanine, an unnatural amino acid.

^h^Assessed by ^1^H NMR integration.

**Fig. 4:**
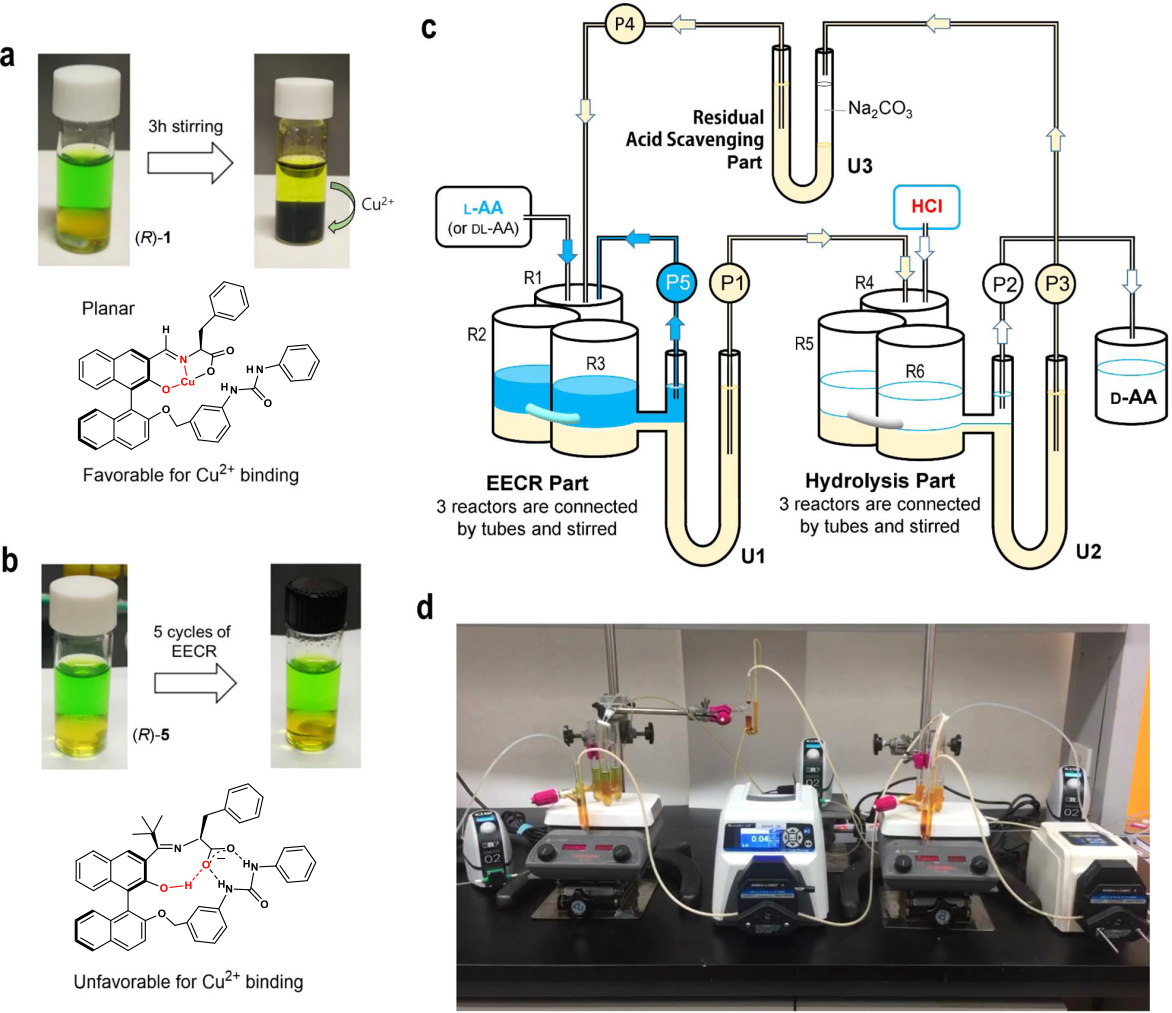
Cu^2+^ sequestration and EECR in continuous flow. **a** When (*R*)-**1** was used as the extractant, a striking color change indicating the transfer of Cu(II) from the aqueous to the organic layer was observed. **b** When (*R*)-**5** was used as the extractant, the color did not change over five EECR cycles. **c** Schematic design of continuous reaction system (CRS) that carries out EECR and hydrolysis reactions repeatedly. **d** Photographs of CRS in operation on the lab bench. The apparatus was manufactured to accommodate a total solution volume of 100 mL.

The transfer of Cu^2+^ ions from the aqueous layer to the organic layer was a serious problem that previously impeded the successful implementation of EECR. The use of **5** as an extractant also solved this problem. Figure 4a, b compares the color changes of the solutions of **1** and **5** during EECR trials for Phe. In the case of **1**, the transfer of Cu^2+^ ion to the organic layer resulted in the darkening of the biphasic mixture within 3 h of stirring. The same features were observed during EECR attempts using **2** and **3**. Thus, EECR could not be run using **1**, **2**, or **3** as extractants. The coplanarity of the imine and adjacent phenol group probably favors the binding of Cu^2+^ ions.

When **5** was used as an extractant, however, both the organic and aqueous layers conserved their initial colors, even after several repeated cycles of imine formation and hydrolysis, indicating the sequestration of Cu^2+^ ions in the aqueous layer. Further analysis by atomic absorption spectrometry confirmed, remarkably, that leaching of Cu(II) ions in the organic layer did not occur ([Cu] ≤ 1 p.p.m.), even after 40 h stirring under EECR conditions with **5**. The orthogonality of the imine plane to that of the naphthol ring in **5** likely prevents the formation of tight salicylaldimine-type Cu^2+^ complexes.

### EECR in a continuous flow reactor

Continuous EECR in flow was developed following the encouraging results of the above EECR experiments. The designed apparatus, shown in Fig. 4c, is composed of three stages that, respectively, perform the EECR, hydrolysis, and residual acid scavenging. EECR is performed in consecutive stirred reactor chambers R1–R3, which flow into the U-tube U1 to allow for the separation of the reaction mixture into organic and aqueous layers. A peristaltic pump P1 transfers the separated organic layer from U1 to the consecutive stirred reactor chambers R4–R6 that carry the hydrolysis of the imine. The following separation of the layers in U-tube U2 provides the organic layer containing **5** and the aqueous layer containing enantiomerically pure AA as its HCl salt. The acidic organic layer flowing from U2 is transferred to U-tube U3 for residual acid scavenging before it is recycled into R1, where a new EECR cycle start. During the process, the starting AA is also fed into the reactor R1 in discrete additions to compensate for its depletion. This system is an expanded variant of the well-known U-tube design to perform a continuous transfer of material from one side to the other^39–41^.

Using the apparatus shown in Fig. 4d, EECR in flow enabled the continuous conversion of common l-Phe to the more valuable d-Phe. The apparatus was charged with a solution of the chiral extractant (*S*)-**5** (4.5 g, 7.6 mmol) in dichloromethane (40 mL), and l-Phe (5.0 g, 30 mmol) alongside the racemization catalyst in the aqueous layer (20 mL). The reactor was operated continuously for 40 h. During this period, two additional equivalents of l-Phe (2.5 g, 15.2 mmol) were fed in. From the hydrolyzed aqueous layer was isolated 4.9 g (29.7 mmol) of the solid d-Phe with enantiopurity of ≥98:2 e.r., corresponding to 3.9 equiv. of the extractant, or 65% based on the total l-Phe used. No sign of degradation of the chiral extractant **5** was detected after 40 h of continuous operation, demonstrating the appeal of the EECR of AAs relying on **5** for further process intensification and commercial-scale production.

In conclusion, we have demonstrated the EECR as a DKR process for the deracemization of unprotected AAs using only extractive processes. The success of EECR relied on the highly sterically hindered pivaloylated extractant **5**, and the identification of a compatible catalyst system for the racemization of AAs under ambient conditions. The orthogonal arrangement of the pivaloyl group and the naphthol ring in **5** prevents the co-extraction of Cu^2+^ ions. The extractant **5** shattered the widely accepted view that the RAHB found in vitamin B_6_ cofactor mimics is required for the formation of stable imines, which may open new perspectives in imine chemistry and its applications. A great advantage of EECR compared to other processes like enzymatic DKR and asymmetric chemical syntheses are the potential for the development of automated continuous processes as shown in the flow reactor designed and operated in this work. Although the 100-mL continuous reactor was so far only operated up to multigram reaction scales, this work foreshadows practical and cost-effective extensions far beyond this scale for the deracemization of underivatized AAs.

## Methods

### Materials

NaH (60%), *tert*-butyllithium (1.6 M in hexane), and other conventional reagents were obtained from Sigma-Aldrich, Alfa-Aesar, or TCI, and used as received without further purification. Enantiomerically pure 2,2ʹ-binol-3-carboxylic acid was provided by Aminologics (Seoul, Korea). Compound (*R*)-**1** and (*R*)-**2** were prepared following literature procedures^25,26^. The syntheses and characterization data for (*R*)-**3**–(*R*)-**5** are described in Supplementary information, Section 1.

### Instruments and analyses

^1^H NMR spectra were recorded on a Bruker AVANCE 300 spectrometer (300 MHz) at the National Research Facilities and Equipment Center (NanoBio·Energy Materials Center) at Ewha Woman’s University. Chemical shifts were quoted in p.p.m. referenced to 0.0 p.p.m. for tetramethylsilane. Carbon-13 NMR spectra were recorded on Bruker AVANCE 300 spectrometer (75 MHz), and were fully decoupled by broadband proton decoupling. Chemical shifts were reported in p.p.m. referenced to the centerline of a triplet at 77.0 p.p.m. of chloroform-*d*. High-resolution mass spectra were recorded on an Agilent Mass spectrometer on EI or FAB mode. Agilent 1260 HPLC system using Sumichiral OA-5000 or OA-6100 column was used for determination of the enantiopurities of AA solutions.

### Representative ELLE experiment for an AA with (*R*)-5 as the extractant

The organic layer was prepared by dissolution of (*R*)-**5** (0.60 g, 1 mmol) and Aliquat 336 (0.43 g, 1.05 mmol) into CHCl_3_ (2 mL). The aqueous layer was prepared by the addition of dl-AAs (4.0 mmol) to a solution of NaOH (0.16 g, 4.0 mmol) in water (2 mL). The two solutions were mixed and stirred vigorously at room temperature (20–25 °C). The stirring was continued until the imine formation was completed.

### Representative EECR experiment for an AA with (*R*)-5 as the extractant

The organic layer was prepared by dissolution of (*R*)-**5** (0.60 g, 1.0 mmol) and Aliquat 336 (0.43 g, 1.05 mmol) in CDCl_3_ (1.0 mL). The aqueous layer was prepared by the addition of d-AA (4.0 mmol), NaOH (0.16 g, 4.4 mmol) in water (2.0 mL), and both PLP (0.011 g, 0.04 mmol) and CuSO_4_ (0.0064 g, 0.04 mmol) were added to the aqueous solution. The two solutions were mixed and stirred vigorously in a sealed glass vial at room temperature (20–25 °C) for 3–6 h, until the imine formation was completed.

### Hydrolysis of the imine in the organic layer (back-extraction)

After the extraction in the experiment of ELLE or EECR, the organic layer was separated and stirred with 2.0 mL of 2.0 N HCl solution for 2 h at room temperature, which led to the clean recovery of the extractant (*R*)-**5** and Aliquat 336 in the organic layer and the enantiomerically pure AA in the aqueous layer. This process was monitored by ^1^H NMR and HPLC analysis.

### Continuous EECR using the specially designed apparatus shown in Fig. 4d

The aqueous layer in the EECR part was prepared from l-Phe (6.0 g, 38 mmol), PLP (0.03 g, 0.12 mmol), and CuSO_4_(0.0096 g, 0.06 mmol) dissolved in 20 mL water, and the pH was adjusted to 12.0 by the addition of NaOH. The organic layer was prepared by dissolving (*S*)-**5** (4.5 g, 7.6 mmol) and Aliquat 336 (4.3 g, 8.5 mmol) in 40 mL of CH_2_Cl_2_. The hydrolysis chambers R4–R6 used a 2.0 N aqueous HCl layer. The residual acid scavenging chamber U3 used a 1.0 M aqueous Na_2_CO_3_ layer to neutralize residual HCl in the organic layer. Transfer rates of the pumps were controlled to be ~0.15 mL/min.

## Supplementary information

Supplementary Information

## Data Availability

All experimental data are available in the main text or the Supplementary information. The X-ray crystallographic coordinates for structures reported in this study have been deposited at the Cambridge Crystallographic Data Centre (CCDC), under deposition number 1948381 [10.5517/ccdc.csd.cc23dg00]. These data can be obtained free of charge from The Cambridge Crystallographic Data Centre via www.ccdc.cam.ac.uk/data_request/cif. Data are also available from the corresponding author on request.
