# Supplementary material for: Enantioselective extraction of unprotected amino acids coupled with racemization
Source: Nat Commun. 2021 Jan 5;12:125. doi: 10.1038/s41467-020-20402-x (PMC7785727; doi:10.1038/s41467-020-20402-x)
Supplement: Supplementary file 1 — Supplementary Information [file 41467_2020_20402_MOESM1_ESM.pdf]

Supplementary Information

Enantioselective Extraction of Unprotected Amino Acids Coupled with Racemization

Huang et al.

Table of Contents

1. General Informations and Synthetic Details. S2

2. Extraction of phenylalanine with (*R*)-5..S12

3. Comparative data for the extraction of phenylalanine with (*R*)-1~5..............S14

4. Crystallographic data for [(*R*)-5-L-Phe]$^-$[TPP]$^+$ S16

5. ELLE and back-extraction with (*R*)-5 ....S17

6. EECR with (*R*)-5 for representative amino acids S288

7. Continuous EECR of L-Phe with (*S*)-5. ....S533

8. Model calculations S57

1. General Information and Synthetic Details.

1.1. Materials

NaH (60%), *tert*-butyllithium (1.6 M in hexane) and other conventional reagents were obtained from Sigma-Aldrich, Alfa Aesar or TCI, and used as received without further purification. (*S*)-2,2′-Binol-3-carboxylic acid was provided by Aminologics (Seoul, Korea). 3-Phenyluryl-benzyl-bromide, and compounds (*R*)-**1** and (*R*)-**2** were prepared following literature procedures.[1,2]

1.2. Analytical Methods

^{1}H NMR spectra were recorded on a Bruker AVANCE 300 instrument (300 MHz). Chemical shifts are reported in parts per million (ppm) referenced to 0.0 ppm for tetramethylsilane. ^{13}C NMR spectra were recorded on Bruker AVANCE 300 instrument (75 MHz) under full decoupling by broad band proton irradiation. Chemical shifts are reported in ppm referenced to the center line of the chloroform-d triplet at 77.0 ppm. High-resolution mass spectra (HRMS) were recorded on an Agilent Mass spectrometer using either EI or FAB mode. Enantiomeric ratio values were determined on an Agilent 1260 HPLC system using Sumichiral OA-5000 or OA-6100 columns.

1.3. General method for the preparation of ketobinol compounds

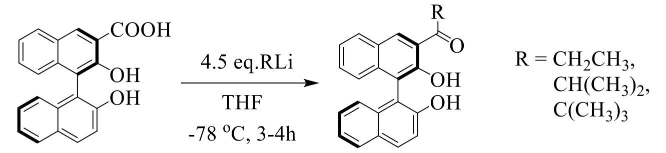

ketone binol compouds

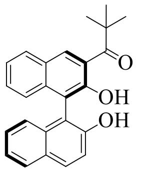

(*R*)-3-*tert*-Butyl-2,2′-dihydroxy-1,1′-binaphthyl ketone: To a THF (50 mL) solution containing (*S*)-2,2′-binol-3-carboxylic acid (3.3 g, 10 mmol) at -78 ºC was added dropwise *tert*-butyllithium (1.7 *M* in pentane, 26.5 mL, 45 mmol). The solution was stirred at this temperature for 4 h, before quenching by the addition of a saturated NH_4Cl solution. The volatiles were evaporated under reduced pressure, and then submitted to a water-ethyl acetate (3 x 25 mL) workup. The combined organic layers were dried over $MgSO_4$, filtered, and concentrated in vacuo. The crude product by purified by column chromatography on silica gel using EA/hexane (1:15) as the eluent to give (*R*)-3-*tert*-butyl-2,2′-dihydroxy-1,1′-binaphthyl ketone as a yellow solid (2.77g, 75%). $[\alpha]_D$ = +41 (c 0.54, $CHCl_3$). ^{1}H NMR (300 MHz, $CDCl_3$) δ 11.61 (s, 1H), 8.79 (s, 1H), 7.89-7.97 (m, 3H), 7.16- 7.43 (m, 5H), 7.01-7.20 (m, 2H), 5.07 (s, 1H), 1.63 (s, 9H). ^{13}C NMR (75 MHz, $CDCl_3$) δ 212.7, 155.8 151.5 136.6, 134.1, 130.3, 129.3, 123.4, 113.9, 45.4, 29.1. HRMS-EI (m/z) calcd for $C_{25}H_{22}O_3$: 371.1569; found 371.1569.

The ethyl ketone binol and *iso*-propyl ketone binol were prepared according to the general method described above, using either ethyllithium or isopropyllithium in place of *tert*-butyllithium.

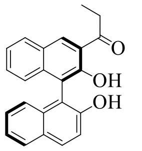

(*R*)-3-Ethyl-2,2′-dihydroxy-1,1′-binaphthyl ketone: yield 78%. $[\alpha]_D$ = +34.6 (c 1.2, $CHCl_3$). ^{1}H NMR (300 MHz, $CDCl_3$) δ 12.14 (s, 1H), 8.67 (s, 1H), 7.89~8.00 (m, 3H), 7.09- 7.19 (m, 7H), 5.03 (s, 1H), 3.36 (q, *J* = 6 Hz, 2H), 1.36 (t, *J* = 9 Hz 3H). ^{13}C NMR (75 MHz, $CDCl_3$) δ 207.4, 155.7 151.4 137.5, 133.9, 130.5,

126.6, 124.7, 117.4, 113.9, 31.9, 8.37. HRMS-EI (m/z) calcd for $C_{23}H_{18}O_3$: 342.1256; found 342.1258.

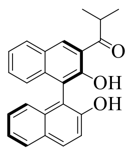

(*R*)-3-Isopropyl-2,2′-dihydroxy-1,1′-binaphthyl ketone: yield 70%. $[\alpha]_D$ = +34.3 (c 1.0, $CHCl_3$). ^1H NMR (300 MHz, $CDCl_3$) δ 12.29 (s, 1H), 8.69 (s, 1H), 7.89~8.01 (m, 3H), 7.16- 7.43 (m, 7H), 5.02 (s, 1H), 3.95-4.04 (m, 1H), 1.43 (d, *J* = 6 Hz, 6H). ^{13}C NMR (75 MHz, $CDCl_3$) δ 211.2, 156.3 151.4 137.6, 134.1, 130.3, 129.3, 124.7, 114.0, 35.4, 19.8, 19.6. HRMS-EI (m/z) calcd for $C_{24}H_{20}O_3$: 356.1412; found 356.1411.

1.4 General method for the preparation of extractant (*R*)-5 and its analogues

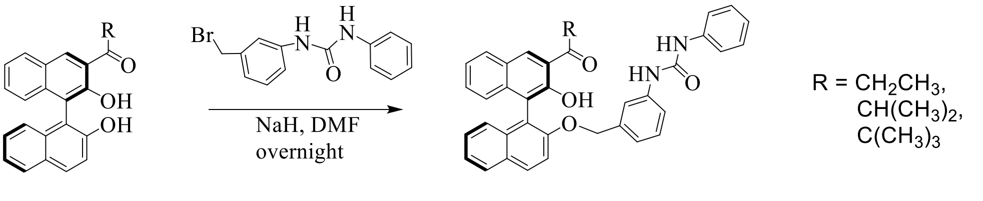

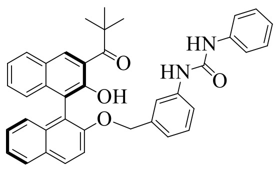

(*R*)-**5**: A solution of (*R*)-3-*tert*-butyl-2,2′-dihydroxy-1,1′-binaphthyl ketone (3.7 g, 10 mmol) in DMF (50 mL) was added dropwise to a stirred slurry of NaH (60% in mineral oil, 0.44 g, 11 mmol) in DMF (50 mL) at 0˚C. After 2 h of stirring at this temperature, 3-phenyluryl-benzyl bromide (3.36 g, 11 mmol) was added, and the stirring was continued at room temperature overnight. The reaction was then quenched with a saturated NH_4Cl solution and extracted with ethyl acetate and water. After evaporation of the volatiles from the combined organic layers, the crude product was purified by column chromatography on silica gel using EA/hexane (1:7) as the eluent to furnish compound (*R*)-**5** as a yellow solid (2.67 g, 45%). $[\alpha]_D$ = 109 (*c* 0.55, $CHCl_3$). ^1H NMR (300 MHz, $CDCl_3$) δ 11.3 (s, 1H), 8.28 (s, 1H), 7.83~7.99 (m, 5H),

7.51-7.55 (d, J = 9 Hz 1H), 7.21-7.43 (m, 10 H), 7.12-7.15 (d, J = 6 Hz 1H), 6.98-7.04 (m, 2H), 6.73 (s, 1H), 6.65-6.68 (d, J = 9 Hz, 1H）, 5.02 (d, J = 12 Hz, 1H), 4.88 (d, J = 12 Hz, 1H), 1.52 (s, 9H). ^{13}C NMR (75 MHz, $CDCl_3$) δ 215.5, 154.4, 153.6, 151.4, 138.9, 137.7, 135.7, 124.2, 119.8, 115.3, 70.3, 45.6, 28.2. HRMS-EI (*m/z*) calcd for $C_{39}H_{34}N_2O_4$: 595.2252; found 595.2253.

(*R*)-**3** and (*R*)-**4** were similarly prepared using the above procedure.

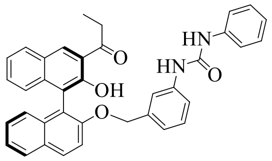

(*R*)-**3**: yield 41%. $[\alpha]_D$ = 129.6 (*c* 0.54, $CHCl_3$). ^{1}H NMR (300 MHz, $CDCl_3$) δ 11.96 (s, 1H), 8.43 (s, 1H), 7.82~7.89 (m, 3H), 6.89-7.34 (m, 14H), 6.81 (s, 1H), 6.66 (s, 1H), 6.59-6.62 (d, J = 9 Hz, 1H), 4.86 (s, 2H), 3.12-3.19 (q, J = 9 Hz, 2H), 1.22 (t, J = 9 Hz, 3H). ^{13}C NMR (75 MHz, $CDCl_3$) δ 207.9, 154.4, 153.9, 153.1, 138.2, 137.5, 132.7, 124.9, 120.0, 115.4, 70.8, 31.8, 8.25. HRMS-EI (*m/z*) calcd for $C_{37}H_{30}N_2O_4$: 566.2206; found 566.2208.

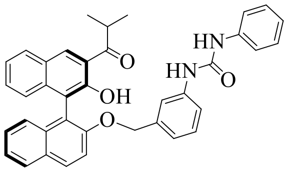

(*R*)-**4**: yield 38%. $[\alpha]_D$ = 117.5 (*c* 0.4, $CHCl_3$). ^{1}H NMR (300 MHz, $CDCl_3$) δ 12.12 (s, 1H), 8.52 (s, 1H), 6.81~7.87 (m, 13H), 6.71 (s, 1H), 6.59 (d, 1H), 4.85 (s, 2H), 3.87 (m, 1H), 1.25-1.37 (d, J = 6 Hz, 6H). ^{13}C NMR (75 MHz, $CDCl_3$) δ 211.7, 155.0, 153.9, 153.3, 138.2, 137.6, 133.6, 129.8, 124.9, 119.5, 115.4, 70.7, 35.4, 19.8, 19.5. HRMS-EI (*m/z*) calcd for $C_{38}H_{32}N_2O_4$ 580.2362; found 580.2362.

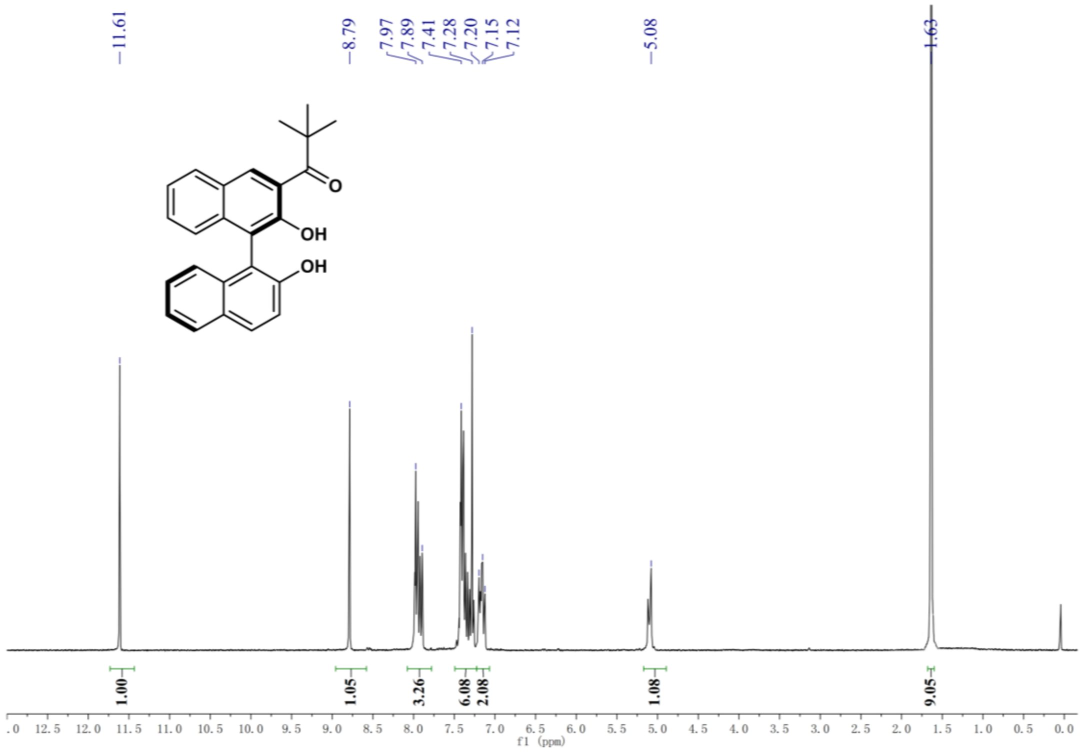

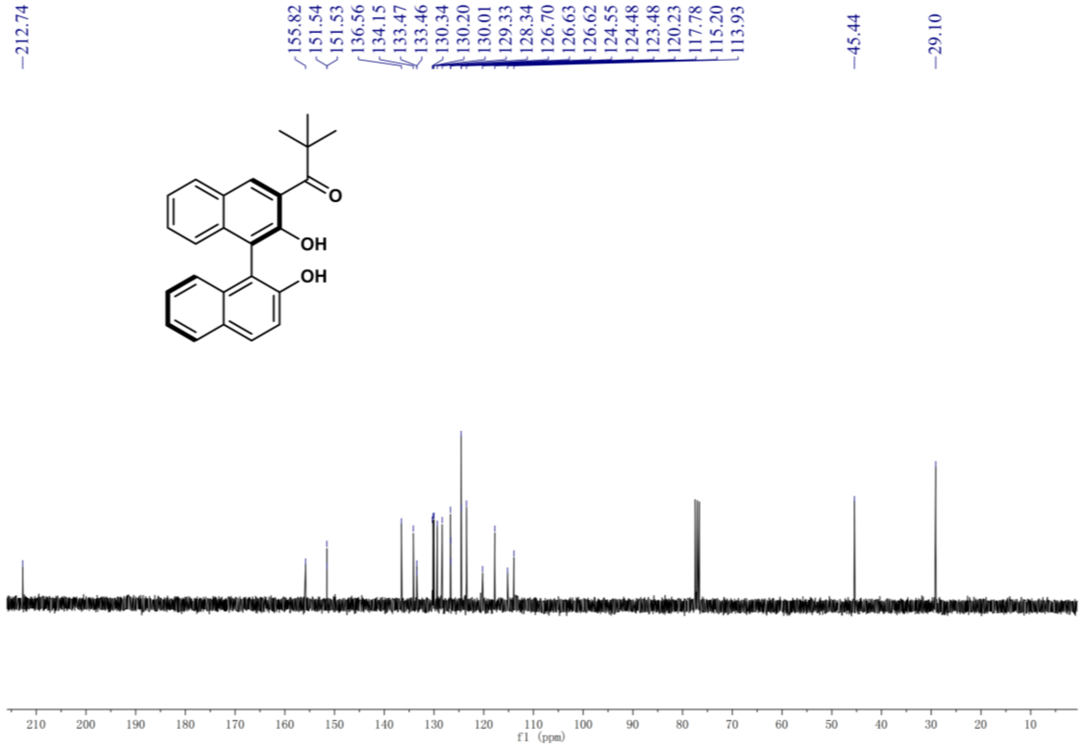

Supplementary Figure 1. ^{1}H and ^{13}C NMR spectra of (*R*)-3-*tert*-butyl-2, 2′-dihydroxy-1,1′-binaphthyl ketone in $CDCl_3$.

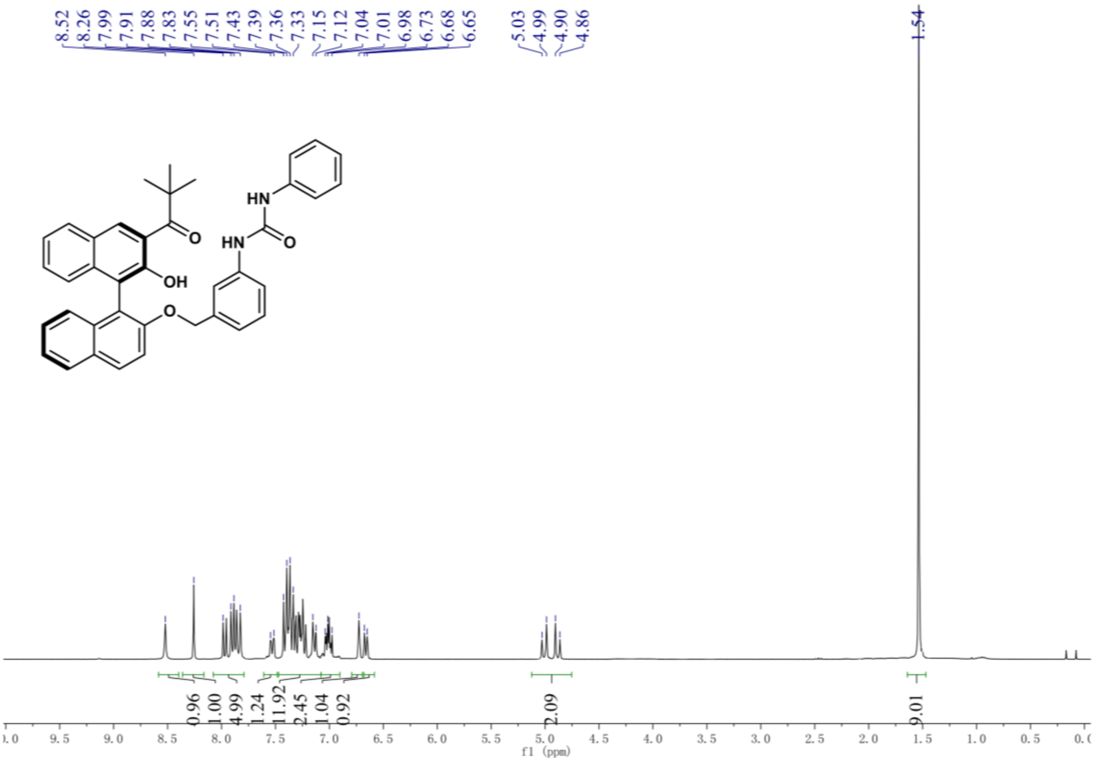

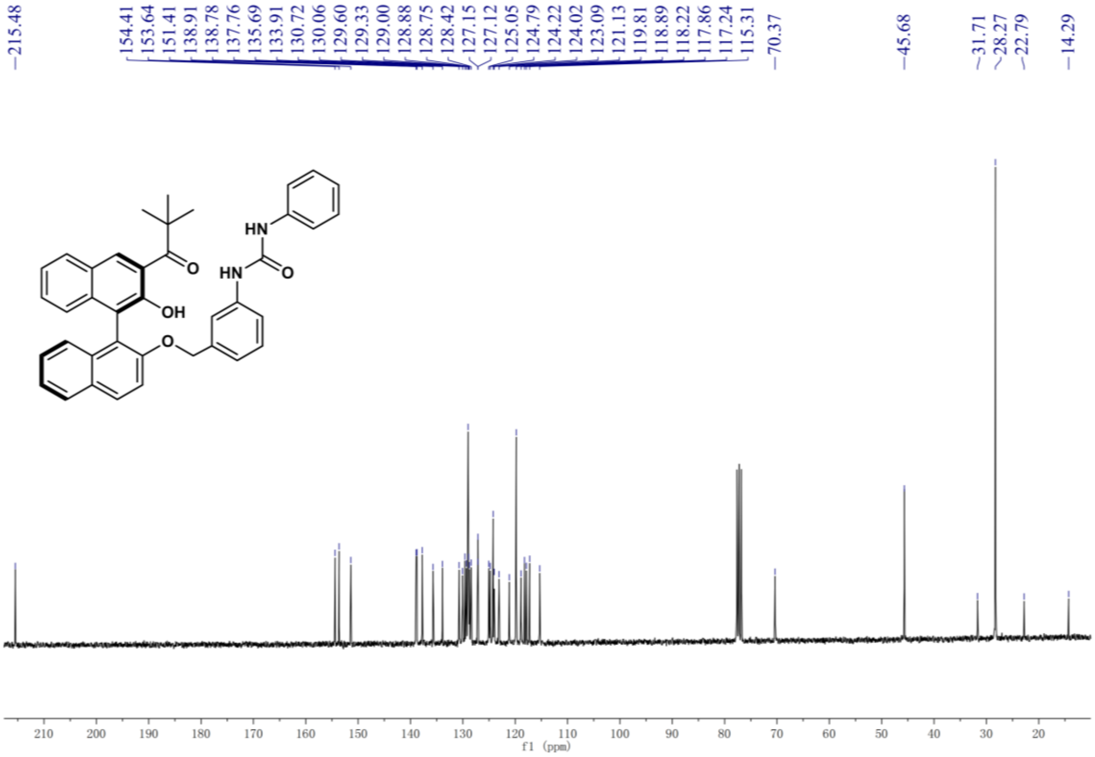

Supplementary Figure 2. ^{1}H and ^{13}C NMR spectra of (*R*)-**5** in $CDCl_3$.

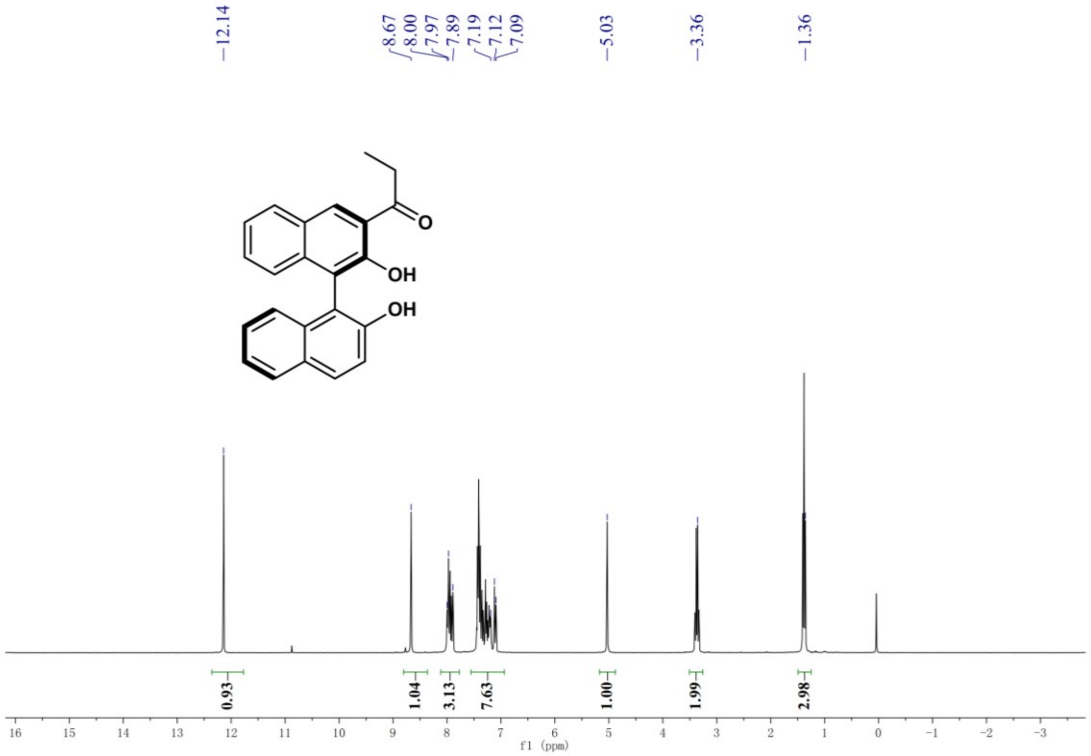

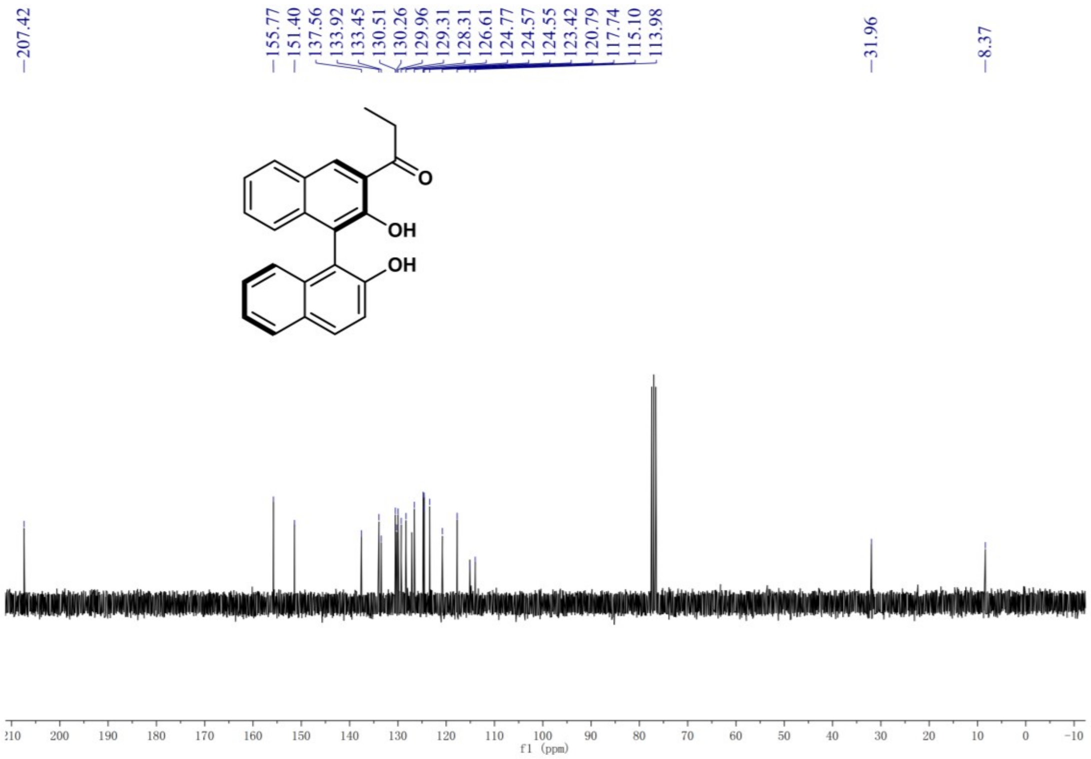

Supplementary Figure 3. ^{1}H and ^{13}C NMR spectra of (*R*)-3-ethyl-2,2′-dihydroxy-1,1′-binaphthyl ketone in $CDCl_3$.

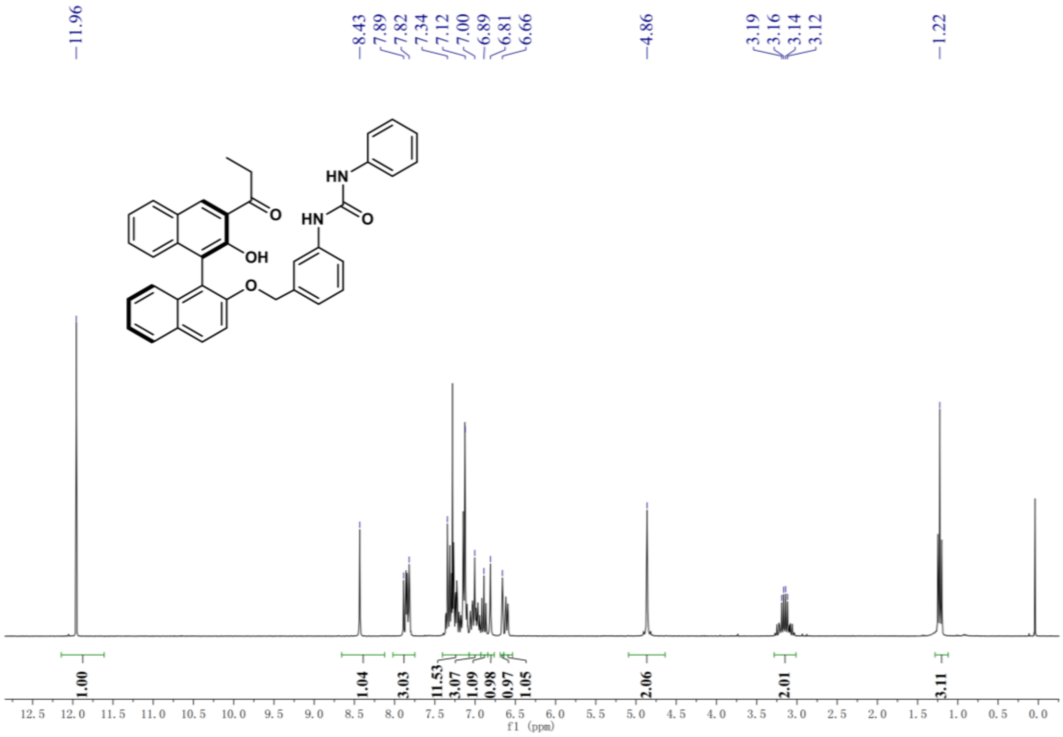

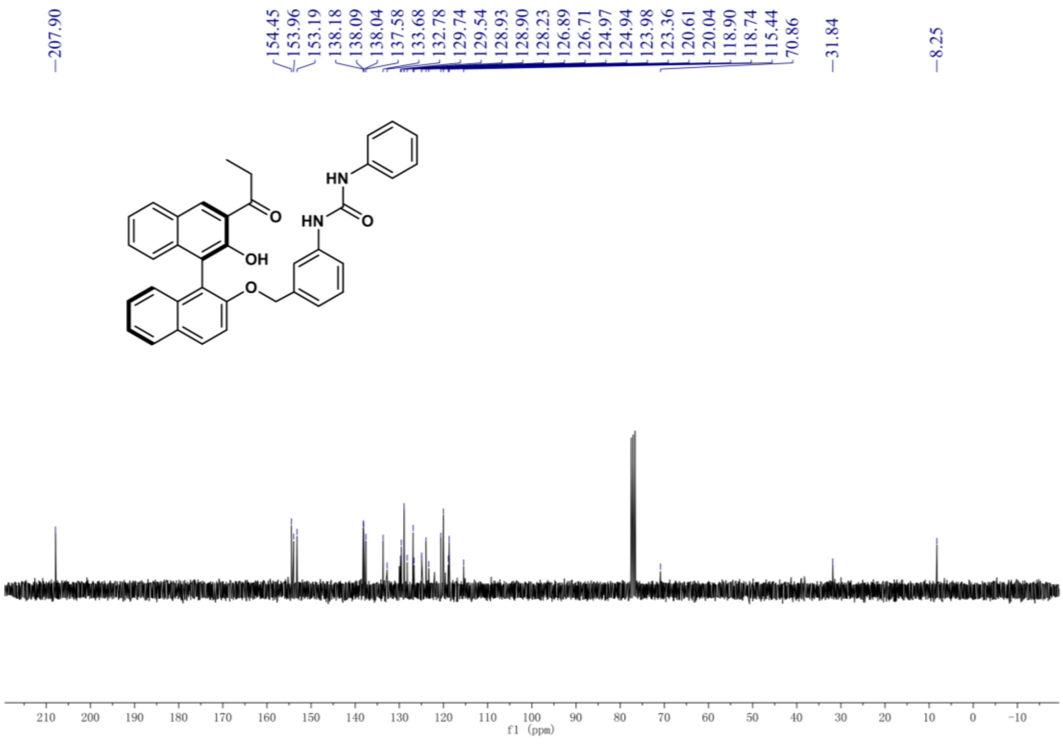

Supplementary Figure 4. ^{1}H and ^{13}C NMR spectra of (*R*)-**3** in $CDCl_3$.

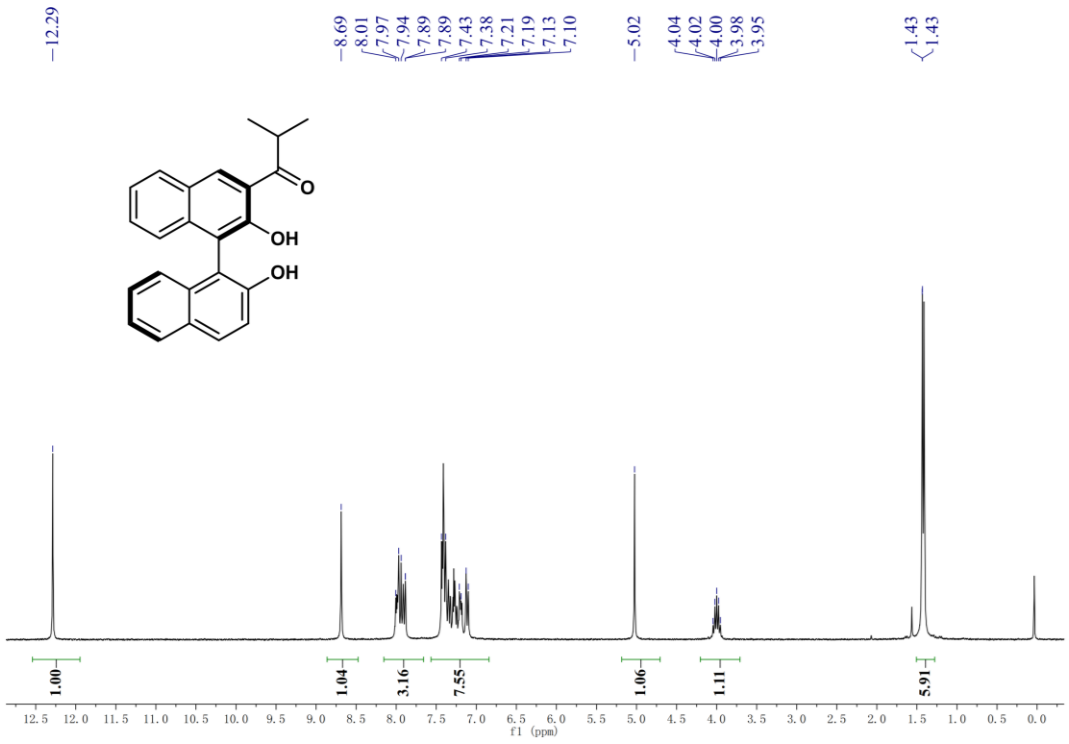

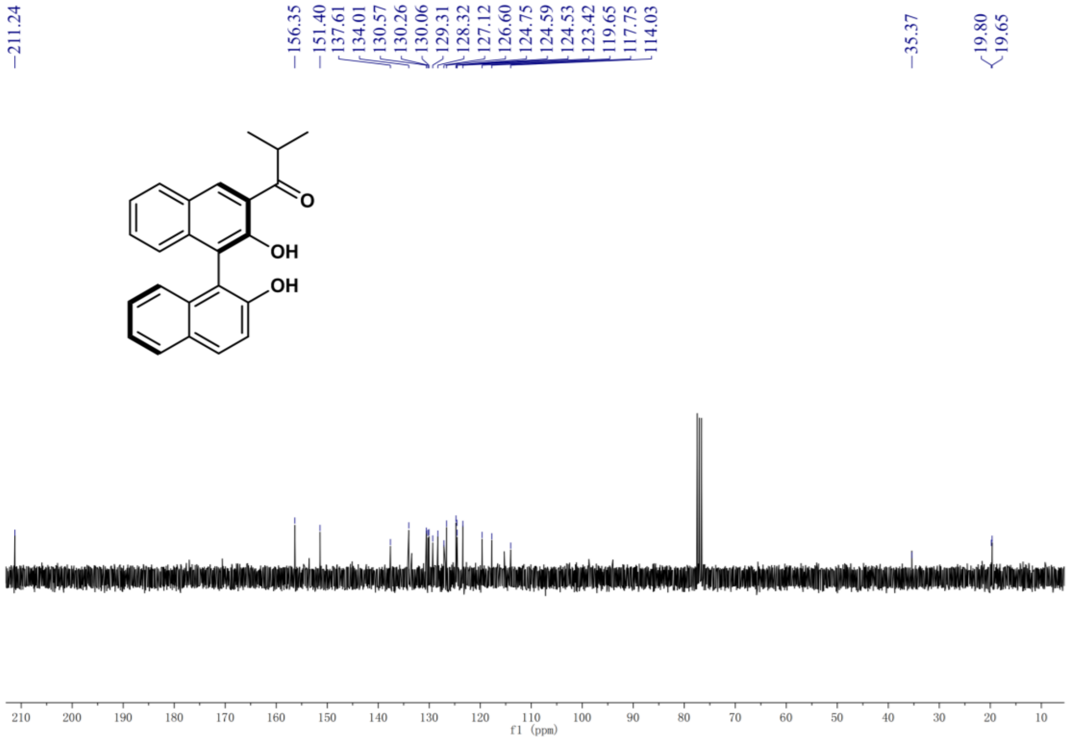

Supplementary Figure 5. ^{1}H and ^{13}C NMR spectra of (*R*)-3-isopropyl-2,2′-dihydroxy-1,1′-binaphthyl ketone in $CDCl_3$.

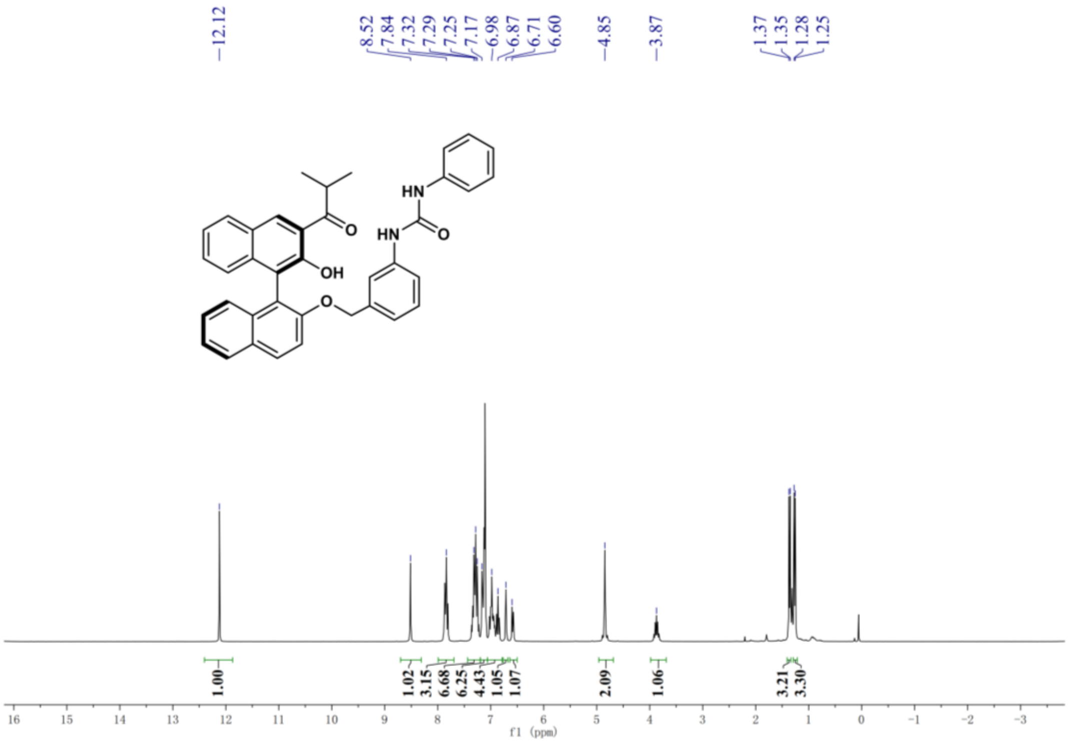

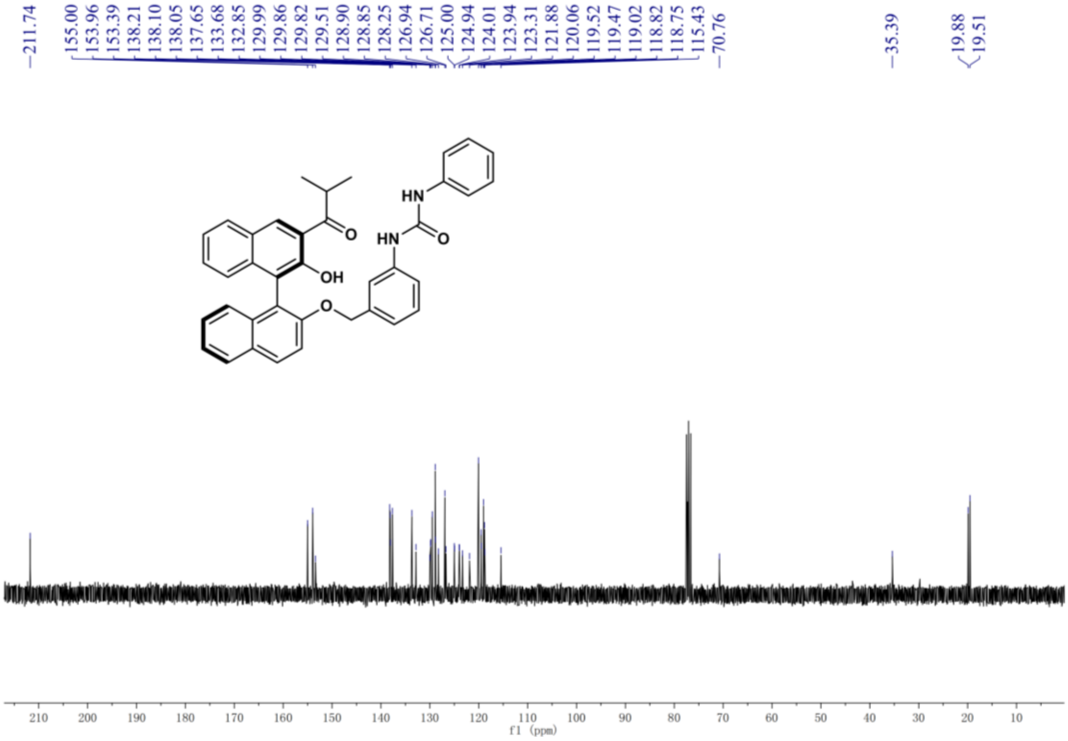

Supplementary Figure 6. ^{1}H and ^{13}C NMR spectra of (*R*)-**4** in $CDCl_3$.

2. Extraction of phenylalanine with (*R*)-5

2.1 Typical ELLE procedure

An organic layer prepared by the dissolution of (*R*)-**5** (0.60 g, 1 mmol) and Aliquat-336 (0.43 g, 1.05 mmol) in $CHCl_3$ (2 mL) was added to an aqueous layer prepared by the addition of a DL-amino acid (4 mmol) to a solution of NaOH (0.16 g, 4 mmol) in water (2 mL). The two layers were mixed and stirred vigorously at room temperature (20-25 °C). The stirring was continued until imine formation was complete, as monitored by ^{1}H-NMR analysis of the organic layer.

2.2 Details for extraction of DL-Phe, D-Phe, and L-Phe with (*R*)-5

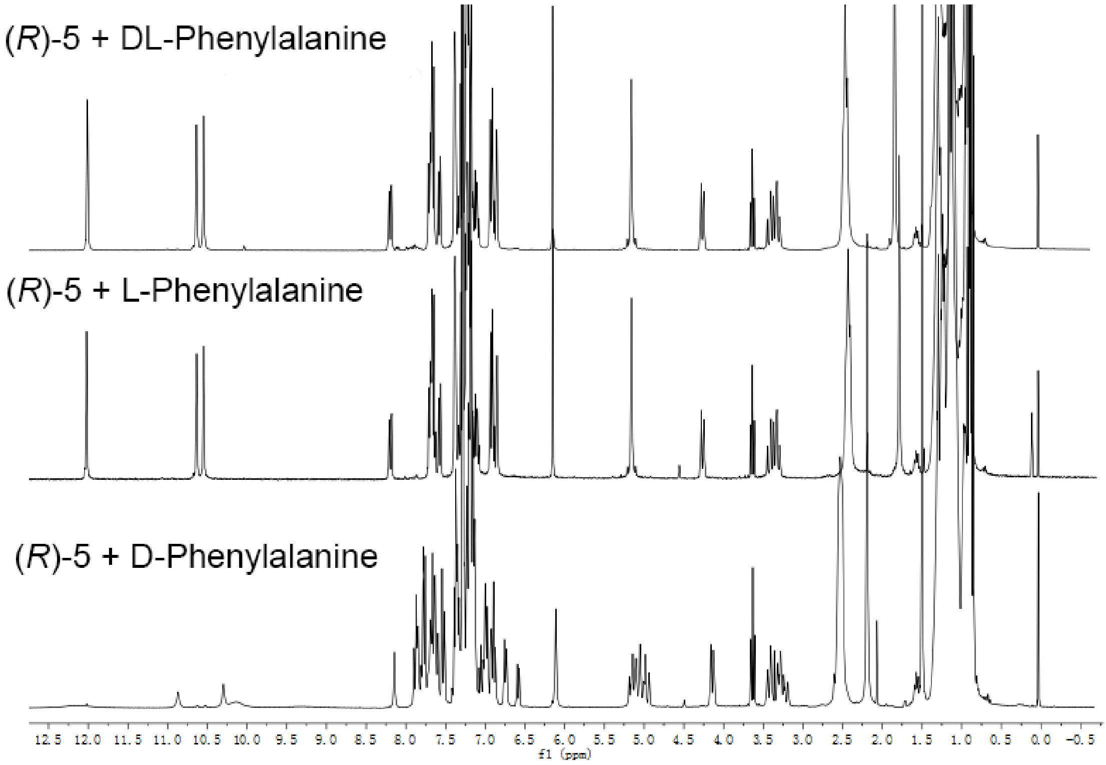

Supplementary Figure 7. ^{1}H NMR spectra of the organic layer after completion of the ELLE experiment using (*R*)-**5** with DL-Phe (top), L-Phe (middle), and L-Phe (bottom), respectively.

(a)

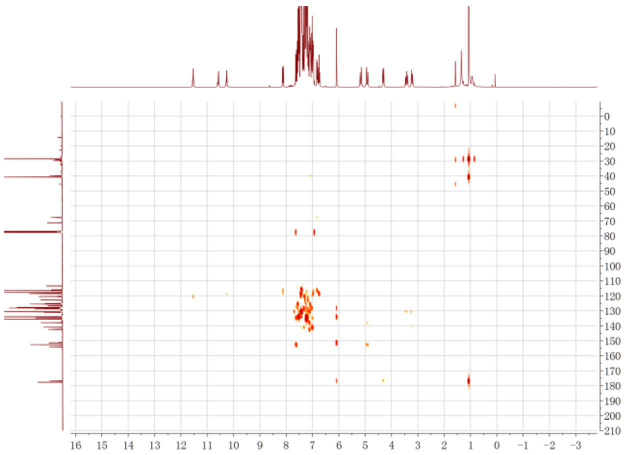

(b)

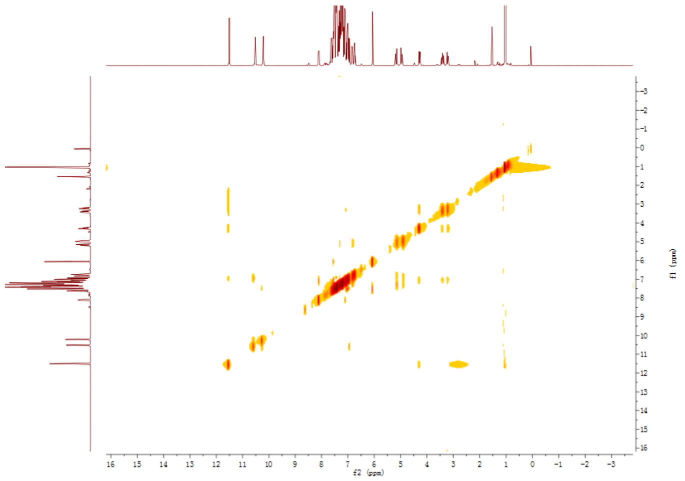

(c)

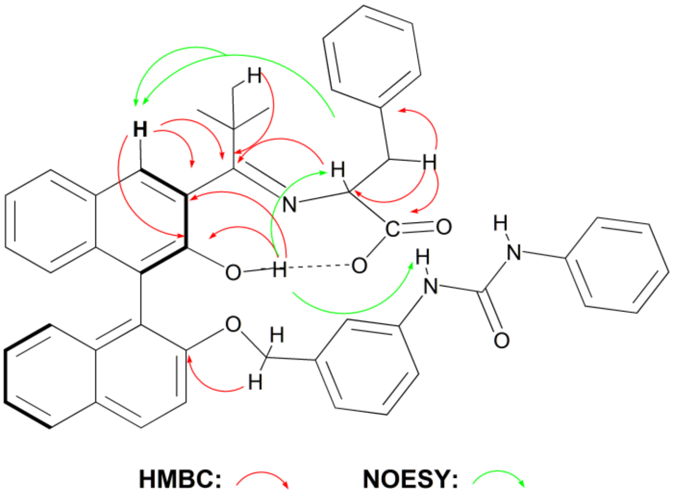

Supplementary Figure 8. (a) HMBC and **(b)** NOESY spectra of the imine (*R*)-**5**-L-Phe. **(c)** Schematic representation of the H-H and C-H correlations in the imine structure according to the above 2D NMR data.

3. Comparative data for the extraction of phenylalanine with (*R*)-1~5

The experiments were carried out following the typical ELLE procedure (see Supplementary section 2.1) using the chiral extractants (*R*)-**1-5**. After completion, the organic layers were analyzed by ^{1}H-NMR.

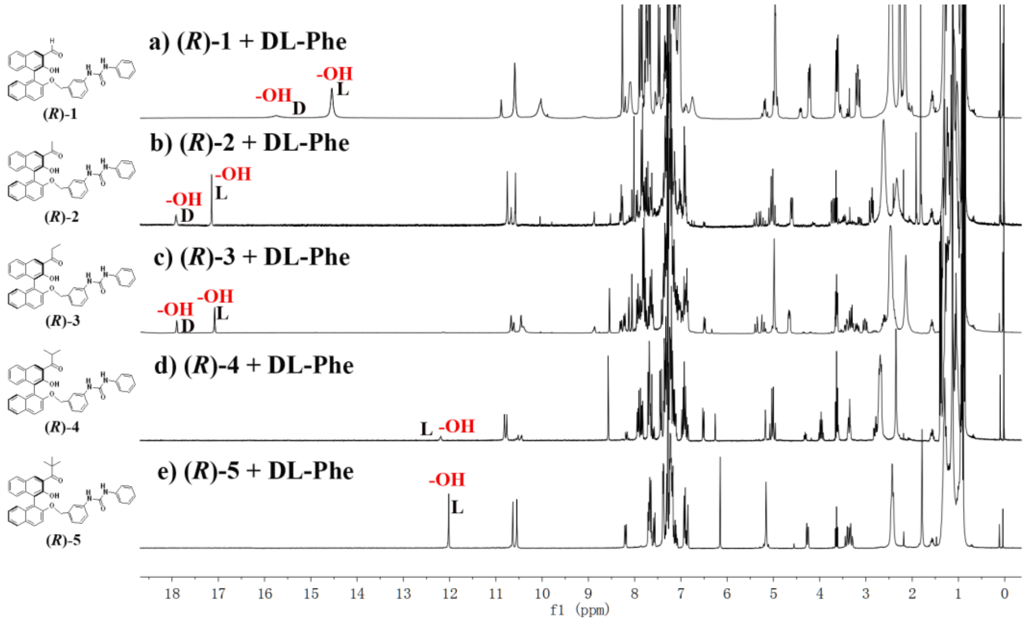

Supplementary Figure 9. Full width ^{1}H NMR spectra of the organic layers after completion of the ELLE experiments in the extraction of DL-Phe with a) (*R*)-**1**, b) (*R*)-**2**, c) (*R*)-**3**, d) (*R*)-**4**, and e) (*R*)-**5**, respectively.

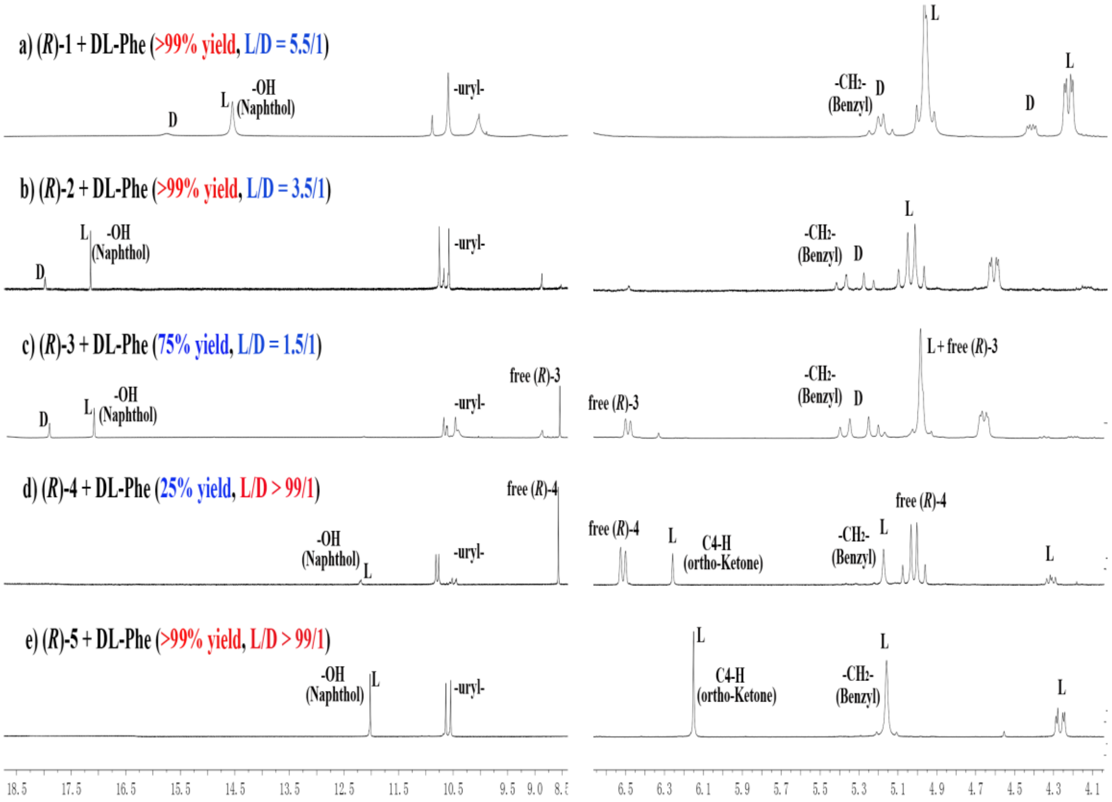

Supplementary Figure 10. Magnified regions of the ^{1}H NMR spectra presented in Supplementary Figure 9 of the organic layers after completion of the ELLE experiments in the extraction of DL-Phe with a) (*R*)-**1**, b) (*R*)-**2**, c) (*R*)-**3**, d) (*R*)-**4**, and e) (*R*)-**5**, respectively. The imine yields and diastereomeric ratios (L/D) were determined by comparative integration.

4. Crystallographic data for [(*R*)-**5**-L-Phe]$^-$[TPP]$^+$

Supplementary Table 1. Crystallographic data and structure refinement for (*R*)-**5**-L-Phe (tetraphenylphosphonium salt).*

_

Empirical formula	C76 H66 N3 O5 P	
Formula weight	1132.28	
Temperature	296(2) K	
Wavelength	0.71073 Å	
Crystal system	Triclinic	
Space group	P 1	
Unit cell dimensions	a = 10.4978(2) Å	α= 114.6704(10)°.
	b = 13.0490(2) Å	β= 100.7775(11)°.
	c = 13.8968(3) Å	γ = 101.0028(11)°.
Volume	1620.45(5) $Å^3$	
Z	1	
Density (calculated)	1.160 Mg/m^3	
Absorption coefficient	0.095 mm^{-1}	
F(000)	598	
Crystal size	0.200 x 0.200 x 0.100 mm^3	
Theta range for data collection	1.690 to 24.152°.	
Index ranges	-12<=h<=12, -15<=k<=15, -15<=l<=15	
Reflections collected	43005	
Independent reflections	10296 [R(int) = 0.0386]	
Completeness to theta = 25.242°	87.9 %	
Absorption correction	multi-scan	
Refinement method	Full-matrix least-squares on F^2	
Data / restraints / parameters	10296 / 113 / 816	
Goodness-of-fit on F^2	1.046	
Final R indices [I>2sigma(I)]	R1 = 0.0601, wR2 = 0.1703	
R indices (all data)	R1 = 0.0723, wR2 = 0.1845	
Absolute structure parameter	0.03(3)	
Largest diff. peak and hole	0.827 and -0.206 e.$Å^{-3}$	

* CCDC Deposition Number for [(*R*)-**5**-L-Phe]$^-$[TPP]$^+$: 1948381

5. ELLE and back-extraction with (*R*)-5

5.1. Extraction with (*R*)-5 for representative amino acids

The experiments were carried out following the typical ELLE procedure (see Supplementary Section 2.1) using the chiral extractants (*R*)-**5**. After completion, the organic layers were analyzed by ^{1}H-NMR. Then, the imines were hydrolyzed by stirring the organic layer in the presence of aqueous 2N HCl (1.5 mL), and the purity of the back-extracted amino acids was assayed by chiral HPLC.

(a)

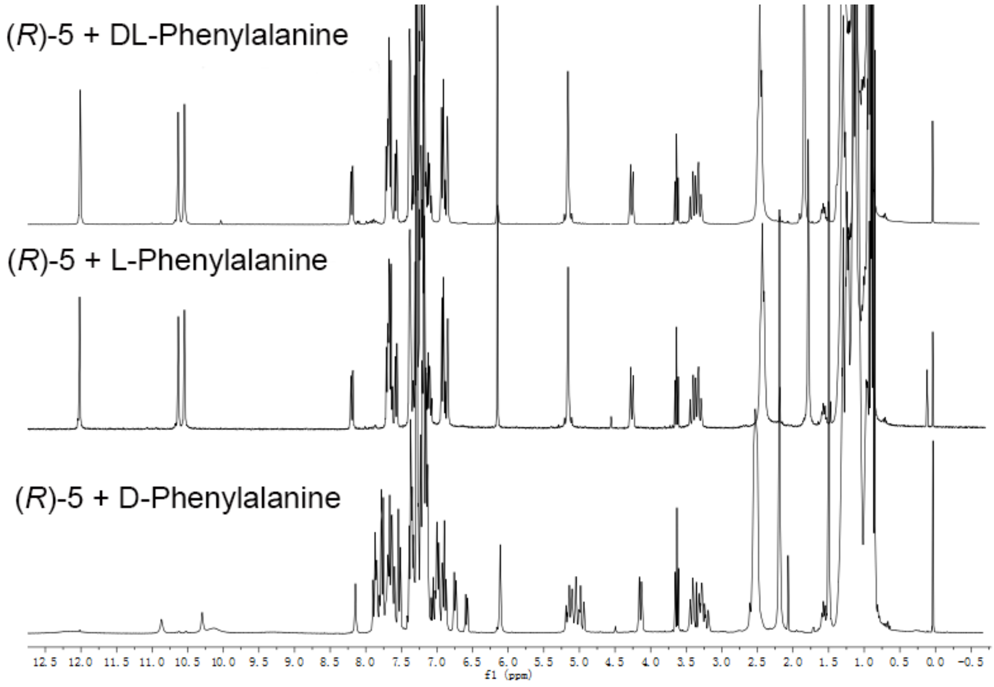

Supplementary Figure 11-(a). ^{1}H NMR spectra of the organic layer after completion of the ELLE experiment using (*R*)-**5** with DL-Phe (top), L-Phe (middle), and L-Phe (bottom), respectively.

(b)

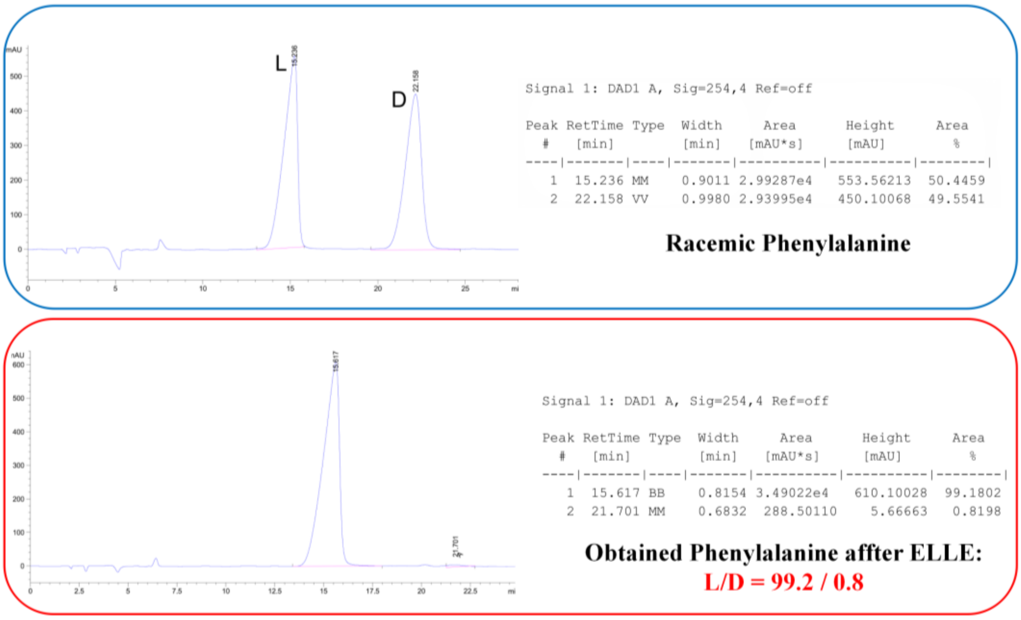

Supplementary Figure 11-(b). HPLC chromatograms of racemic phenylalanine (blue box, top) compared to that of the product isolated after ELLE and subsequent hydrolysis (red box, below). **HPLC conditions**: Column: Sumichiral OA-5000, S.A.S. Co.; Eluent: 2 mM $CuSO_4$ solution/2-propanol (85/15); Flow rate: 1 mL/min; Detector: UV 254 nm.

(a)

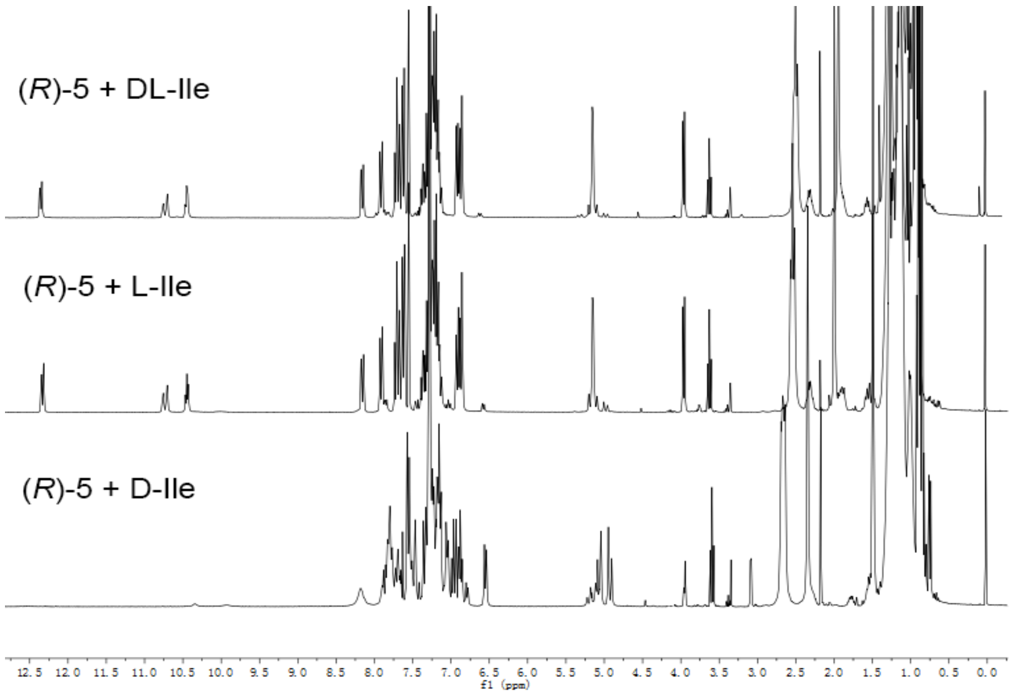

(b)

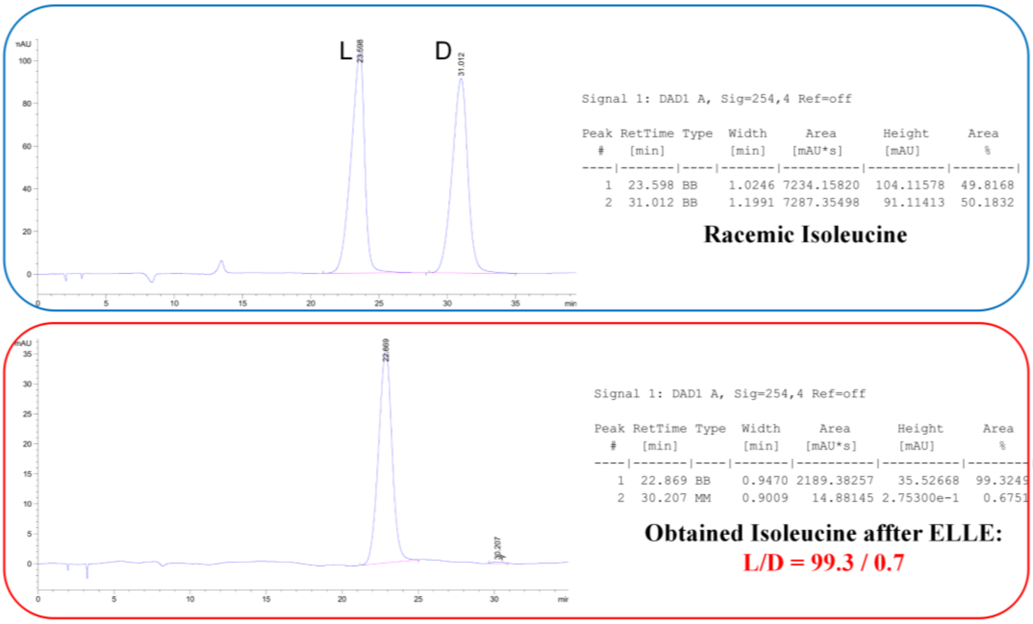

Supplementary Figure 12. (a) ^{1}H NMR spectra of the organic layer after completion of the ELLE experiment using (*R*)-**5** with DL-Ile (top), L-Ile (middle), and L-Ile (bottom), respectively. **(b)** HPLC chromatograms of racemic isoleucine (blue box, top) compared to that of the product isolated after ELLE and subsequent hydrolysis (red box, below). **HPLC conditions**: Column: Sumichiral OA-5000, S.A.S. Co.; Eluent: 2mM $CuSO_4$ solution/2-propanol (95/5); Flow rate: 1 mL/min; Detector: UV 254nm.

(a)

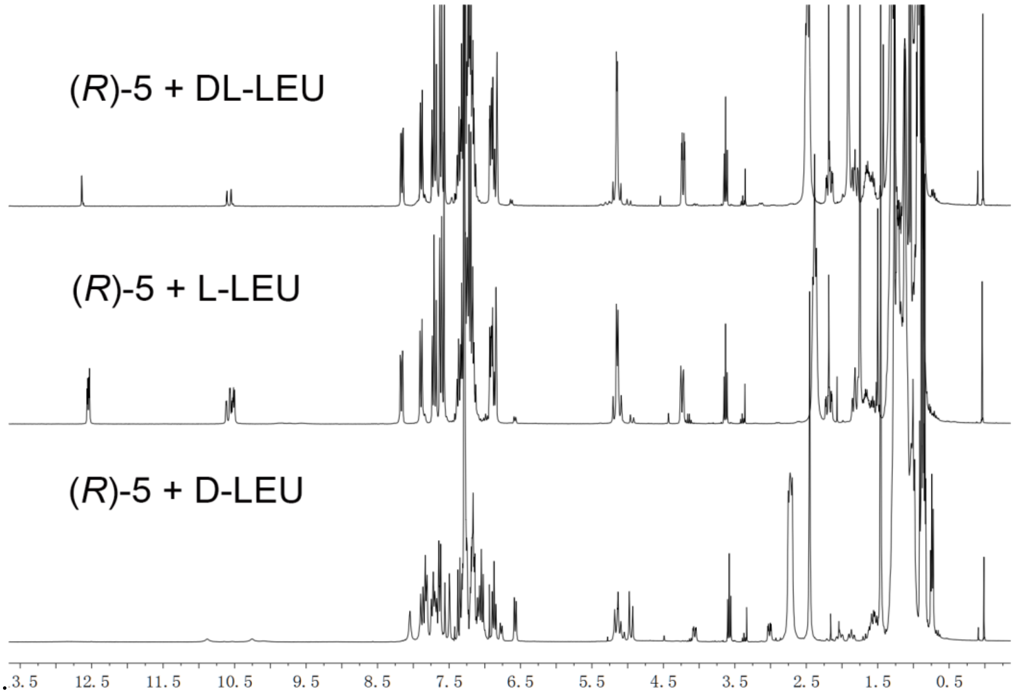

(b)

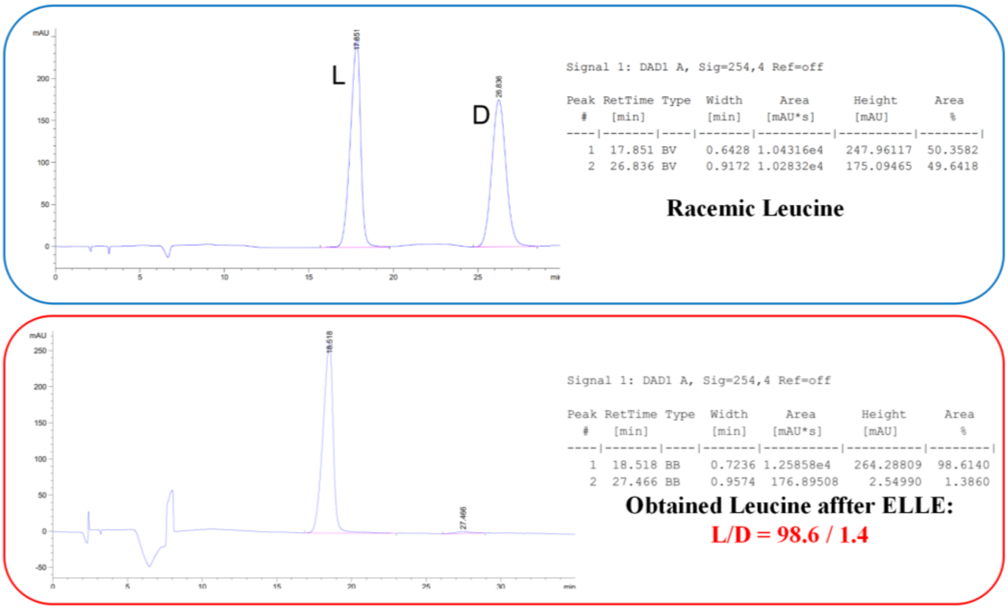

Supplementary Figure 13. (a) ^{1}H NMR spectra of the organic layer after completion of the ELLE experiment using (*R*)-**5** with DL-Leu (top), L-Leu (middle), and L-Leu (bottom), respectively. **(b)** HPLC chromatograms of racemic leucine (blue box, top) compared to that of the product isolated after ELLE and subsequent hydrolysis (red box, below). **HPLC conditions**: Column: Sumichiral OA-5000, S.A.S. Co.; Eluent: 2mM $CuSO_4$ solution/2-propanol (95/5); Flow rate: 1 mL/min; Detector: UV 254 nm.

(a)

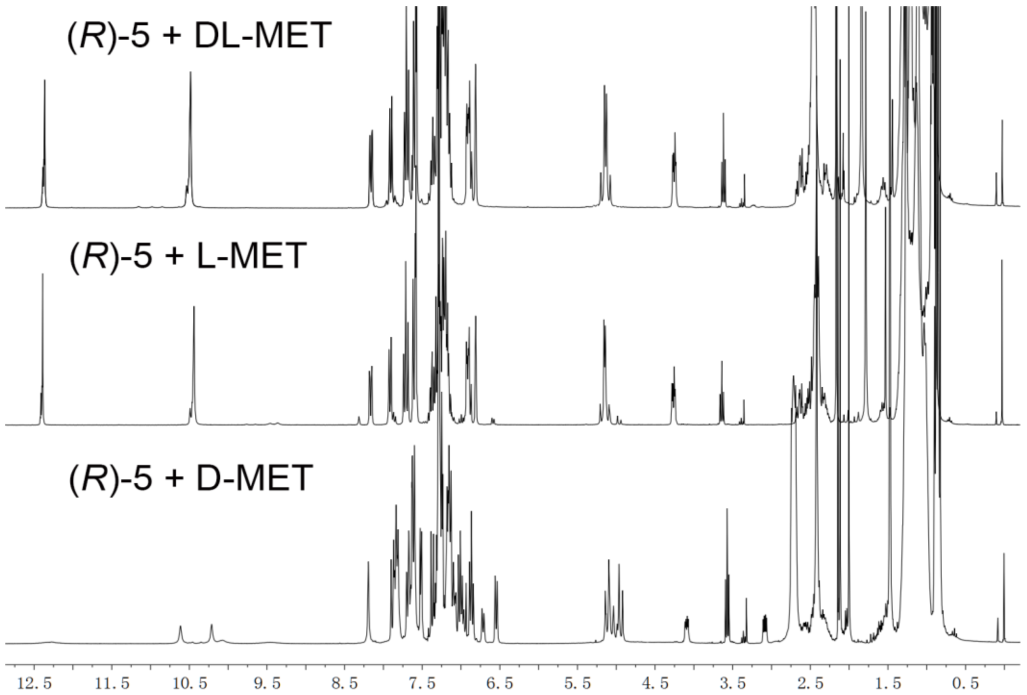

(b)

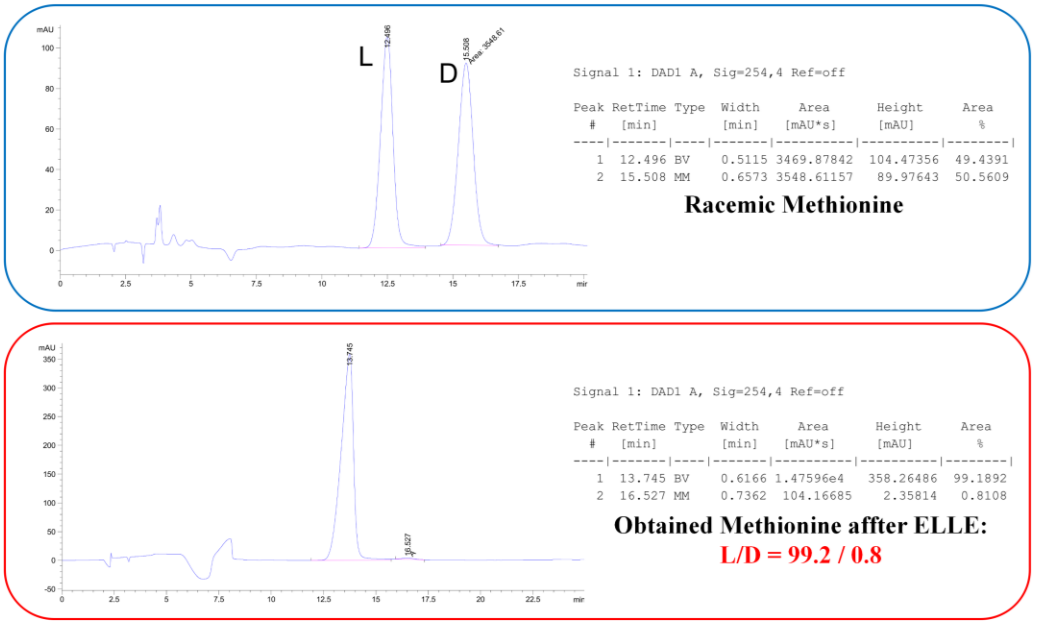

Supplementary Figure 14. (a) ^{1}H NMR spectra of the organic layer after completion of the ELLE experiment using (*R*)-**5** with DL-Met (top), L-Met (middle), and L-Met (bottom), respectively. **(b)** HPLC chromatograms of racemic methionine (blue box, top) compared to that of the product isolated after ELLE and subsequent hydrolysis (red box, below). **HPLC conditions**: Column: Sumichiral OA-5000, S.A.S. Co.; Eluent: 2mM $CuSO_4$ solution/2-propanol (95/5); Flow rate: 1 mL/min; Detector: UV 254 nm.

(a)

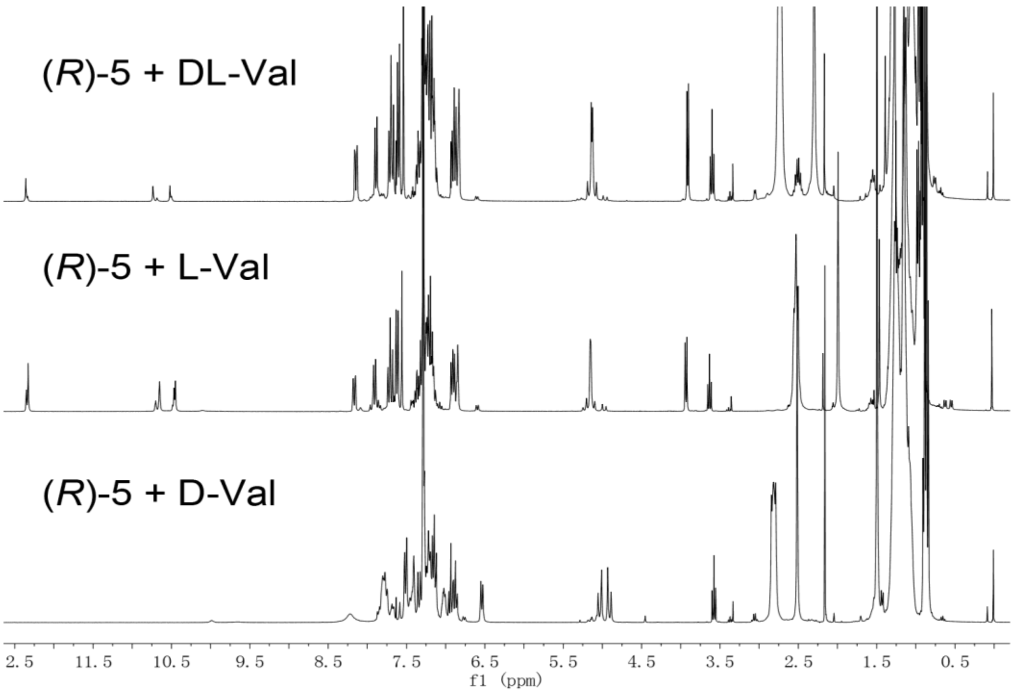

(b)

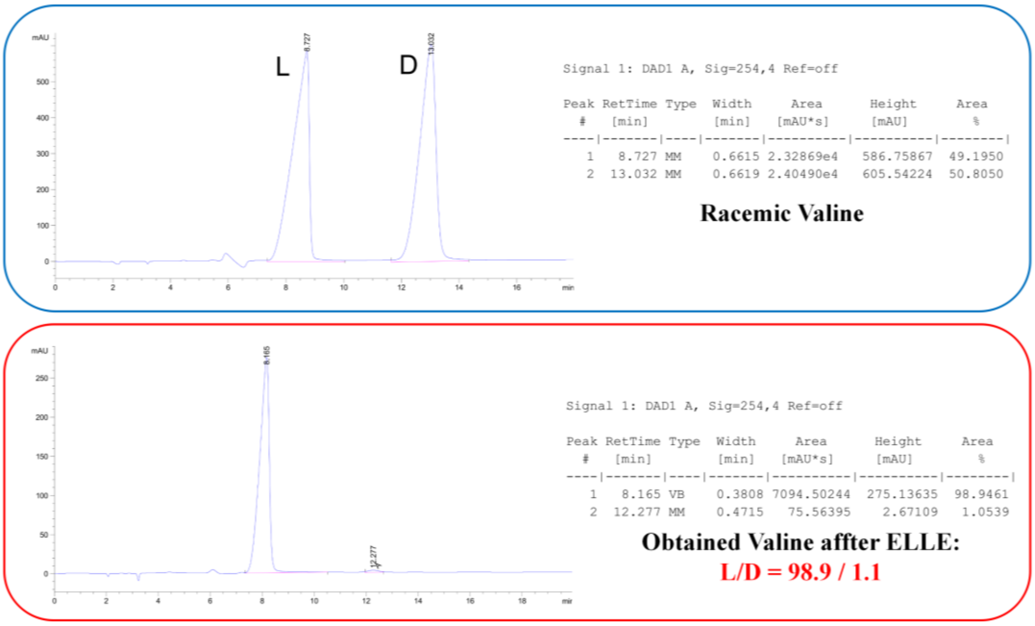

Supplementary Figure 15. (a) ^{1}H NMR spectra of the organic layer after completion of the ELLE experiment using (*R*)-**5** with DL-Val (top), L-Val (middle), and L-Val (bottom), respectively. **(b)** HPLC chromatograms of racemic valine (blue box, top) compared to that of the product isolated after ELLE and subsequent hydrolysis (red box, below). **HPLC conditions**: Column: Sumichiral OA-5000, S.A.S. Co.; Eluent: 2mM $CuSO_4$ solution/2-propanol (95/5); Flow rate: 1 mL/min; Detector: UV 254 nm.

(a)

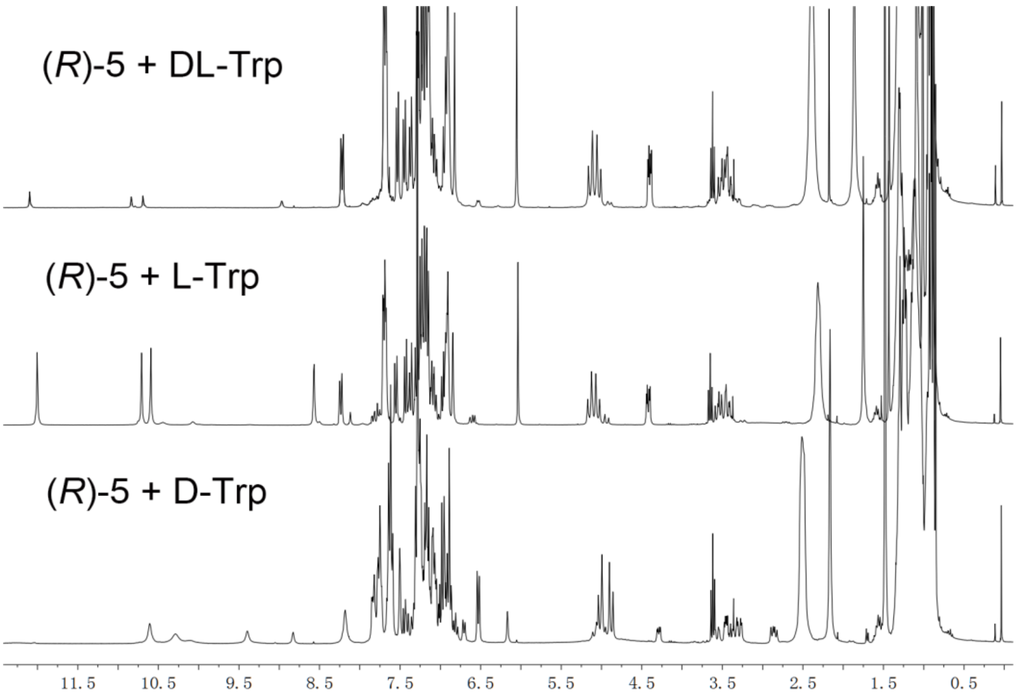

(b)

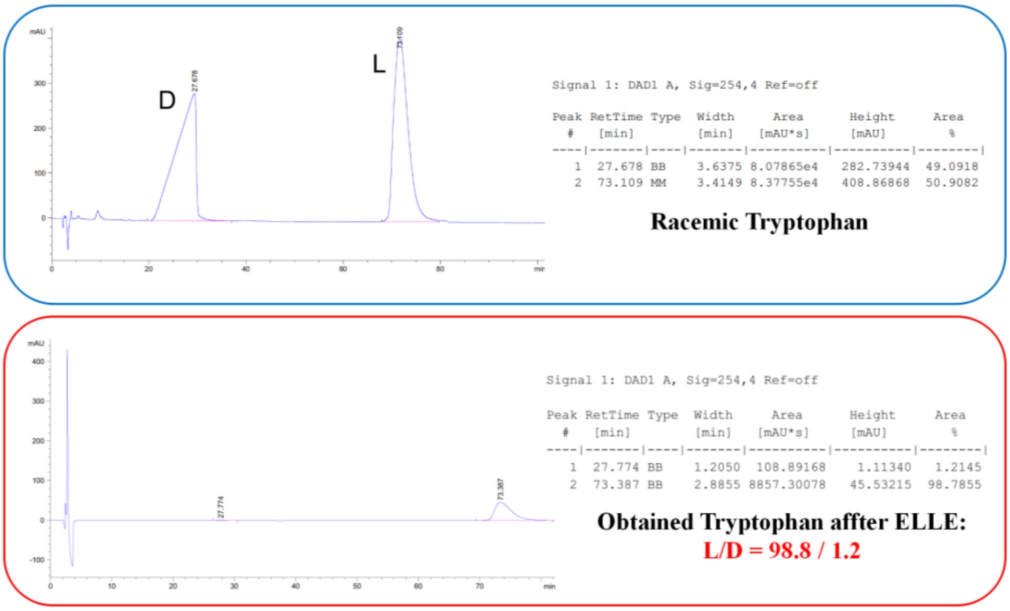

Supplementary Figure 16. (a) ^{1}H NMR spectra of the organic layer after completion of the ELLE experiment using (*R*)-**5** with DL-Trp (top), L-Trp (middle), and L-Trp (bottom), respectively. **(b)** HPLC chromatograms of racemic tryptophan (blue box, top) compared to that of the product isolated after ELLE and subsequent hydrolysis (red box, below). **HPLC conditions**: Column: Sumichiral OA-5000, S.A.S. Co.; Eluent: 2mM $CuSO_4$ solution/acetonitrile (90/10); Flow rate: 1 mL/min; Detector: UV 254 nm.

(a)

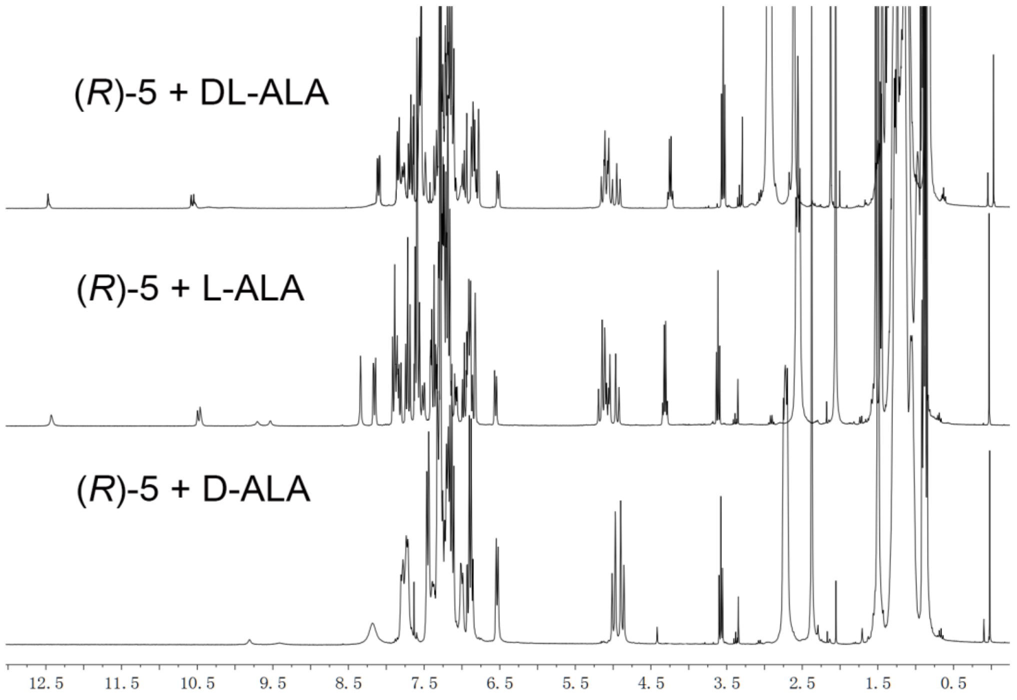

(b)

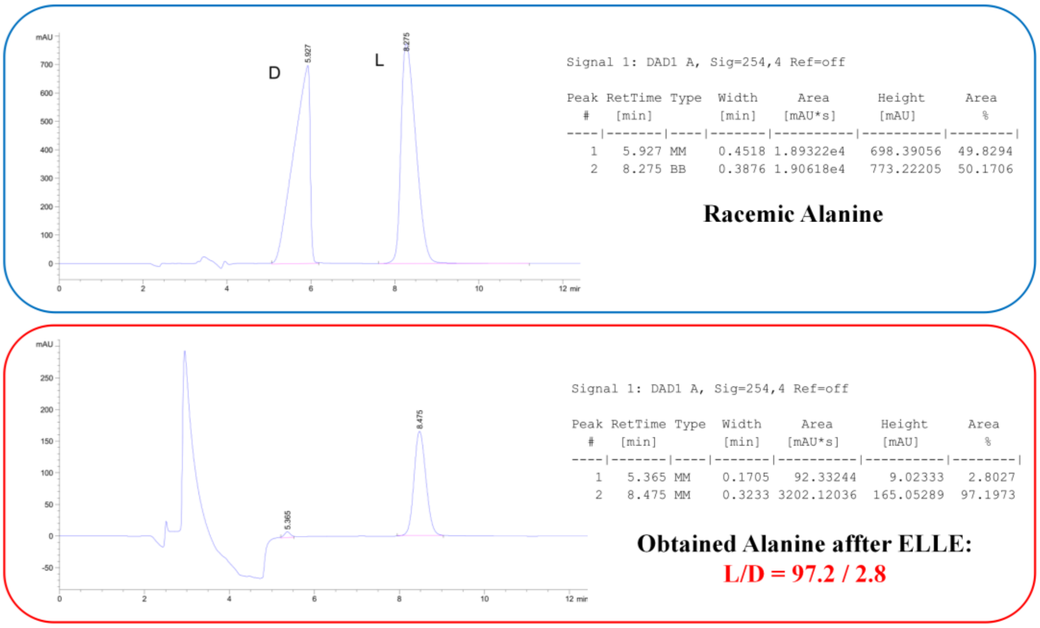

Supplementary Figure 17. (a) ^{1}H NMR spectra of the organic layer after completion of the ELLE experiment using (*R*)-**5** with DL-Ala (top), L-Ala (middle), and L-Ala (bottom), respectively. **(b)** HPLC chromatograms of racemic alanine (blue box, top) compared to that of the product isolated after ELLE and subsequent hydrolysis (red box, below). **HPLC conditions**: Column: Sumichiral OA-5000, S.A.S. Co.; Eluent: 2mM $CuSO_4$ solution/2-propanol (95/5); Flow rate: 1 mL/min; Detector: UV 254 nm.

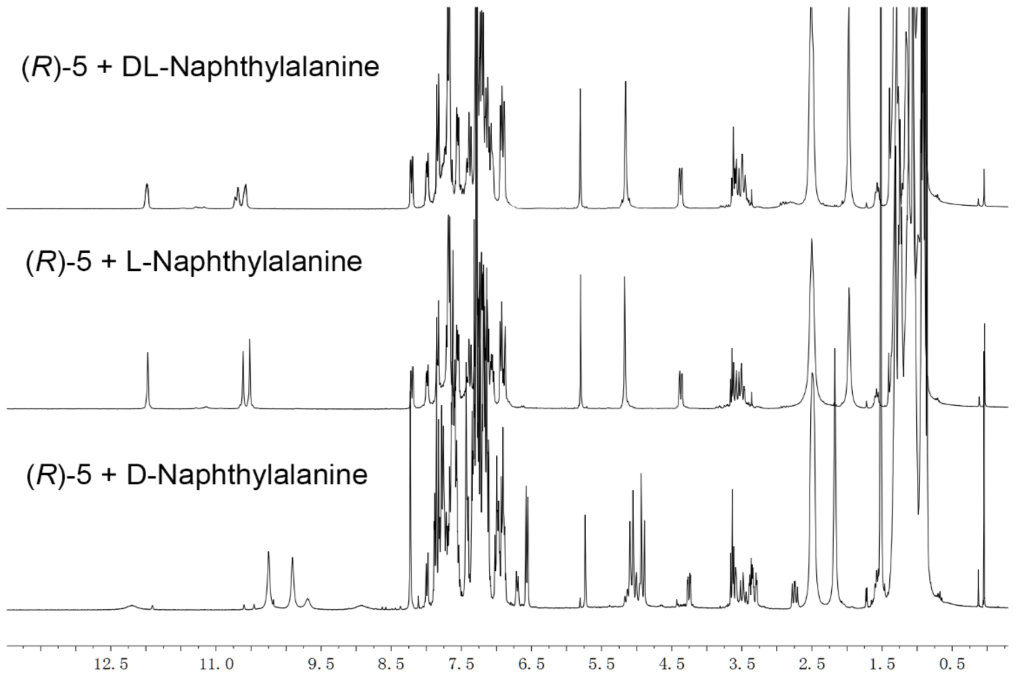

Supplementary Figure 18. (a) ^{1}H NMR spectra of the organic layer after completion of the ELLE experiment using (*R*)-**5** with DL-Nal (top), L-Nal (middle), and L-Nal (bottom), respectively.

5.2 Robustness and recyclability of the extractant (*R*)-5

An experiment was performed to establish the robustness and recyclability of the chiral extractant (*R*)-**5** during the course of ELLE experiments. The ELLE of DL-Phe was performed, recycling the organic layer containing (*R*)-**5** and Aliquat 336 after acid hydrolysis, over 20 cycles. The composition of the organic layer was analyzed by ^{1}H-NMR, as shown in Supplementary Figure 19 shown below. No sign of degradation or decomposition of (*R*)-**5** could be detected over the course of the 20 ELLE cycles.

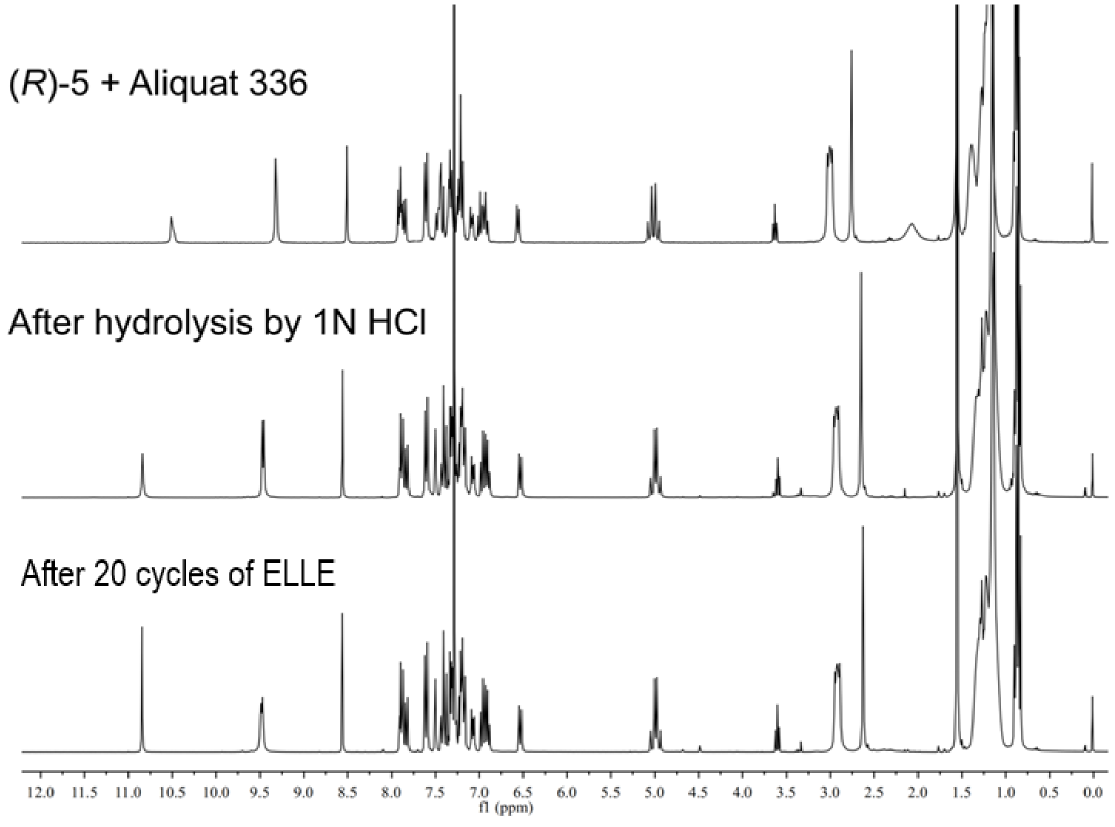

Supplementary Figure 19. ^{1}H NMR spectra of the organic layer in the ELLE of DL-Phe with (*R*)-**5**: before the first extraction cycle (top), following the first extraction and acid hydrolysis cycle (middle), and following the completion of 20 ELLE cycles (bottom).

5.3 Hydrolysis (back-extraction) kinetics

The kinetics of back-extraction of Phe was tested using either 1 eq 2 N HCl, or 2 eq 2N HCl aqueous solutions. The results are shown in Supplementary Figure 20.

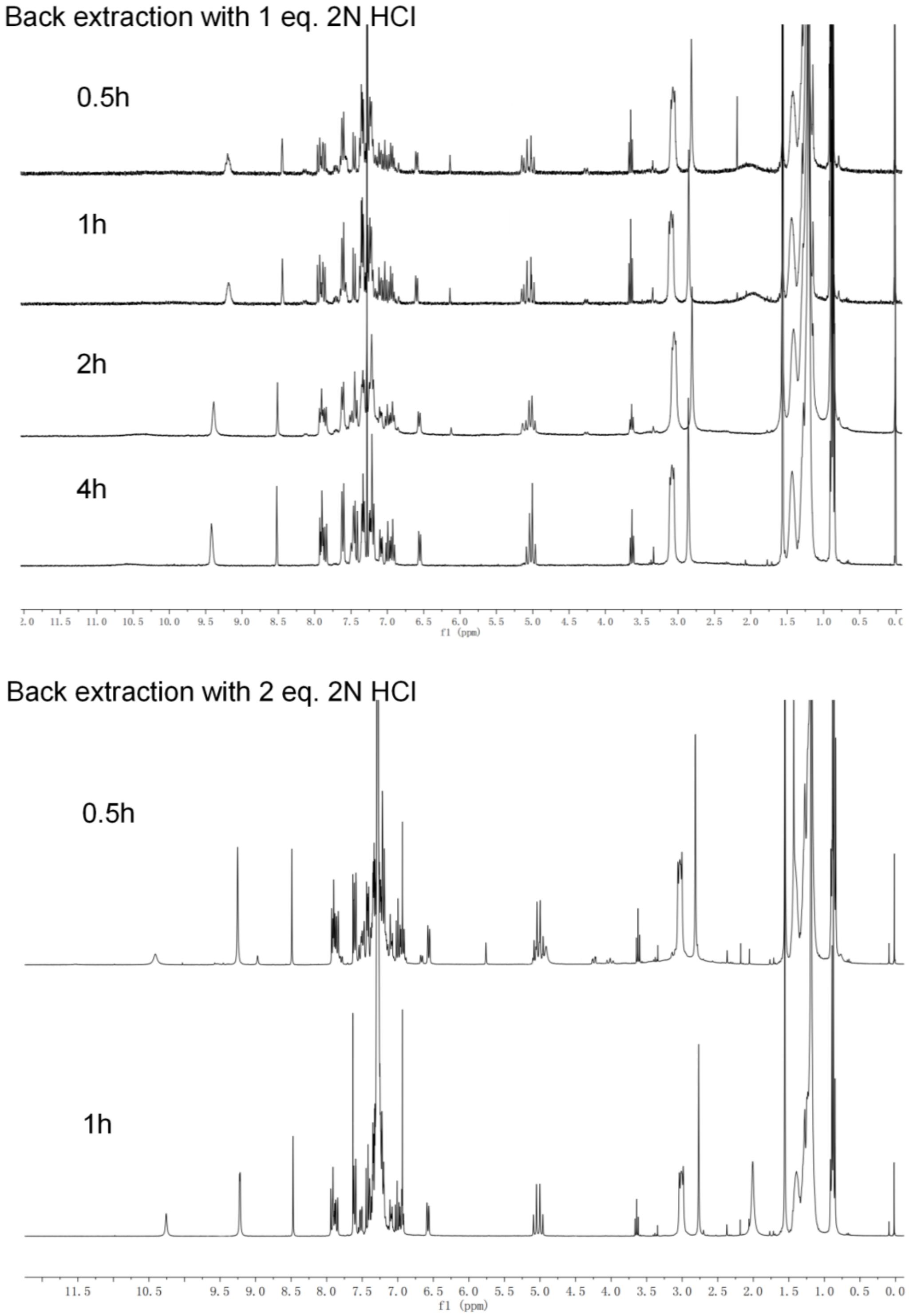

Supplementary Figure 20. ^{1}H NMR for the separated organic layer obtained in the back-extraction experiment of (*R*)-5-L-Phe after stirring for the indicated time in the presence of aqueous HCl solutions. The results show that 2 h is sufficient to complete the hydrolysis of the imine and fully back-extract the amino acids.

6. EECR with (*R*)-5 for representative amino acids

6.1 Racemization of amino acids in aqueous layer.

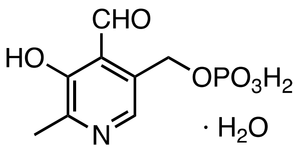

PLP
Pyridoxal-5'-phosphate monohydrate

The successful racemization of amino acids in the aqueous layer is a critical factor to achieve EECR with acceptable performance and timescale. We found that small catalytic amounts of PLP and Cu^{2+} ions were sufficient to catalyze the racemization of amino acids in the aqueous layer at acceptable rates. **Supplementary Figure 21** shows representative HPLC kinetics data for the racemization of phenylalanine over 26h, starting with L-Phe (1.0 M), PLP (0.01 M) and Cu^{2+} (0.01 M) at room temperature. No significant amino acid degradation/decomposition side products were detected by HPLC. **Supplementary Figure 22** shows ^{1}H NMR monitoring of the aqueous phenylalanine solution in the presence of the racemization catalyst under the above conditions. It should be noted again that no significant amino acid degradation/decomposition side products were detected by ^{1}H NMR under these conditions.

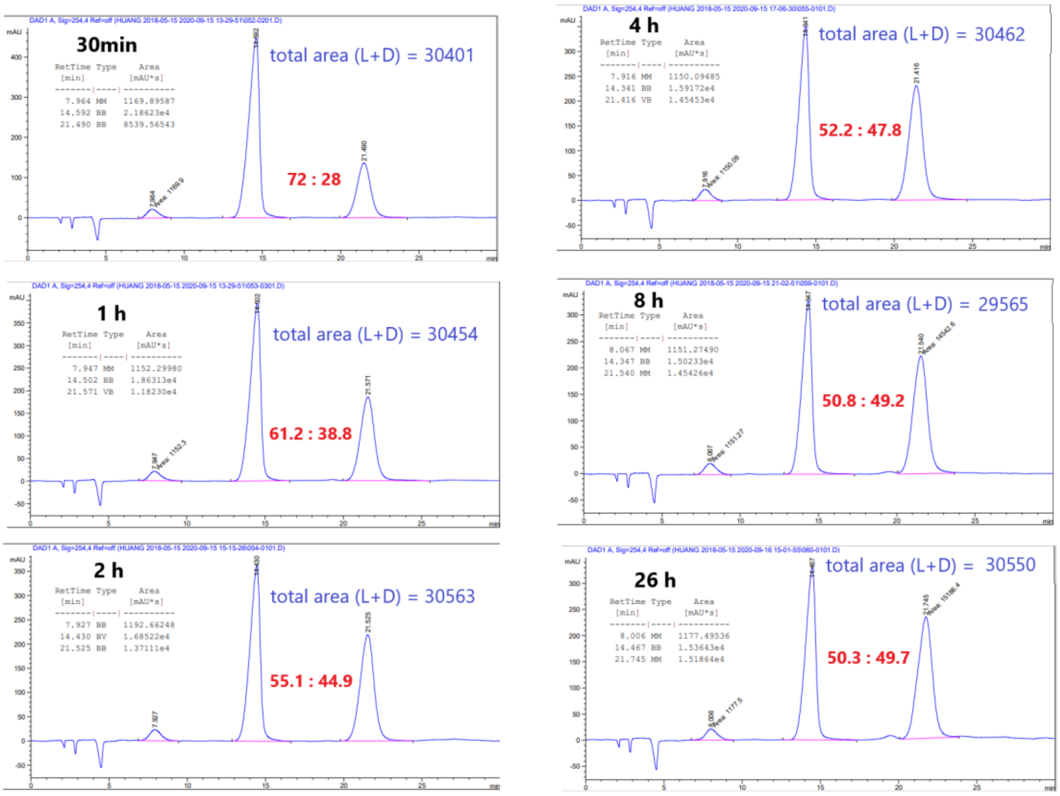

Supplementary Figure 21. HPLC kinetics of the catalytic racemization of L-phenylalanine (1.0 mol/L) in the presence of PLP (0.01 mol/L) and Cu^{2+} (0.01 mol/L), with 1.05 mol/L NaOH as base. HPLC samples were all taken same amount and diluted in same ratio. The combined integration area of both L- and D-Phe are constant, which indicate that there is not significant degradation of Phe during the racemization. The signal at 8.0 min is corresponding to ethylenediaminetetraacetate (EDTA), which was added as an integration reference.

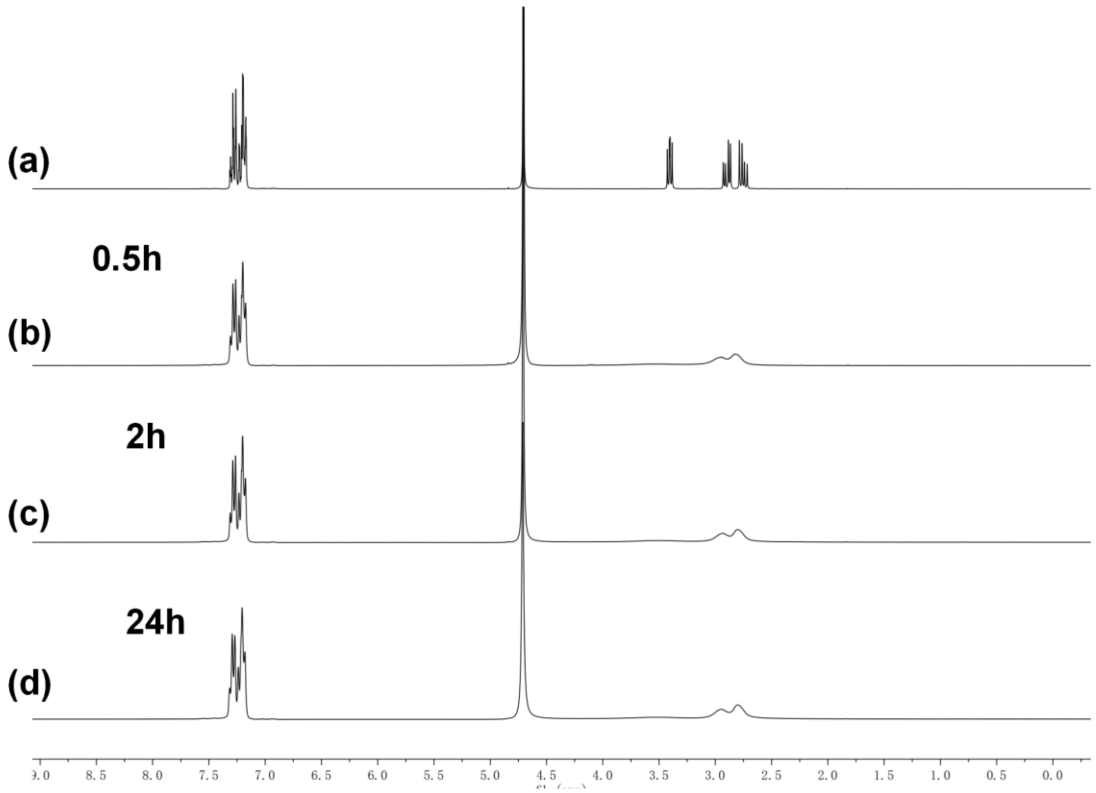

Supplementary Figure 22. ^{1}H NMR monitoring of the catalytic racemization mixture of D-Phe. (a) D-Phe sodium salt (pH 11-12) in D_2O. (b-f) D-Phe sodium salt (pH 11-12) in D_2O in the presence of PLP (1 mole%) and $CuSO_4$ (1 mole%) after 0.5, 2, 24h at room temperature, respectively.

Supplementary Table 2 summarizes racemization conditions for amino acids of the aqueous layer that were employed in the EECR experiments. Most racemizations and EECR experiments were conducted at room temperature (ca. 23˚C), with the exception of the bulkier amino acids valine (60˚C) and isoleucine (40˚C), which required moderately higher temperatures to achieve satisfactory racemization rates.

Supplementary Table 2. Racemization conditions and the efficiencies.[a]

Entry	Amino acid	NaOH (eq.)	PLP (eq.)	$CuSO_4$ (eq.)	Half lives[b] (min)
1	Phenylalanine	1.02	0.01	0.01	25
2	Alanine	1.02	0.01	0.01	20
3	Leucine	1.03	0.01	0.01	40
4	Isoleucine	1.05	0.02	0.02	53[c]
5	Methionine	1.03	0.01	0.005	35
6	Valine	1.05	0.02	0.02	70[d]
7	Tryptophan	1.02	0.01	0.005	28

[a] D-amino acids (4.0 mmol) in H_2O (2.0 mL) at room temp (ca. 23˚C); [b] Calculated from the HPLC data; [c] 40 ºC; [d] 60 ºC

6.2 Comparison of the transfer of Cu^{2+} from organic layer to aqueous layer in the EECR with (*R*)-1 and (*R*)-5 as extractors

As shown in the Supplementary Figure 23, when an EECR process was attempted with (*R*)-**1** as the extractant, the Cu^{2+} ions were transferred to organic layer, as evidenced by the change of the color of the organic layer from yellow to dark blue. The sequestration of the Cu ions in the organic layer was also found to shut down the catalysis of the racemization of the amino acid.

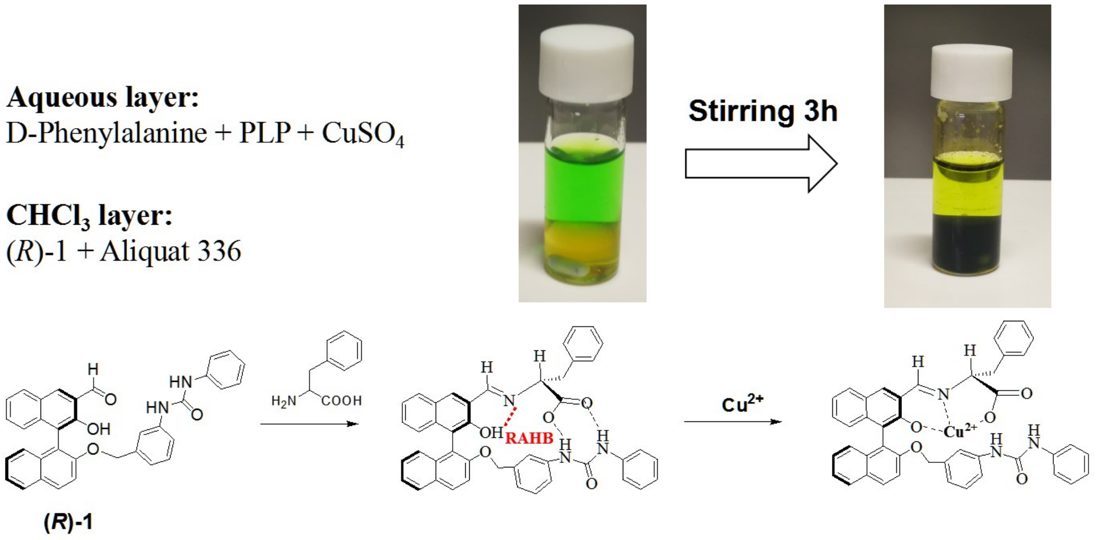

Supplementary Figure 23. Photographs of the EECR process using (*R*)-**1**, illustrating the sequestration of the Cu ions in the organic layer (top). Proposed rationale for the complexation of Cu ions by the coplanar, chelating ligand formed between (*R*)-**1** and the amino acid, responsible for the transfer of the Cu ions into the organic layer.

As shown in the Supplementary Figure 24, by contrast, when the EECR process was carried out with (*R*)-**5** as the extractant, no color change was perceived in the organic layer over the course of 5 EECR cycles. No leeching of Cu ions was detected by in the organic layer by atomic absorption spectroscopy ([Cu] ≤ 1 ppm). Furthermore, the racemization catalyst remained active in the aqueous layer.

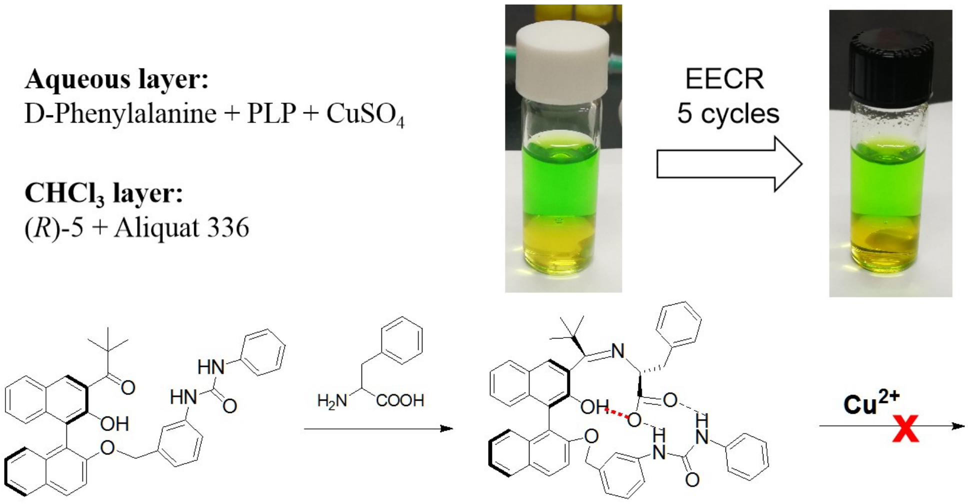

(*R*)-5

Supplementary Figure 24. Photographs of the EECR process using (*R*)-**5**, illustrating the lack of phase-transfer of the Cu ions from the aqueous to the organic layer (top). Proposed rationale for the absence of complexation of Cu ions by the non-coplanar, imine formed between (*R*)-**5** and the amino acid.

6.3 EECR Experiments

6.3.1 EECR of 1.1 equiv DL-Phe with (*R*)-5, single extraction.

The organic layer was prepared by dissolving (*R*)-**5** (0.60 g, 1.0 mmol) and 1.2 eq of Aliquat 336 in $CDCl_3$ (6 mL). The aqueous layer was prepared by dissolving DL-phenylalanine (0.18 g, 1.1 mmol), NaOH (0.048 g, 1.2 mmol), PLP (0.0027 g, 0.011 mmol) and $CuSO_4$ (0.0018 g, 0.011 mmol) in H_2O (1 mL). The two solutions were vigorously stirred in a 10-mL vial, and ^{1}H NMR spectra of the separated organic layer were acquired after 1h, 2h, 4h, 12h and 24h. The results are shown in Supplementary Figure 25. The extraction yield after 24h, as estimated by NMR was of 68% based on the extractor (*R*)-**5** and 62% based on the amino acid. The NMR study shows that the mismatched imine (*R*)-**5**-D-Phe is present as a minor constituent at the beginning of the EECR experiment, but later disappears. The qualitative kinetics for the formation and extraction of the imine (*R*)-**5**-L-Phe (and therefore (*S*)-**5**-D-Phe) may be estimated on the basis of these results.

(a)

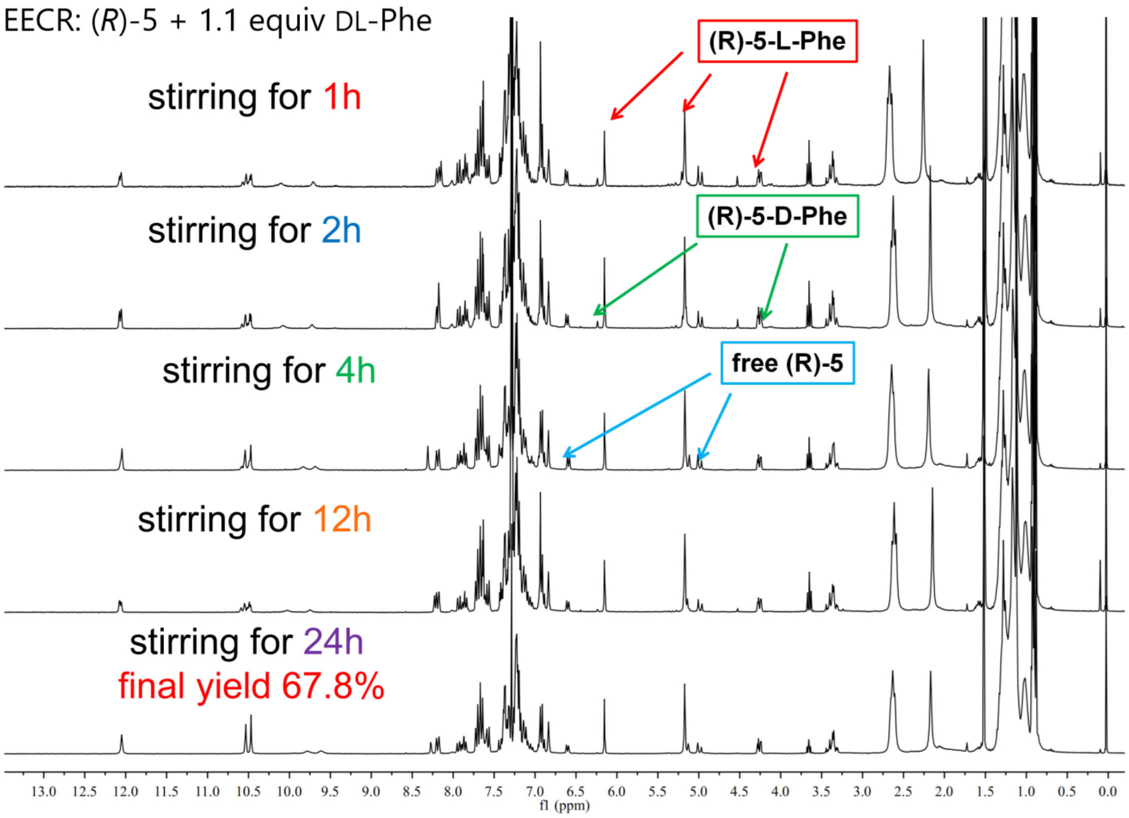

(b)

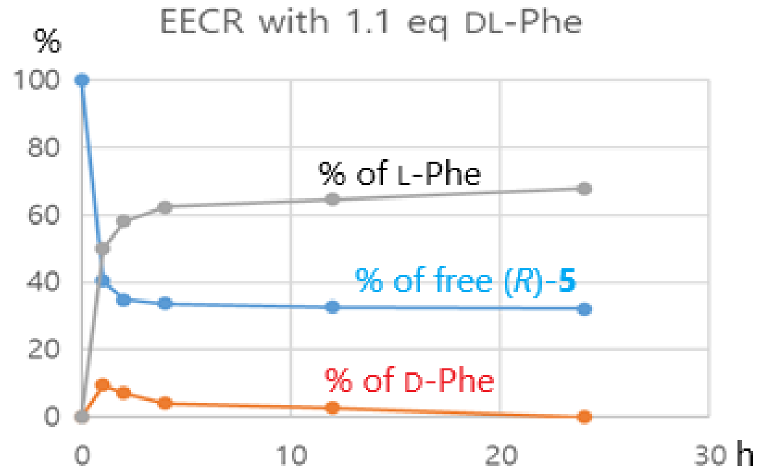

Supplementary Figure 25. (a) ^{1}H NMR monitoring of the separated organic layer in the EECR of DL-Phe (1.1 equiv) with (*R*)-**5** after 1, 2, 4, 12 and 24 h, respectively. (b) Evolution of the composition of the mixture in EECR as a function of time, as estimated by the ^{1}H NMR integration of the above spectra and referenced to the total amount of the extractant (*R*)-**5**.

6.3.2. EECR of 4 equiv D-Phe (4 equiv) with (*R*)-5, single extraction

The organic layer was prepared by dissolving (*R*)-**5** (0.90 g, 1.5 mmol) and 1.2 eq of Aliquat 336 in $CDCl_3$ (10 mL). The aqueous layer was prepared by dissolving D-phenylalanine (1.0 g, 6.0 mmol), NaOH (0.26 g, 6.6 mmol), PLP (0.016 g, 0.060 mmol) and $CuSO_4$ (0.010 g, 0.060 mmol) in H_2O (5 mL). The two solutions were vigorously stirred in a 20-mL vial, and ^1H NMR spectra of the separated organic layer were acquired after 0.5, 1h, 2h, 3h, 6h, and 24h. The results are shown in Supplementary Figure 26. The results indicate that imine formation is complete within 6h. The NMR study also shows that the mismatched imine (*R*)-**5**-D-Phe is present as a minor constituent at the beginning of the EECR experiment, but later disappears. On the basis of these results, the latter EECR experiments were carried out over ≥ 6h to maximize the selectivity of process.

(a)

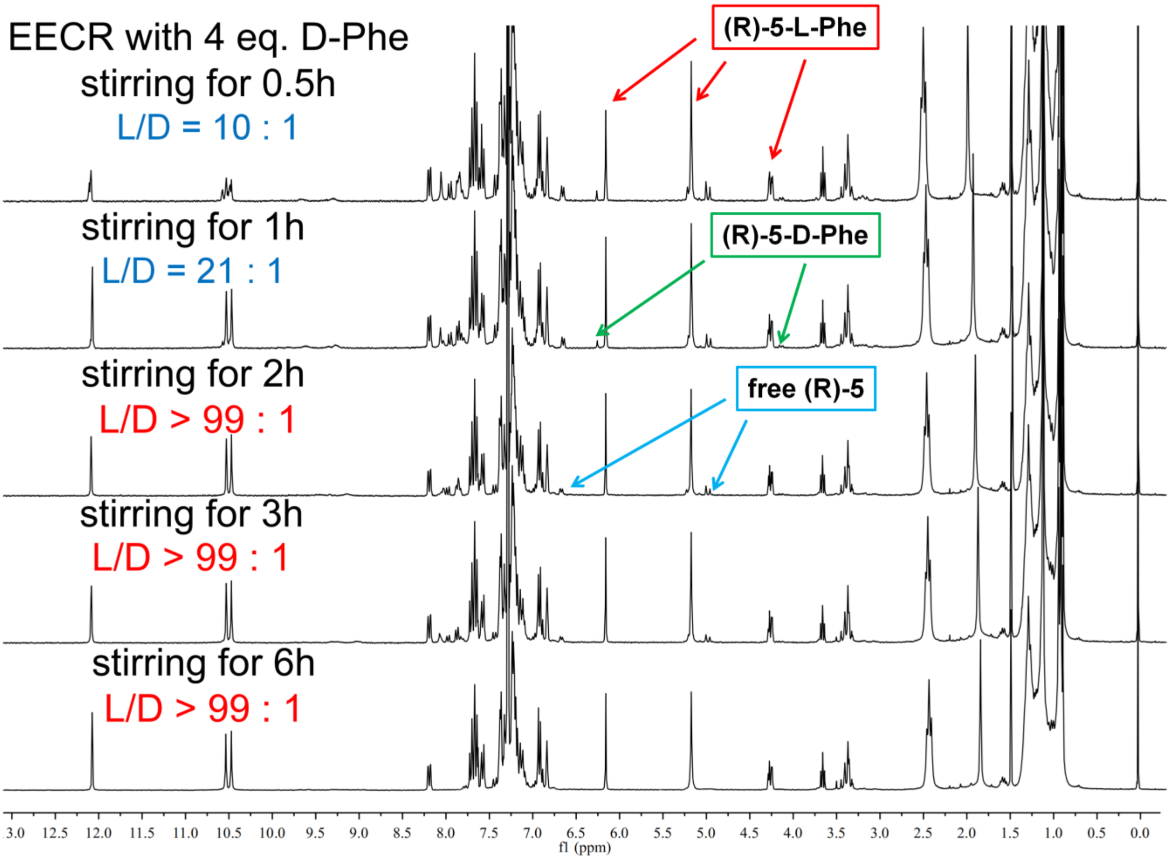

(b)

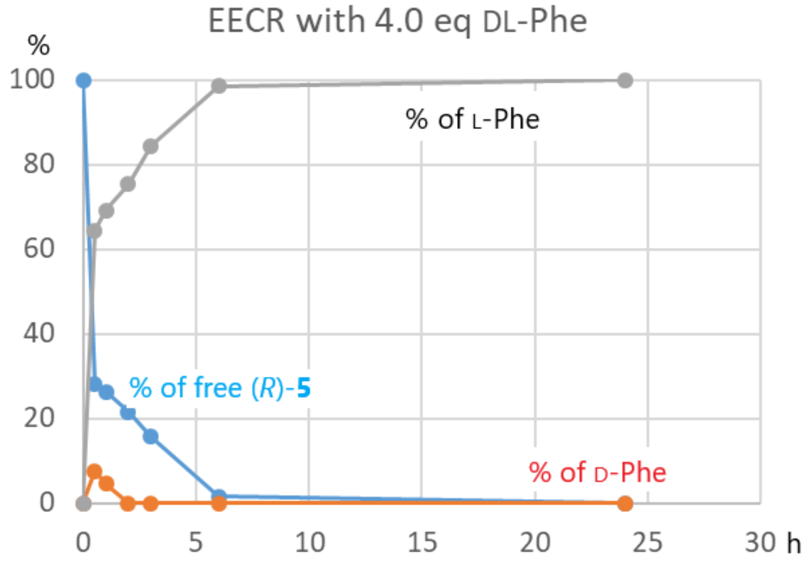

Supplementary Figure 26. (a) ^{1}H NMR monitoring of the separated organic layer in the EECR of D-Phe (4 equiv) with (*R*)-**5** after 0.5, 1, 2, 3, and 6 h, respectively. (b) Evolution of the composition of the mixture in EECR as a function of time, as estimated by the ^{1}H NMR integration of the above spectra, and referenced to the total amount of the extractant (*R*)-**5**.

6.3.3. EECR of 4 equiv DL-Phe with (*R*)-5, 4 repeated extractions and hydrolyses

Extraction (imine formation) Stage: The organic layer was prepared by dissolving (*R*)-**5** (0.90 g, 1.5 mmol) and 1.2 eq of Aliquat 336 in $CDCl_3$ (10 mL). The aqueous layer was prepared by dissolving DL-phenylalanine (1.0 g, 6.1 mmol), NaOH (0.27 g, 6.7 mmol), PLP (0.015 g, 0.061 mmol) and $CuSO_4$ (0.0097 g, 0.061 mmol) in H_2O (5 mL). The two solutions were vigorously stirred in a 20-mL vial for 5 h, and the ^1H NMR spectrum of the separated organic layer was acquired to confirm the stereoselective formation of the imine (*R*)-**5**-L-Phe.

Hydrolysis (back-extraction) Stage: The separated organic layer was then hydrolyzed by vigorously stirring in the presence of 2.0 *N* aqueous HCl (2.5 mL) for 4 h. After separation of the layers, the decanted organic layer containing (*R*)-**5** and Aliquat 336 was recombined with the previous aqueous layer containing the amino acid to carry out a second EECR cycle.

The two stages – extraction and hydrolysis – were repeated for a total of 4 cycles without further addition of DL-phenylalanine. Neutralization of the combined aqueous layer from the acidic hydrolysis with 2*N* NaOH induced precipitation of the amino acid, which was filtered, washed with cold water and ethanol, and finally dried to obtain L-Phe (0.72 g, 4.4 mmol) with an enantiopurity greater than 98% e.r. The isolated yield of L-Phe is 73% based on the amino acid, and 293% with respect to the extractant (*R*)-**5**.

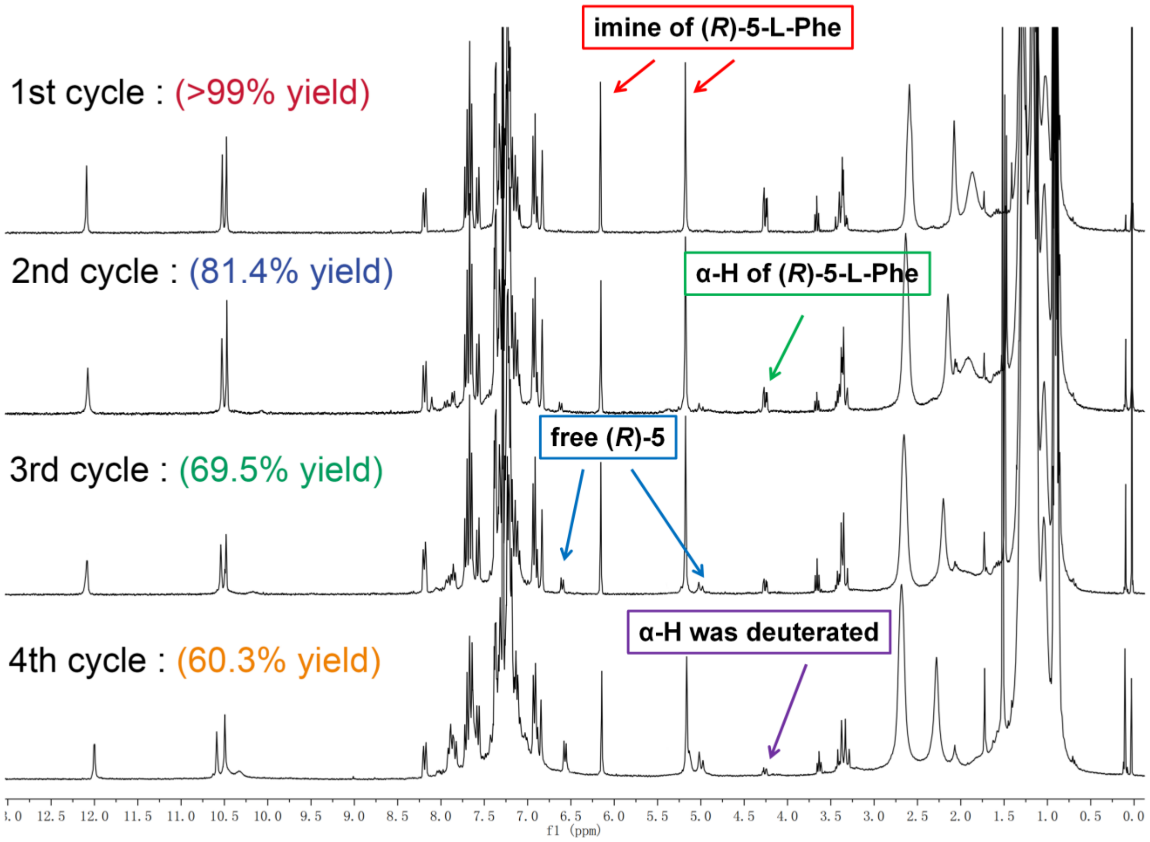

Supplementary Figure 27. ^1H NMR monitoring of the separated organic layer in the EECR of DL-Phe (4 equiv) with (*R*)-**5** (no DL-Phe added in cycles #2-4) upon each EECR cycle. Note that the NMR yield of the extracted imine (*R*)-**5**-L-Phe decreases after each cycle as the total [Phe] remaining in the aqueous layer decreases.

6.3.4 EECR of 4 equiv DL-Met (4 equiv) with (*R*)-5, 4 repeated extractions and hydrolyses

The procedure employed for the EECR experiment described in section 6.3.3 was repeated using DL-Met (0.91 g, 6.1 mmol) in place of DL-Phe. Over 4 extraction and hydrolysis cycles, L-Met (0.63 g, 4.2 mmol) with an enantiopurity greater than 98% e.r. was isolated, corresponding to a yield 70% based on the amino acid, and 280% with respect to the extractant (*R*)-**5**.

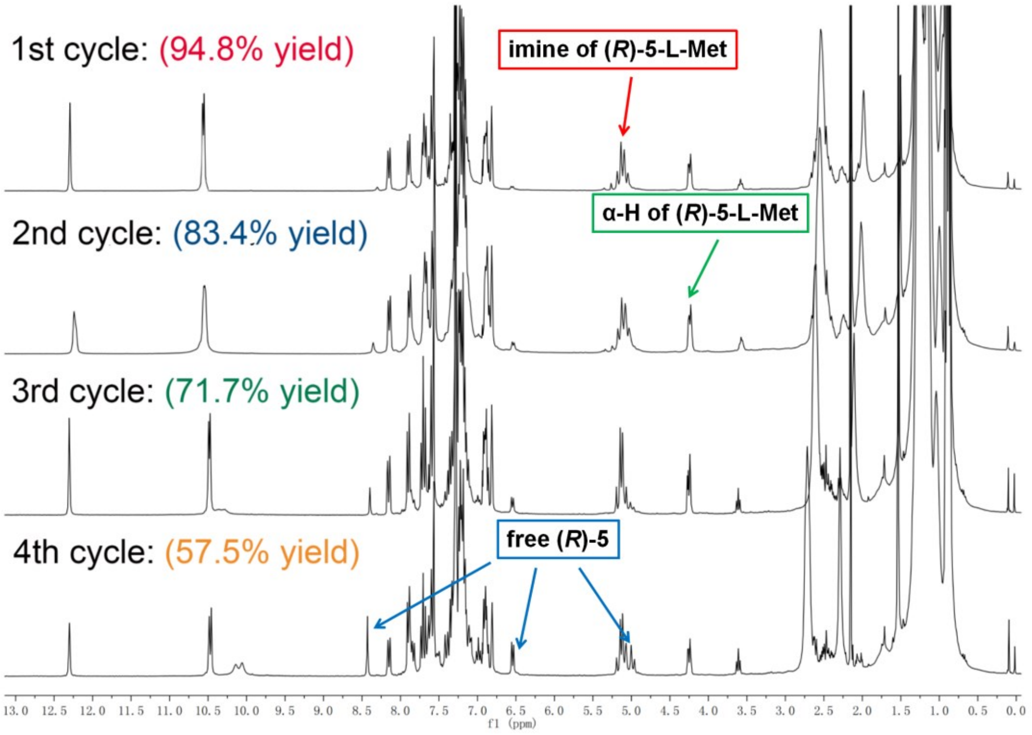

Supplementary Figure 28. ^{1}H NMR monitoring of the separated organic layer in the EECR of DL-Met (4 equiv) with (*R*)-**5** (no DL-Met added in cycles #2-4) upon each EECR cycle. Note that the NMR yield of the extracted imine (*R*)-**5**-L-Met decreases after each cycle as the total [Met] remaining in the aqueous layer decreases.

6.3.5 EECR of DL-naphthylalanine (Nal) (4 equiv) with (*R*)-5, 4 repeated extractions and hydrolyses

The procedure employed for the EECR experiment described in section 6.3.3 was repeated using DL-Nal (1.31 g, 6.1 mmol) in place of DL-Phe. Over 4 extraction and hydrolysis cycles, L-Nal (0.94 g, 4.2 mmol) with an enantiopurity greater than 98% e.r. was isolated, corresponding to a yield 73% based on the amino acid, and 293% with respect to the extractant (*R*)-**5**.

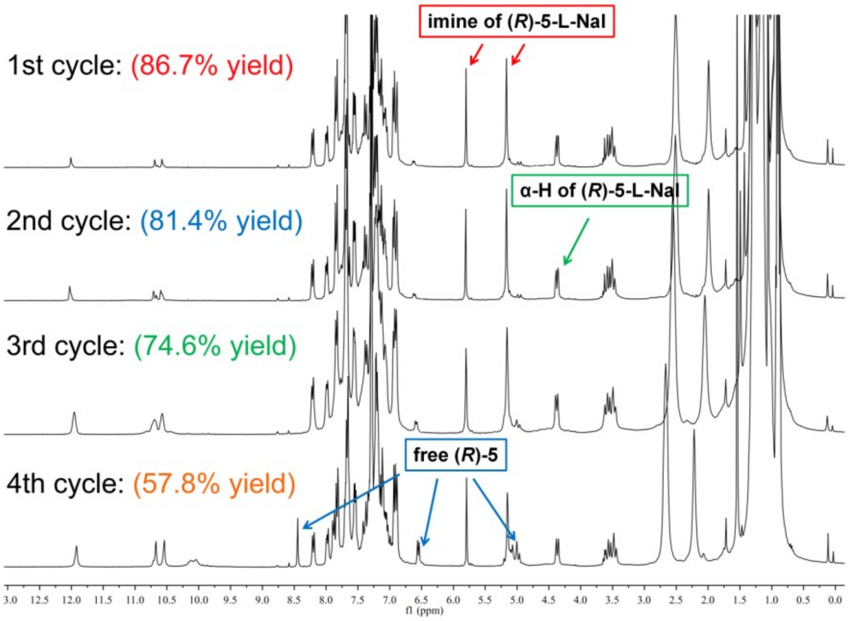

Supplementary Figure 29. ^{1}H NMR monitoring of the separated organic layer in the EECR of DL-Nal (4 equiv) with (*R*)-**5** (no DL-Nal added in cycles #2-4) upon each EECR cycle. Note that the NMR yield of the extracted imine (*R*)-**5**-L-Nal decreases after each cycle as the total [Nal] remaining in the aqueous layer decreases.

6.3.6 EECR of DL-isoleucine (Ile) (4 equiv) with (*R*)-5, 4 repeated extractions and hydrolyses

The procedure employed for the EECR experiment described in section 6.3.3 was repeated using DL-Ile in place of DL-Phe. Over 4 extraction and hydrolysis cycles, L-Ile (0.57 g, 4.4 mmol) with an enantiopurity greater than 98% e.r. was isolated, corresponding to a yield 72.1% based on the amino acid, and 288% with respect to the extractant (*R*)-**5**.

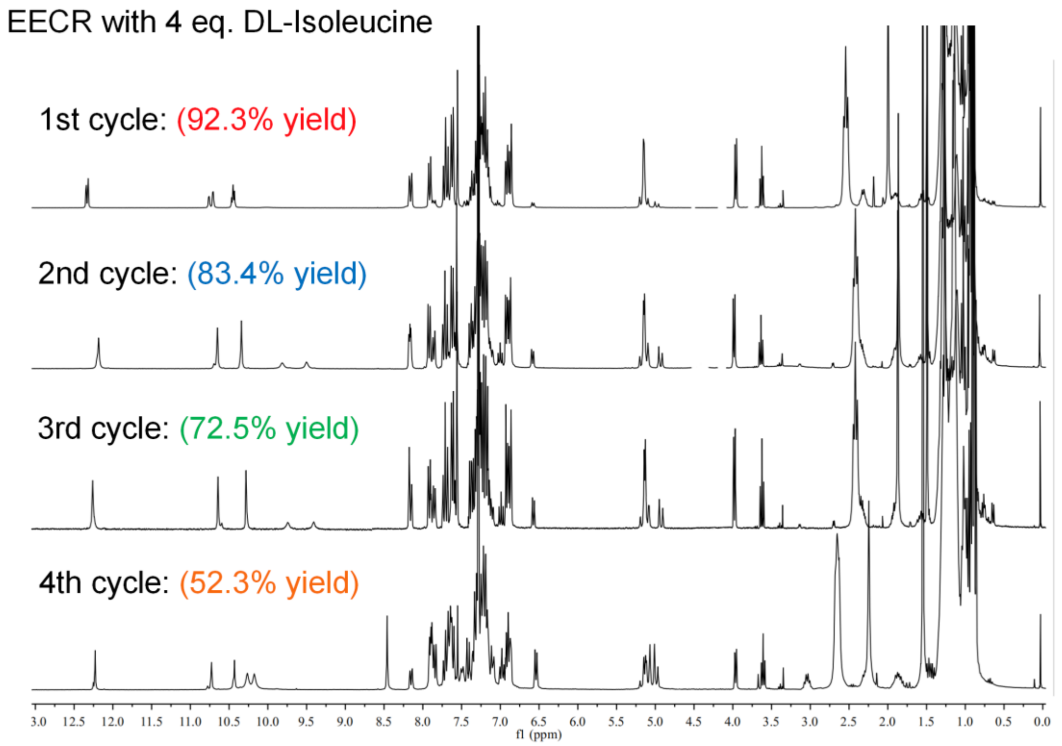

Supplementary Figure 30. ^{1}H NMR monitoring of the separated organic layer in the EECR of DL-Ile (4 equiv) with (*R*)-**5** upon each EECR cycle (no DL-Ile was added in cycles #2-4). Note that the NMR yield of the extracted imine (*R*)-**5**-L-Ile decreases after each cycle as the total [Ile] remaining in the aqueous layer decreases.

6.3.7 EECR of DL-leucine (Leu) (4 equiv) with (*R*)-5, 4 repeated extractions and hydrolyses

The procedure employed for the EECR experiment described in section 6.3.3 was repeated using DL-Leu in place of DL-Phe. Over 4 extraction and hydrolysis cycles, L-Leu (0.57 g, 4.4 mmol) with an enantiopurity greater than 98% e.r. was isolated, corresponding to a yield 72.1% based on the amino acid, and 288% with respect to the extractant (*R*)-**5**.

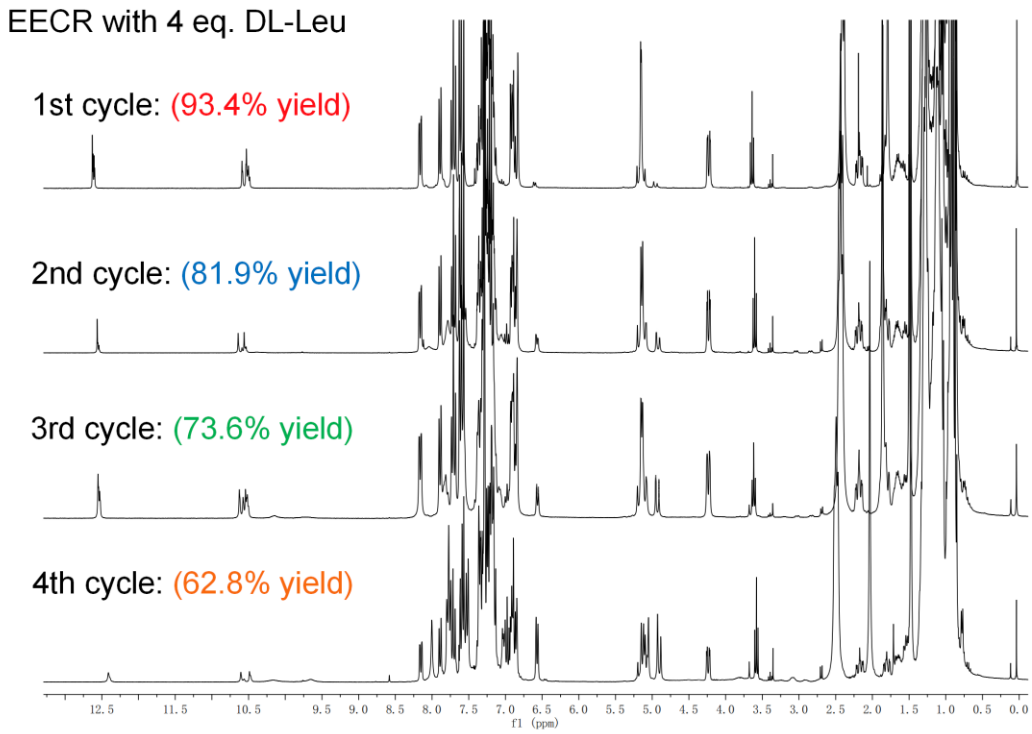

Supplementary Figure 31. ^{1}H NMR monitoring of the separated organic layer in the EECR of DL-Leu (4 equiv) with (*R*)-**5** upon each EECR cycle (no DL-Leu was added in cycles #2-4). Note that the NMR yield of the extracted imine (*R*)-**5**-L-Leu decreases after each cycle as the total [Leu] remaining in the aqueous layer decreases.

6.3.8 EECR of DL-valine (Val) (4 equiv) with (*R*)-5, 4 repeated extractions and hydrolyses

The procedure employed for the EECR experiment described in section 6.3.3 was repeated using DL-Val in place of DL-Phe. Over 4 extraction and hydrolysis cycles, L-Val (0.54 g, 4.6 mmol) with an enantiopurity greater than 98% e.r. was isolated, corresponding to a yield 75.1% based on the amino acid, and 300% with respect to the extractant (*R*)-**5**.

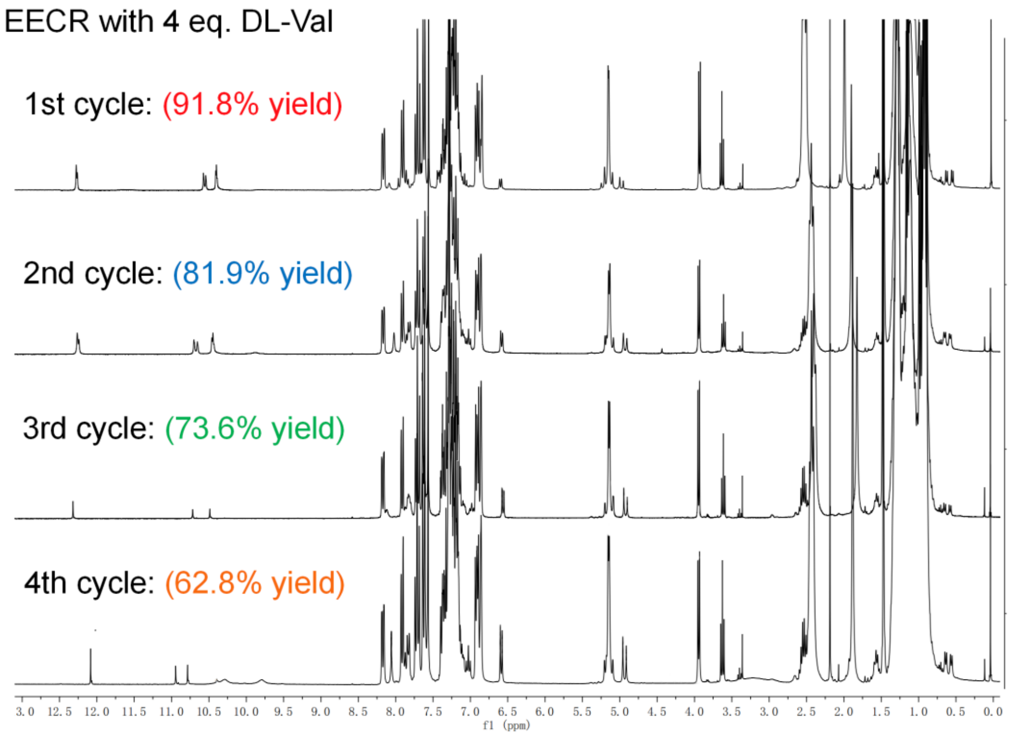

Supplementary Figure 32. ^{1}H NMR monitoring of the separated organic layer in the EECR of DL-Val (4 equiv) with (*R*)-**5** upon each EECR cycle (no DL-Val was added in cycles #2-4). Note that the NMR yield of the extracted imine (*R*)-**5**-L-Val decreases after each cycle as the total [Val] remaining in the aqueous layer decreases.

6.3.9 EECR of DL-tryptophan (Trp) (4 equiv) with (*R*)-5, 4 repeated extractions and hydrolyses

The procedure employed for the EECR experiment described in section 6.3.3 was repeated using DL-Trp in place of DL-Phe. Over 4 extraction and hydrolysis cycles, L-Trp (0.93 g, 4.5 mmol) with an enantiopurity greater than 98% e.r. was isolated, corresponding to a yield 74.5% based on the amino acid, and 298% with respect to the extractant (*R*)-**5**.

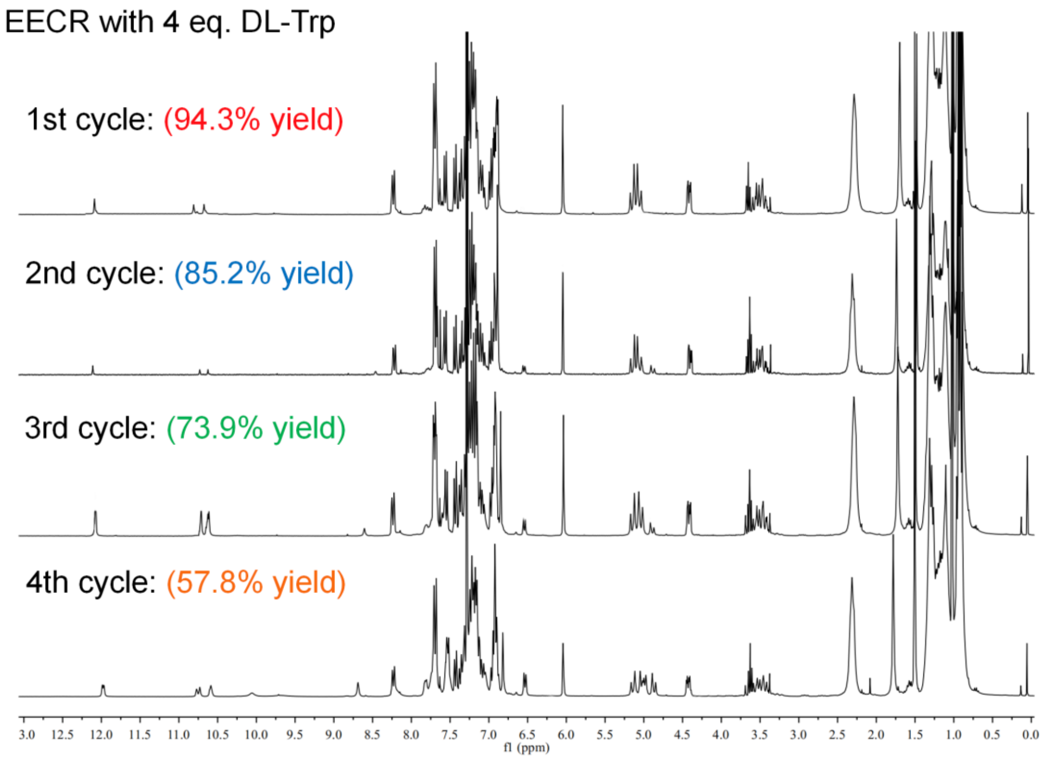

Supplementary Figure 33. ^{1}H NMR monitoring of the separated organic layer in the EECR of DL-Trp (4 equiv) with (*R*)-**5** upon each EECR cycle (no DL-Trp was added in cycles #2-4). Note that the NMR yield of the extracted imine (*R*)-**5**-L-Trp decreases after each cycle as the total [Trp] remaining in the aqueous layer decreases.

6.3.10 EECR of DL-Phe (4 equiv) with (*R*)-5 with toluene solvent, 4 repeated extractions and hydrolyses

The procedure employed for the EECR experiment described in section 6.3.3 was repeated for DL-Phe using toluene as the solvent in place of $CDCl_3$. Over 4 extraction and hydrolysis cycles, L-Phe (0.76 g, 4.6 mmol) with an enantiopurity greater than 98% e.r. was isolated, corresponding to a yield 75.3% based on the amino acid, and 301% with respect to the extractant (*R*)-**5**.

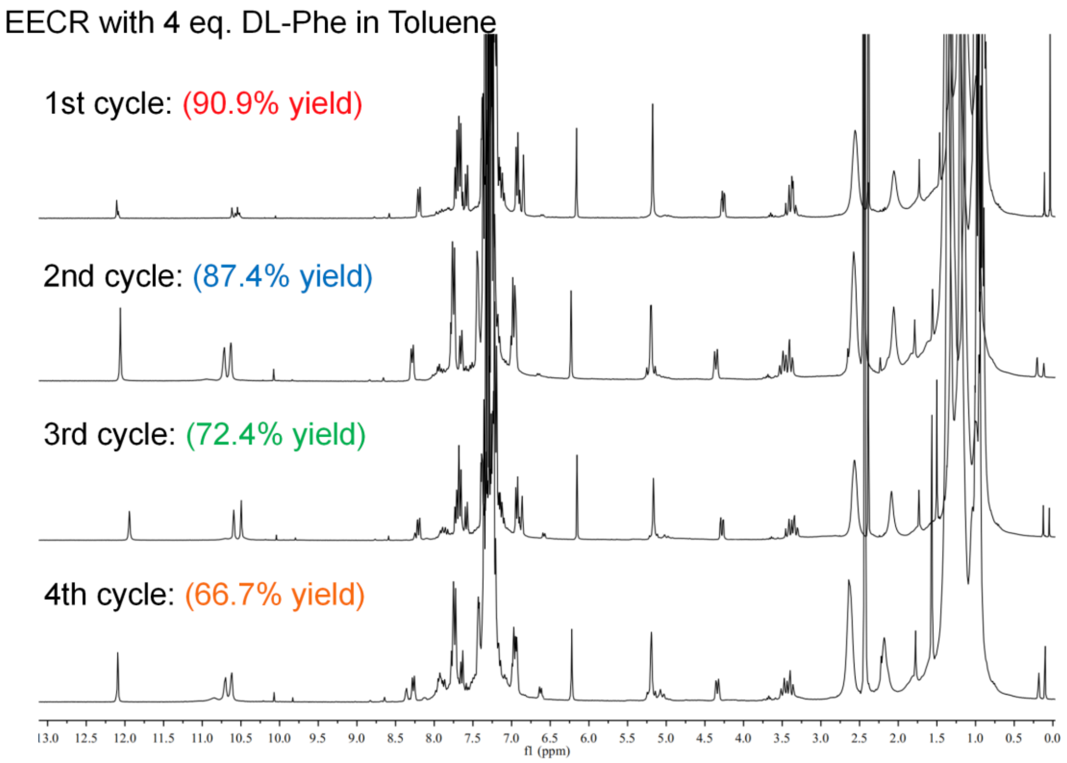

Supplementary Figure 34. ^{1}H NMR monitoring of the separated organic layer in the EECR of DL-Phe (4 equiv) with (*R*)-**5** upon each EECR cycle (no DL-Phe was added in cycles #2-4). The organic solvent toluene was used. Note that the NMR yield of the extracted imine (*R*)-**5**-L-Phe decreases after each cycle as the total [Phe] remaining in the aqueous layer decreases.

6.3.11 EECR of DL-Phe (4 equiv) with (*R*)-5 using *tert*-butyl methyl ether (MTBE) as the solvent, 4 repeated extractions and hydrolyses

The procedure employed for the EECR experiment described in section 6.3.3 was repeated for DL-Phe using *tert*-butyl methyl ether as the solvent in place of $CDCl_3$. Over 4 extraction and hydrolysis cycles, L-Phe (0.77 g, 4.7 mmol) with an enantiopurity greater than 98% e.r. was isolated, corresponding to a yield 76.3% based on the amino acid, and 305% with respect to the extractant (*R*)-**5**.

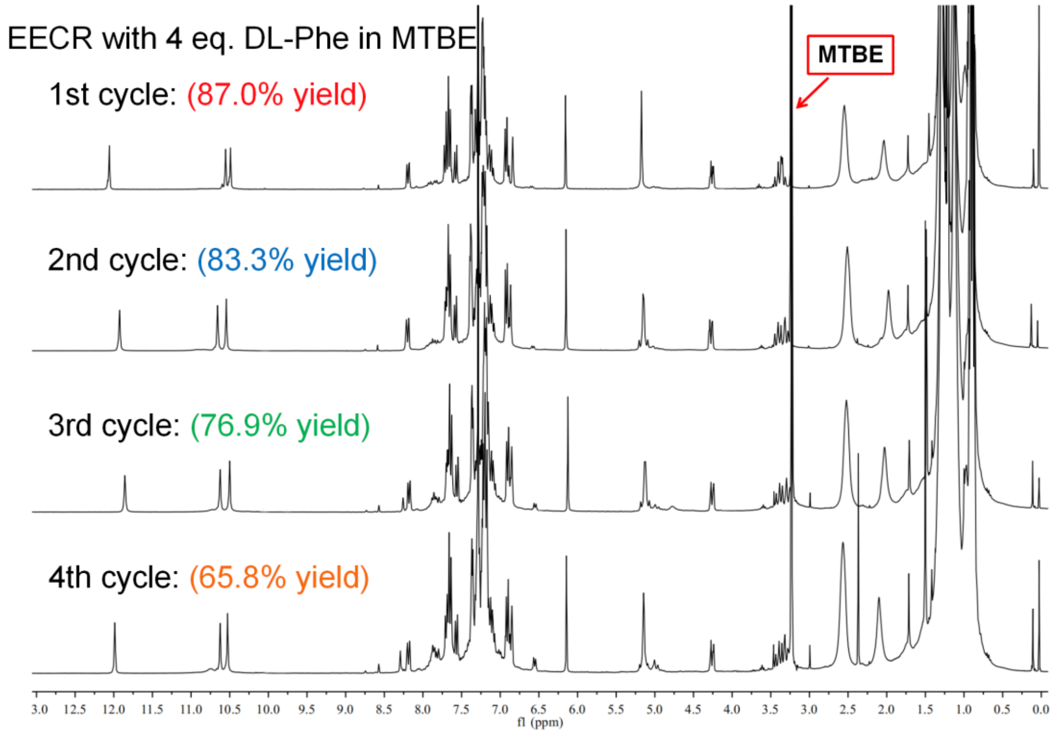

Supplementary Figure 35. ^{1}H NMR monitoring of the separated organic layer in the EECR of DL-Phe (4 equiv) with (*R*)-**5** upon each EECR cycle (no DL-Phe was added in cycles #2-4). The organic solvent MTBE was used. Note that the NMR yield of the extracted imine (*R*)-**5**-L-Phe decreases after each cycle as the total [Phe] remaining in the aqueous layer decreases.

6.3.12 EECR of DL-Phe (4 equiv) with (*R*)-5 with 2-methyltetrahydrofuran (MeTHF) solvent, 4 repeated extractions and hydrolyses

The procedure employed for the EECR experiment described in section 6.3.3 was repeated for DL-Phe using 2-methyltetrahydrofuran as the solvent in place of $CDCl_3$. Over 4 extraction and hydrolysis cycles, L-Phe (0.74 g, 4.5 mmol) with an enantiopurity greater than 98% e.r. was isolated, corresponding to a yield 73.2% based on the amino acid, and 293% with respect to the extractant (*R*)-**5**.

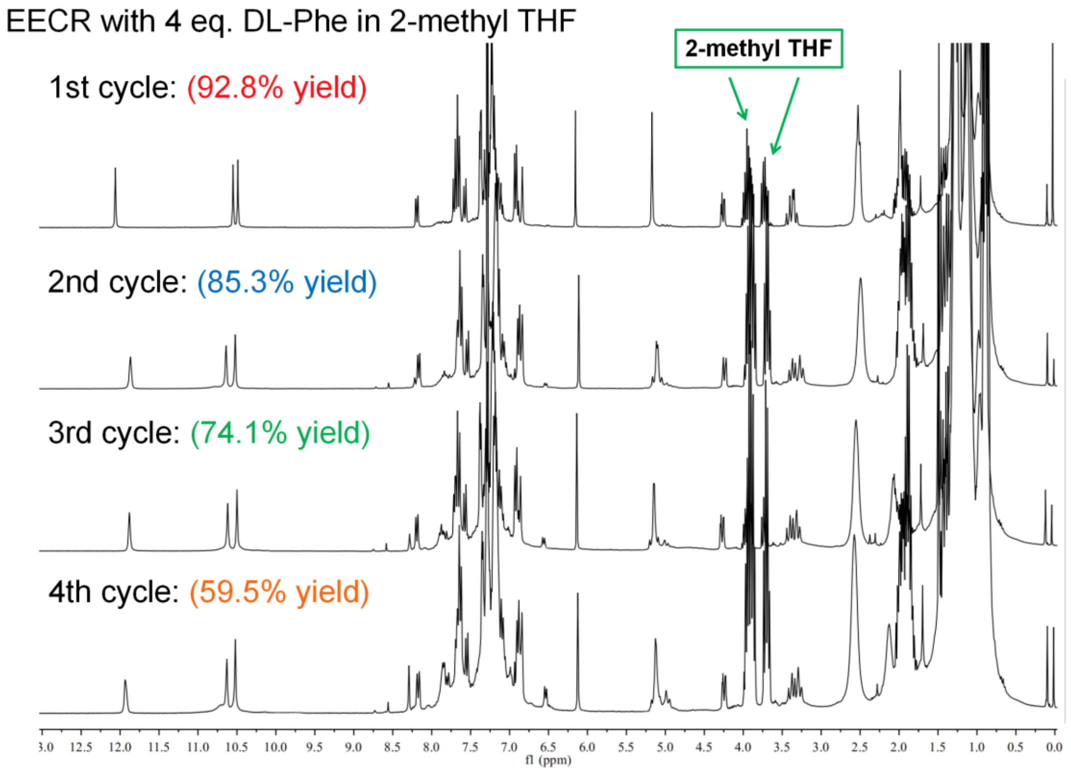

Supplementary Figure 36. ^{1}H NMR monitoring of the separated organic layer in the EECR of DL-Phe (4 equiv) with (*R*)-**5** upon each EECR cycle (no DL-Phe was added in cycles #2-4). The organic solvent MeTHF was used. Note that the NMR yield of the extracted imine (*R*)-**5**-L-Phe decreases after each cycle as the total [Phe] remaining in the aqueous layer decreases.

6.3.13 EECR of D-Phe (4+4 equiv) with (*R*)-5, five cycles of EECR

This experiment is similar to that described in section 6.3.3, with the difference that one equivalent of Phe was added from the second cycle to the fifth cycles of EECR. In total 5 cycles of EECR were carried out usingD-Phe; thus, a total of 8 equivalents of D-Phe were added. This experiment shows the conversion of D-Phe to L-Phe (**Supplementary Figure 38**).

Extraction (imine formation) Stage: The organic layer was prepared by dissolving (*R*)-**5** (0.60 g, 1.0 mmol) and 1.05 eq of Aliquat 336 (0.49 g, 1.05 mmol) in $CDCl_3$ (1.0 mL). The aqueous layer was prepared by dissolving D-phenylalanine (0.66 g, 4.0 mmol), NaOH (0.18 g, 4.4 mmol), PLP (0.011 g, 0.040 mmol) and $CuSO_4$ (0.0064 g, 0.040 mmol) in H_2O (2 mL). The two solutions were vigorously stirred in a 5-mL vial at room temperature (20-25˚C) for 6 h, until complete formation of the imine (*R*)-**5**-L-Phe (≥90% vs. (*R*)-**5** by ^{1}H-NMR in the separated organic layer) (**Supplementary Figure 38-a**).

Hydrolysis (back-extraction) Stage: The separated organic layer was then hydrolyzed by vigorously stirring in the presence of 2.0 *N* aqueous HCl (2.0 mL) for 2 h at room temperature. After separation of the layers, the decanted organic layer containing the cleanly recovered (*R*)-**5** and Aliquat 336 was recombined with the previous aqueous layer containing the amino acid to carry out a second EECR cycle.

The two stages – extraction and hydrolysis – were repeated for a total of 5 cycles with the addition of one equivalent of the sodium salt of the amino acid [e.g. D-Phe (0.165 g, 1.0 mmol) and NaOH (0.04 g, 1.0 mmol)] to replenish the aqueous layer after each cycle. Neutralization of the combined aqueous layer from the acidic hydrolysis with 2*N* NaOH induced precipitation of the amino acid, which was filtered, washed with cold water and ethanol, and finally dried to obtain L-Phe (0.68 g, 4.1 mmol) with an enantiopurity greater than 98% e.r. The isolated yield of L-Phe is 51% based on the total amino acid, and 410% with respect to the extractant (*R*)-**5**.

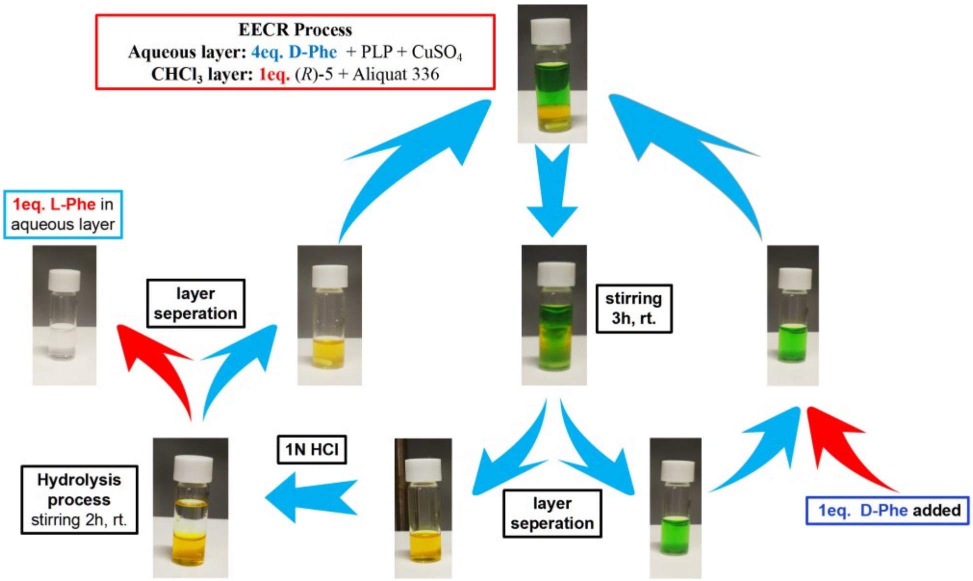

Supplementary Figure 37. Schematic representation of EECR for the stereoconversion of D-Phe to L-Phe with (*R*)-**5**.

(a)

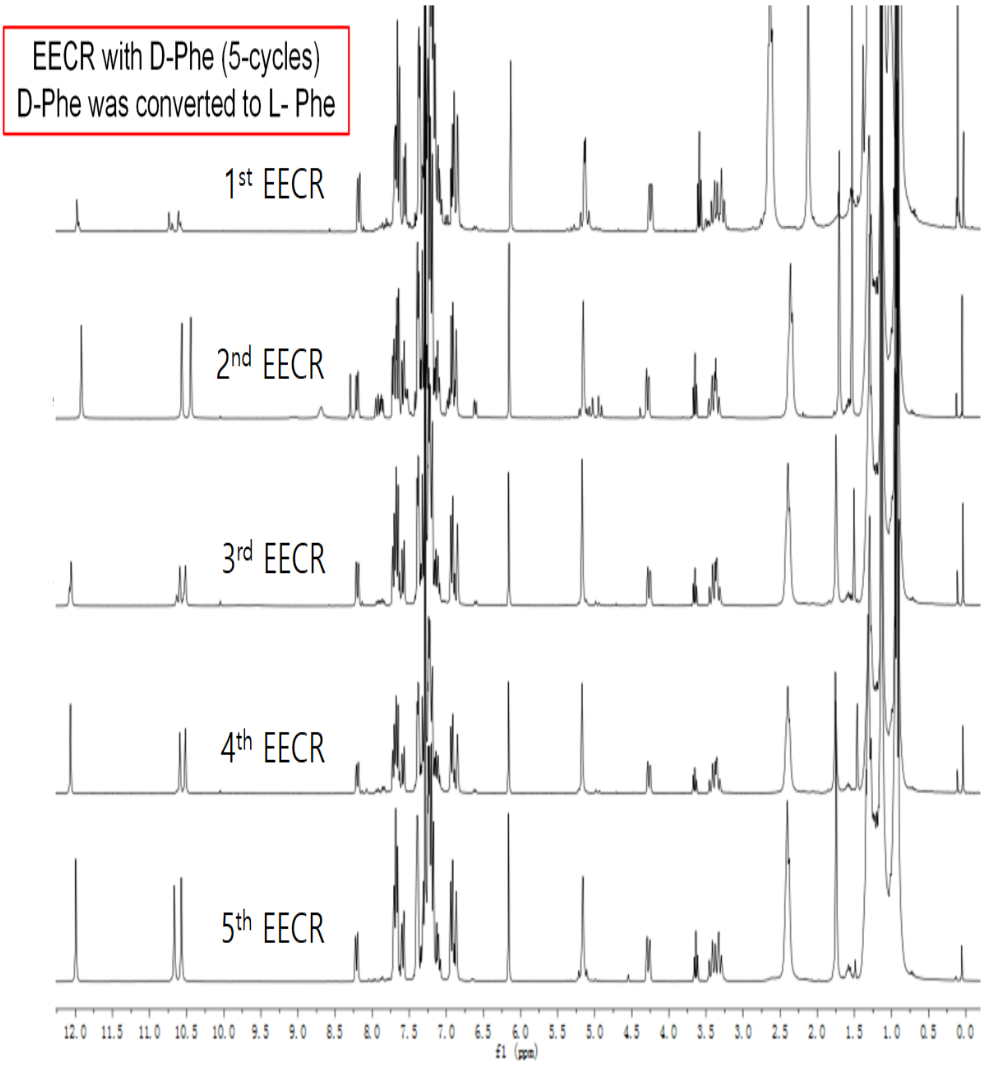

Supplementary Figure 38-(a). ^1H NMR monitoring of the separated organic layer in the EECR of D-Phe (4+4 equiv) with (*R*)-**5** upon each EECR cycle (1 equiv. D-Phe was added in cycles #2-5). Note that the NMR shows exclusive selectivity for the extraction of the (*R*)-**5**-L-Phe imine.

(b)

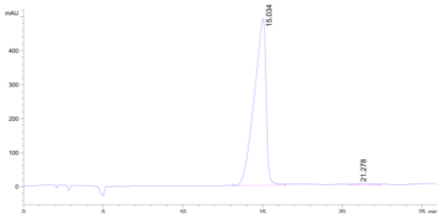

Signal 1: DAD1 A, Sig=254,4 Ref=off

Peak #	RetTime [min]	Type	Width [min]	Area [mAU*s]	Height [mAU]	Area %
1	15.034	VV	0.7417	2.53324e4	491.15659	99.0199
2	21.278	VV	0.8539	250.74321	3.83967	0.9801

Obtained Phenylalanine after 1st cycle of EECR:
L / D = 99.0 / 1.0

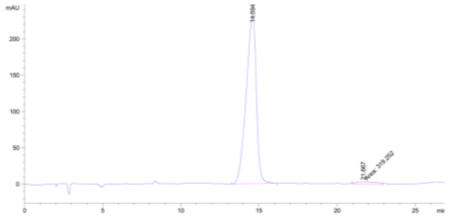

Signal 1: DAD1 A, Sig=254,4 Ref=off

Peak #	RetTime [min]	Type	Width [min]	Area [mAU*s]	Height [mAU]	Area %
1	14.594	BB	0.6808	1.04800e4	234.75475	97.0437
2	21.667	MM	1.6115	319.25214	3.30172	2.9563

Obtained Phenylalanine after 2nd cycle of EECR:
L / D = 97.1 / 2.9

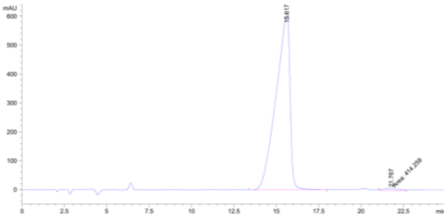

Signal 1: DAD1 A, Sig=254,4 Ref=off

Peak #	RetTime [min]	Type	Width [min]	Area [mAU*s]	Height [mAU]	Area %
1	15.617	BB	0.8154	3.49022e4	610.10028	98.8270
2	21.767	MM	1.2102	414.25882	5.70507	1.1730

Obtained Phenylalanine after 3rd cycle of EECR:
L / D = 98.8 / 1.2

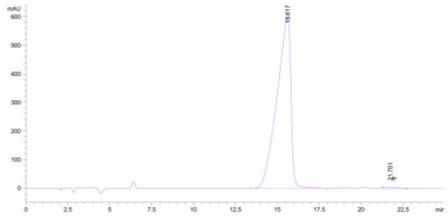

Signal 1: DAD1 A, Sig=254,4 Ref=off

Peak #	RetTime [min]	Type	Width [min]	Area [mAU*s]	Height [mAU]	Area %
1	15.617	BB	0.8154	3.49022e4	610.10028	99.1802
2	21.701	MM	0.6832	288.50110	5.66663	0.8198

Obtained Phenylalanine after 4th cycle of EECR:
L / D = 99.2 / 0.8

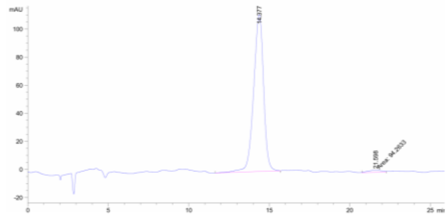

Signal 1: DAD1 A, Sig=254,4 Ref=off

Peak #	RetTime [min]	Type	Width [min]	Area [mAU*s]	Height [mAU]	Area %
1	14.377	BV	0.6621	4803.53125	111.61678	98.0754
2	21.598	MM	0.7677	94.26327	1.46878	1.9246

Obtained Phenylalanine after 5th cycle of EECR:
L / D = 98.1 / 1.9

Supplementary Figure 38-(b). HPLC chromatograms of the Phe obtained upon each of the 5 repeated cycles of EECR of D-Phe with (*R*)-**5** (1 equiv. D-Phe was added in cycles #2-5). The peak at ~14.5 min correspond to D-Phe and the peak at ~22.0 min to L-Phe. Average Enantiomeric ratio (L:D) over 5 EECR cycles = 98.3 : 1.7

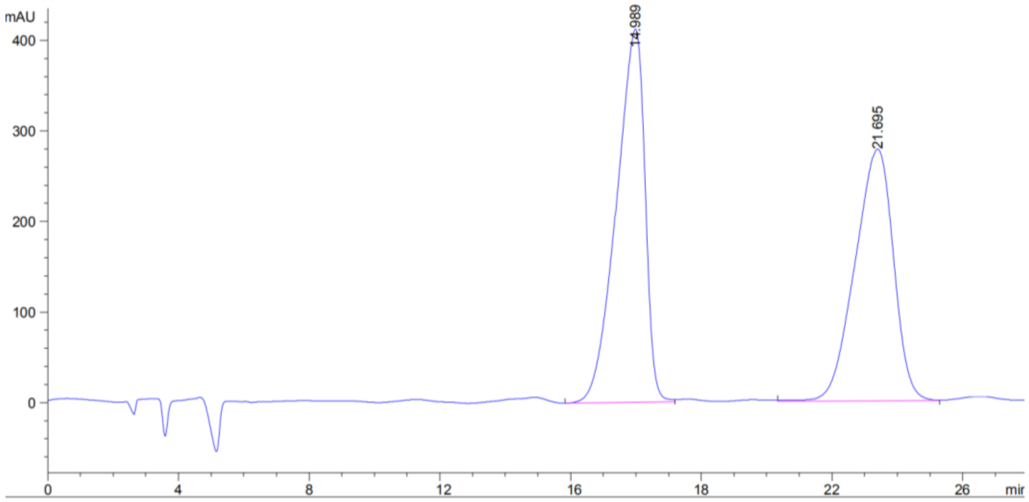

Signal 1: DAD1 A, Sig=250,4 Ref=off

Peak #	RetTime [min]	Type	Width [min]	Area [mAU*s]	Height [mAU]	Area %
1	14.989	BV	0.4686	1.25917e4	412.00699	52.5630
2	21.695	VV	0.6213	1.13638e4	277.78162	47.4370

Supplementary Figure 38-(c). HPLC chromatogram of the residual aqueous layer after 5 cycles of extraction and hydrolysis in the EECR of D-Phe with (*R*)-**5** (1 equiv D-Phe was added in cycles #2-5), indicating that the racemization catalyst remains active over the duration of the experiment.

6.3.14 EECR of other amino acids showing the conversion of D-AAs to L-AAs

The procedure employed for EECR experiment described in section **6.3.13** was repeated using D-Ala, D-Leu, D-Ile, D-Val, D-Met and D-Trp, respectively, in place of D-Phe. The ^{1}H NMR for the organic layer after first cycle of EECR are shown in **Supplementary Figure 39**. These results demonstrate that D-AAs are successfully converted to L-AAs during the EECR experiment.

Supplementary Table 3 lists the imine formation yields and enantioselectivities assessed from the ^{1}H NMR spectra shown in **Supplementary Figure 38** and **39**.

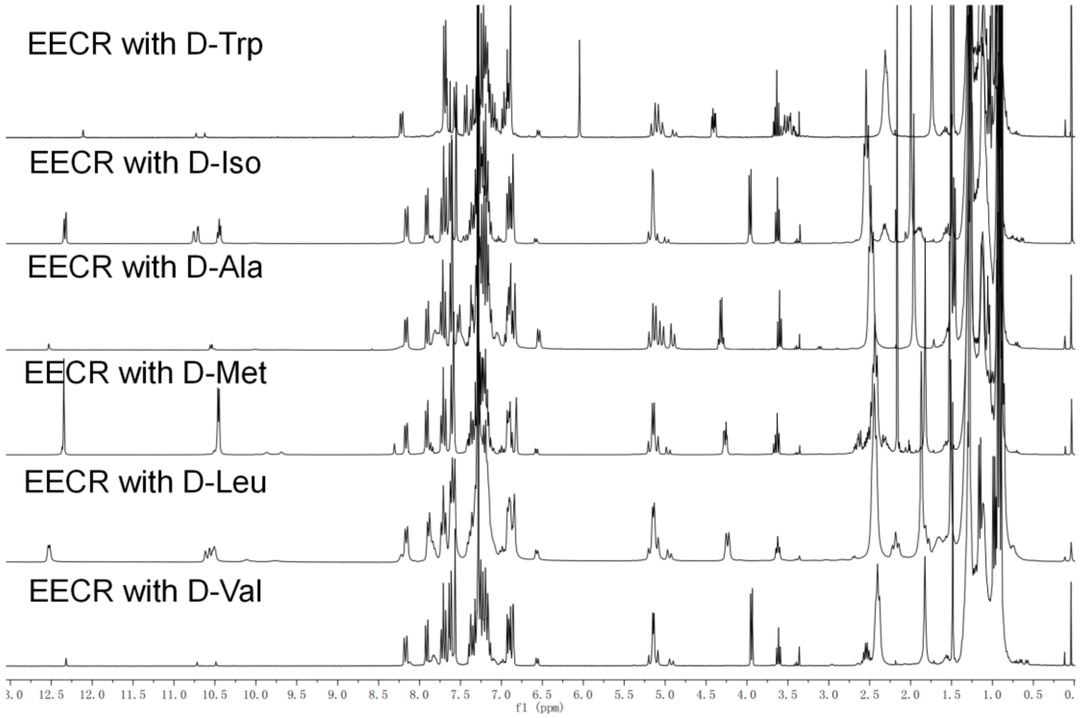

Supplementary Figure 39. ^{1}H NMR monitoring of the separated organic layers following the EECR of D-AAs (4 equiv) with (*R*)-**5**. The ^{1}H NMR spectra correspond to those of (*R*)-**5**-L-AA, which demonstrate that D-AAs were converted to L-AA.

Supplementary Table 3. The imine formation yields and stereoselectivities in the EECR of D-AAs assessed from the analyses of the ^{1}H NMR spectra in Supplementary Figures 38 and 39.

Amino Acids introduced	(*R*)-**5**-L-AA Imine formation NMR Yield	(*R*)-**5**-L-AA Imine Stereoselectivity
D-Phe	95%	>99/1
L-Phe	95%	<1/99
D-Ala	67%	>99/1
D-Leu	85%	>99/1
D-Ile	86%	>99/1
D-Val	80%	>99/1
D-Trp	87%	>99/1
D-Met	84%	>99/1

EECR conditions: (*R*)-**5** (1.0 mmol), Aliquat 336 (1.05 mmol) in $CDCl_3$ (1 mL); Amino acid (4.0 mmol), NaOH (4.05~4.2 mmol), PLP (0.04 mmol), $CuSO_4$ (0.04 mmol) in water (3 mL).

7. Continuous EECR of L-Phe with (*S*)-5.

The stepwise EECR approach described in Supplementary Sections 6.4 and 6.5 requires tedious manual work and induces losses during each operation. To address these issues was designed the continuous reactor shown in Figure 4 of the manuscript.

7.1 Experimental conditions for the operation of the continuous reactor:

Preparation of EECR solutions

Extraction (EECR) stage: The aqueous layer was prepared by dissolving L-Phe (5.0 g, 30 mmol), NaOH (1.32 g, 33 mmol, PLP (0.074 g, 0.3 mmol) and $CuSO_4$ (0.048 g, 0.3 mmol) in water (20 mL, final pH = 12.0). The organic layer was prepared by dissolving (*S*)-**5** (4.5 g, 7.6 mmol) and Aliquat 336 (3.4 g, 8.5 mmol) in CH_2Cl_2 (40 mL).

Hydrolysis stage: 2.0 *M* aqueous HCl solution was used in the aqueous layer.

Residual acid scavenging stage: 1.0 *M* Na_2CO_3 aqueous solution was used to remove residual traces of HCl in the CH_2Cl_2 layer.

Transfer rate of pumps 1-5: 0.15 mL/min

Continuously recycling EECR reactor operation

After continuous operation of the reactor for 5h, one additional equivalent of L-phenylalanine (1.25 g, 7.6 mmol) and NaOH (0.3 g, 7.6 mmol) were added to extraction (EECR) stage. The addition was repeated again after 5h additional operation. The reactor was finally operated continuously for 38 h more without further addition of L-Phe/NaOH. At the end of the run, the collected aqueous layer in the hydrolysis stage was neutralized with dilute aq. NaOH, and the precipitated amino acid was washed with cold water, ethanol and dried to yield D-Phe (4.9 g, 29.7 mmol) with an enantiopurity >98%. The total yield was 390% based on (*S*)-**5**, and 65% based on the phenylalanine used.

7.2 Extraction efficiency and stereoselectivity during the continuous EECR.

Aliquots of the organic layer within the continuous EECR reactor were taken at regular intervals and analyzed by ^{1}H-NMR to assess the efficiency and selectivity of the formation of the (*S*)-**5**-D-Phe imine (**Supplementary Figure 40**). Extraction yields were found to be maintained in the 90-95% range with respect to the total (*S*)-**5**, and the stereoselectivity was found to be exclusive (>99:1 D:L). These results further confirm the efficient racemization of Phe in the aqueous layer.

No significant changes in the extraction yields or stereoselectivity were observed over the ca. 40h run, suggesting that degradation of the amino acids, extractant or racemization catalyst must be negligible over this timescale. Moreover, ^{1}H-NMR analysis of the organic layer of the hydrolysis stage indicates that the hydrolysis of the imine is fast and complete under those conditions, and that the recycled (*S*)-**5** does not show signs of degradation over the ca. 40h run (**Supplementary Figure 41**).

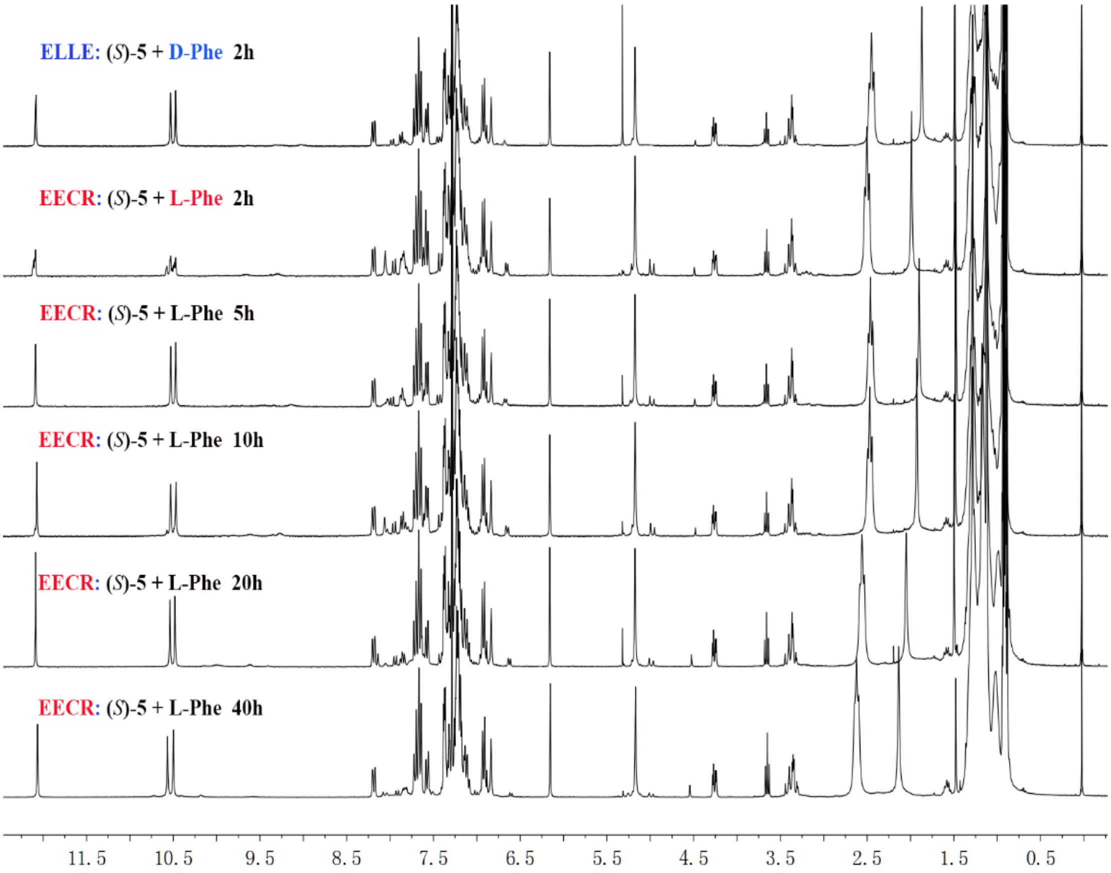

Supplementary Figure 40. Time dependent ^{1}H NMR of the separated organic layer in the extraction (EECR) stage of the continuous reactor during the continuous EECR of L-Phe with (*S*)-**5** over 40 h. The top spectrum is that of the (*S*)-**5**-D-Phe imine obtained in a separate ELLE experiment for reference.

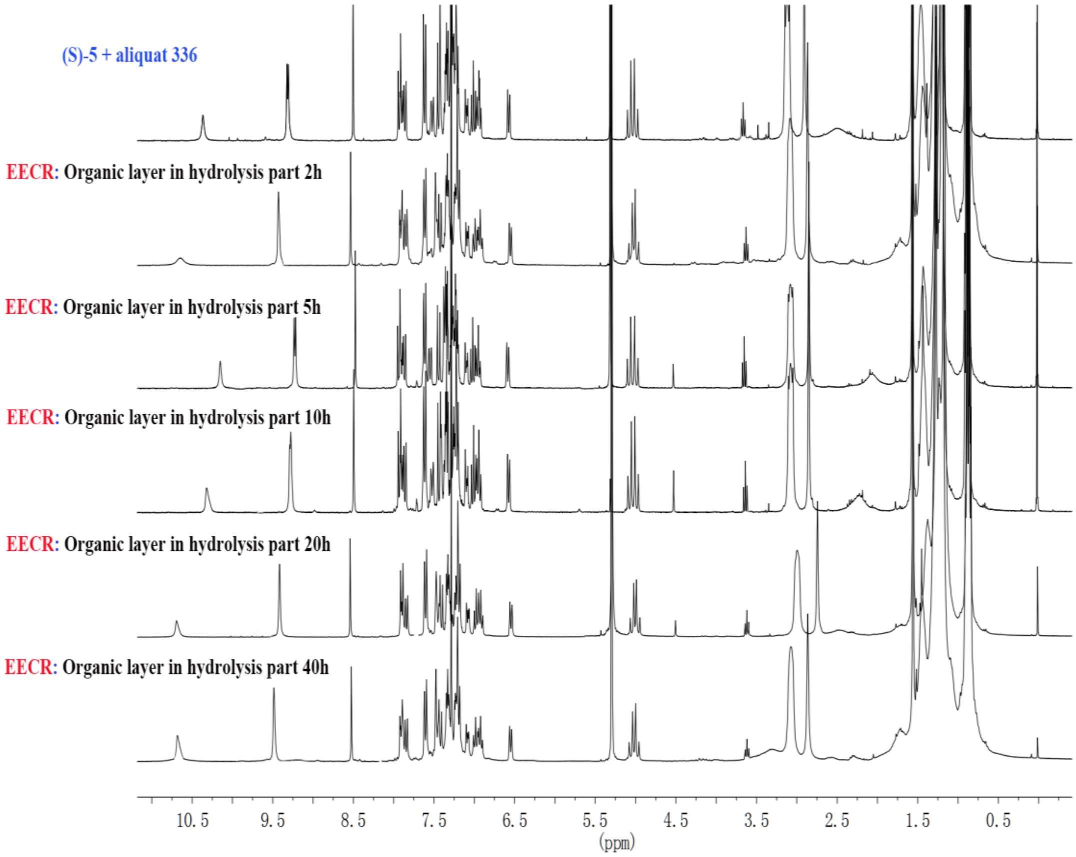

Supplementary Figure 41. Time dependent ^{1}H NMR of the separated organic layer in the hydrolysis (EECR) stage of the continuous reactor during the continuous EECR of L-Phe with (*S*)-**5** over 40 h. The top spectrum is that of the initial solution of the (*S*)-**5** extractant and Aliquat 336 for reference.

HPLC analysis of the isolated amino acid following the 40h run shows that L-Phe was successfully converted to D-Phe during the continuous EECR with (*S*)-**5.** The isolated product has an optical purity >98% e.r. (**Supplementary Figure 42**).

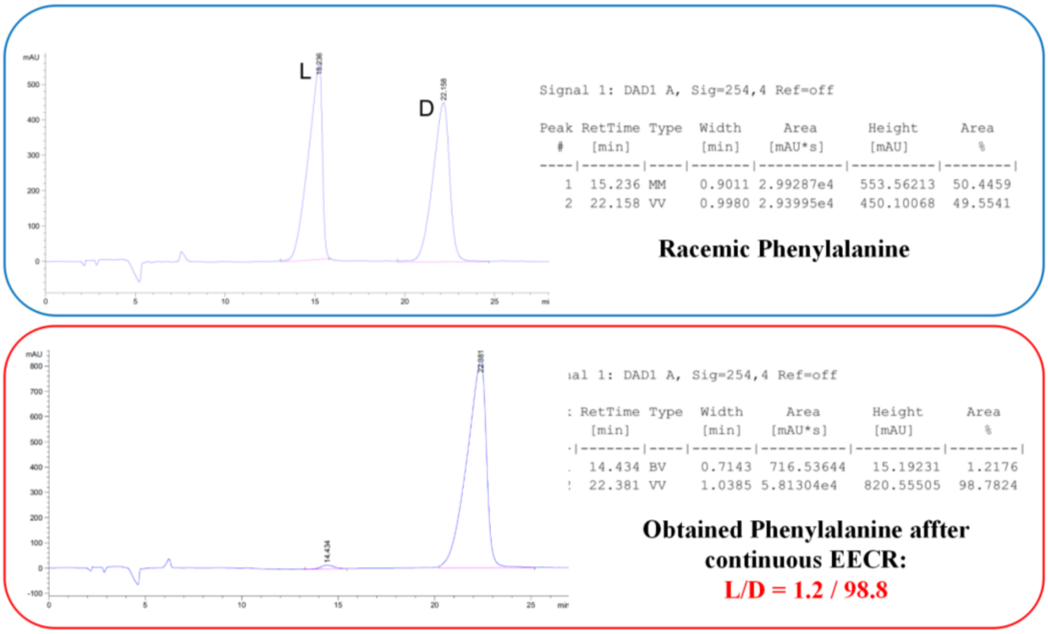

Supplementary Figure 42. HPLC chromatograms of racemic Phe (top, blue box) and of the Phe obtained from the continuous EECR of L-Phe with (*S*)-**5** (bottom, red box).

8. Model calculations

8.1 Procedure

The level of computation to obtain the final geometries and energies for all conformers in this study is the density functional theory supplemented with the Grimme's dispersion (namely D3) correction [3]. The BLYP functional, which uses the Becke's exchange [4] and the Lee-Yang-Parr's correlation [5], and the Pople's 6-311G(d) basis set were employed for the final optimization. We will refer this method to as BLYP-D3/6-311G*. We chose this method since the BLYP-D3 gives a minimal mean absolute deviation when tested for the S22 benchmark dataset of DNA base pairs, amino acid pairs and small model complexes [6].

The input geometries for the final optimization with the BLYP-D3/6-311G* were prepared as follows. In the first step, the crystallographic geometry of (*R*)-**5**-L-Phe anion, which is shown in Fig. 3 of the main article, was modified by replacing each one of the *tert*-butyl CH_3's with one H to provide each seed geometry for (*R*)-**4**-L-Phe. In similar ways, the seed geometries for (*R*)-**3**-L-Phe, (*R*)-**2**-L-Phe, and (*R*)-**1**-L-Phe were prepared. The seed geometries for (*R*)-**5**-L-Val and (*R*)-**5**-L-Ala were prepared similarly but these times the phenyl group in the amino acids were replaced by CH_3 and H, respectively. The seed geometries for the D-amino acid counterpart were prepared by exchanging α-H and the side group of the corresponding amino acid moiety. The seed geometries for the (*R*)-n, (n=**1**-**5**) were prepared after removing amino acid atoms from the geometries of the corresponding imine anion and adding one carbonyl oxygen.

In the second step, each seed geometry mentioned above was relaxed under molecular dynamics (MD) run for 10 ps at the density functional tight binding (DFTB) level of theory [4]. During the MD run the bath temperature was varied between 0.1 K and 290 K. This temperature range was needed to cover a wide range of geometry variation including the cis ↔ trans conversion in the uryl moiety. Occasionally, the (*R*) ↔ (*S*) interconversion was induced. However, as (*S*)-L-amino acid is a mirror image of (*R*)-D-amino amino acid, the result was shared by the two

mirror images. Typically, one seed geometry was run for 30 cycles of 10 ps MD with updated initial geometries. Then, several low energy geometries from 30 local geometries were selected for further optimization in the third step.

The third step was the pre-optimization with the BLYP-D3/6-31G level of theory. About 15 to 50 geometries per each imine anion or (*R*)-n were tried for the pre-optimization and those geometries whose energy was within about 10 kcal/mol to the global minimum value underwent the final optimization.

8.2 Geometries and energies for (*R*)-n

The geometries for the (*R*)-n (n=**1**-**5**) conformers are shown in Supplementary Figures 43-through 47 The relative values of the electronic part of the energy (E_e) and its zero-point energy (ZPE) correction (E_0 = E_e +ZPE) in kcal/mol are shown in parenthesis and square brackets, respectively. These relative values will be denoted with δE_e and δE_0, respectively. Only those conformers whose δE_e or δE_0 is less than 2 kcal/mol are shown and labelled with the alphabetic order.

The geometries in Supplementary Figures 43~47 may be classified into two categories. In the first category, one uryl hydrogen is oriented syn and the other uryl hydrogen is oriented anti to the uryl C=O. The anti uryl hydrogen makes a single hydrogen bond to either the ketyl oxygen or alcoholic oxygen. Then, the alcoholic hydrogen makes a hydrogen bond to the ketyl oxygen. This kind of structure is stabilized through a well-known resonance assisted hydrogen bond (RAHB). In the second category, the two uryl hydrogens are oriented anti to the uryl C=O and makes a double hydrogen bond to the ketyl oxygen. Then, the alcoholic hydrogen is pointing away from the uryl C=O. Thus, the geometries of the second category show a broken RAHB effect but they experience stabilization due to the double hydrogen bond. In which category a given (*R*)-n takes the geometry should be determined by other steric interactions as well as the hydrogen bonds. However, it is worth to mention that the anti-anti orientation of the uryl hydrogen (as in the geometries in the second

category) may undergo smaller structural change to bind an amino acid through the imine formation.

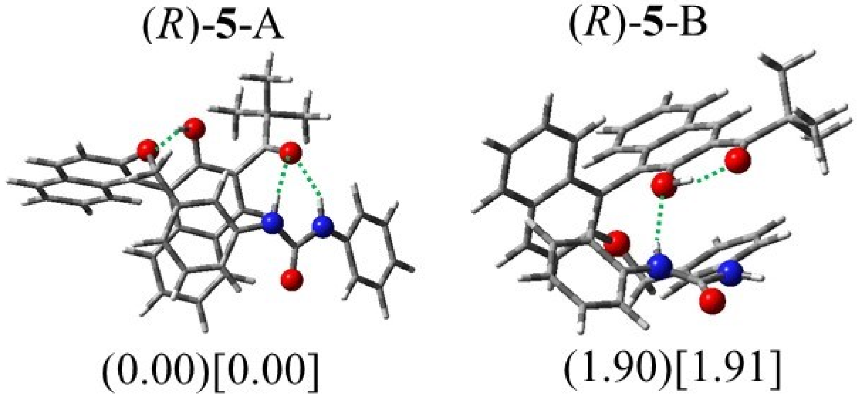

Supplementary Figure 43. Geometries of the two lowest energy conformers of (*R*)-**5** optimized at the BLYP-D3/6-311G* level of theory.

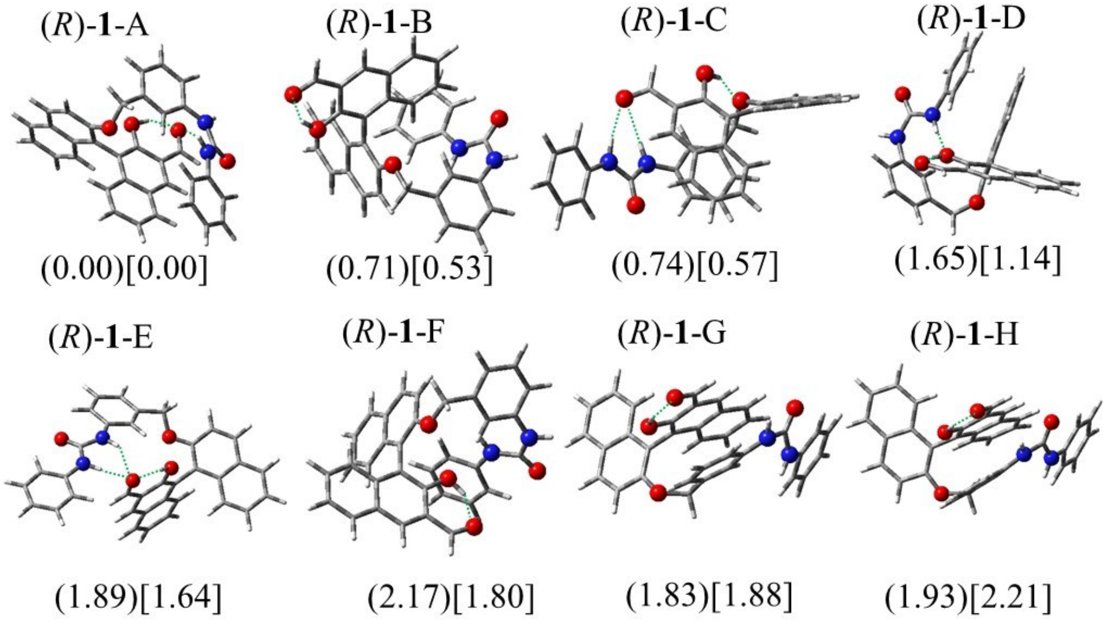

Supplementary Figure 44. Geometries of the eight lowest energy conformers of (*R*)-**1** (aldehyde) optimized at the BLYP-D3/6-311G* level of theory.

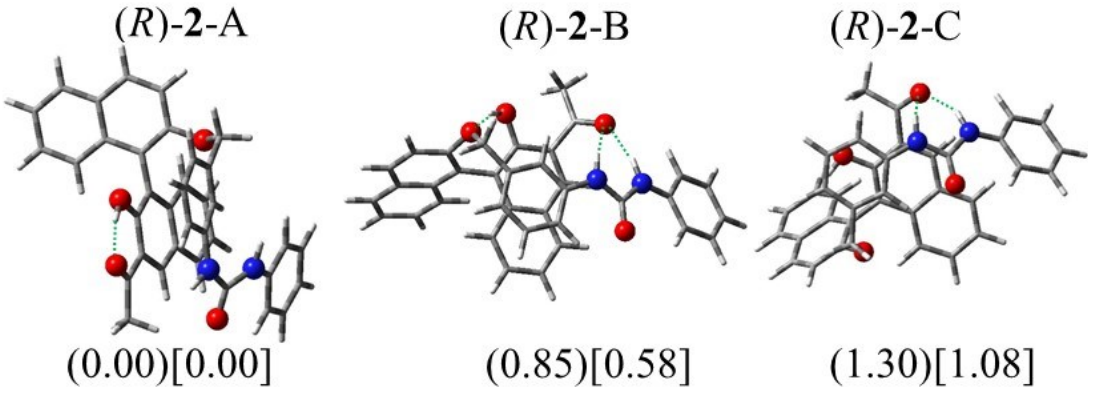

Supplementary Figure 45. Geometries of the three lowest energy conformers of (*R*)-**2** (methyl ketone) optimized at the BLYP-D3/6-311G* level of theory. The fourth lowest conformer has 2.61 and 2.69 kcal/mol for δE_e and δE_0 and is not shown.

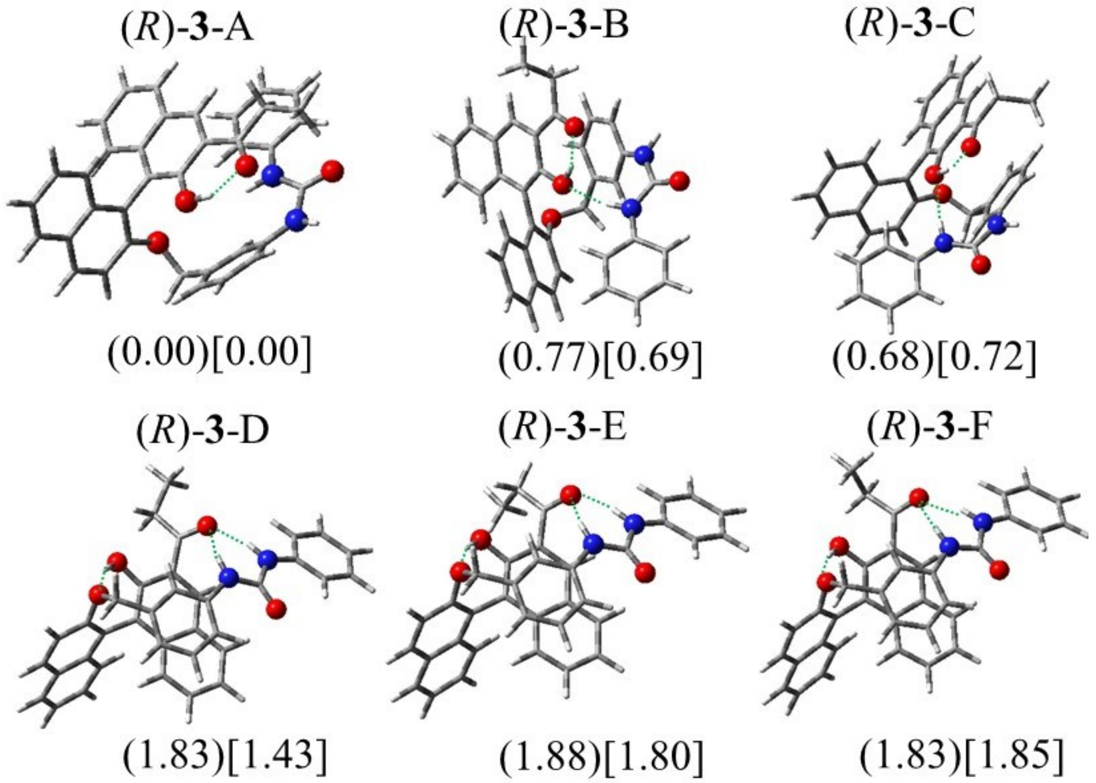

Supplementary Figure 46. Geometries of the six lowest energy conformers of (*R*)-**3** (ethyl ketone) optimized at the BLYP-D3/6-311G* level of theory.

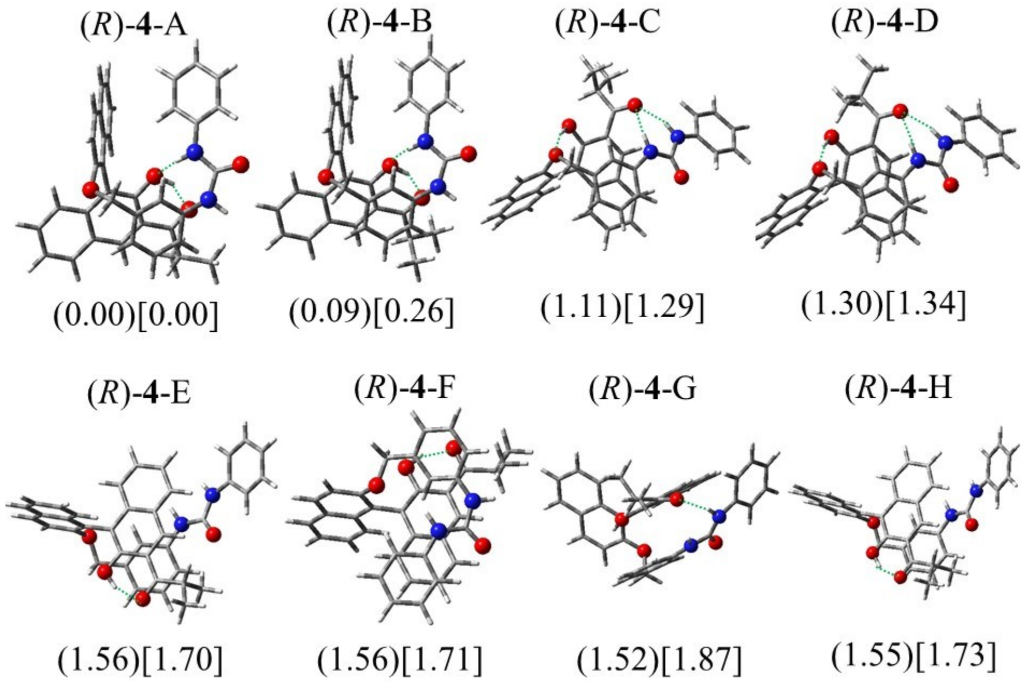

Supplementary Figure 47. Geometries of the eight lowest energy conformers of (*R*)-**4** (isopropyl ketone) optimized at the BLYP-D3/6-311G* level of theory.

8.3 Geometries and energies for imines of (*R*)-5-amino acids

From Supplementary Figures 48 to through 50, the geometries for the (*R*)-**5**-amino acid anion conformers are shown. The δE_e and δE_0 values in kcal/mol are shown in parenthesis and square bracket, respectively. Only those conformers whose δE_e or δE_0 was less than 2 kcal/mol are shown and labelled with the alphabetic order. Notice that in all the conformers the two uryl hydrogens are oriented anti-anti to the uryl C=O and make double hydrogen bonds with the two carboxylic acids of the amino acid moiety. Then, the alcoholic oxygen points to the oxygen of the carboxylic acid. We will classify the geometries of this kind as the category II. In the other kind of geometries (category I), the RABH feature is maintained. However, the geometries in category I have very high energies and are not shown in the figures.

In general, the energies of the (*R*)-**5**-L-amino acids are much lower than those of (*R*)-**5**-D-amino acids. This reflects the steric effect due to the bulky side group of the amino acid. This effect is reduced as the size of the side group is reduced. In other words, the energy difference between the imine anion with L- and D-amino acids is reduced in the order Phe > Val > Ala.

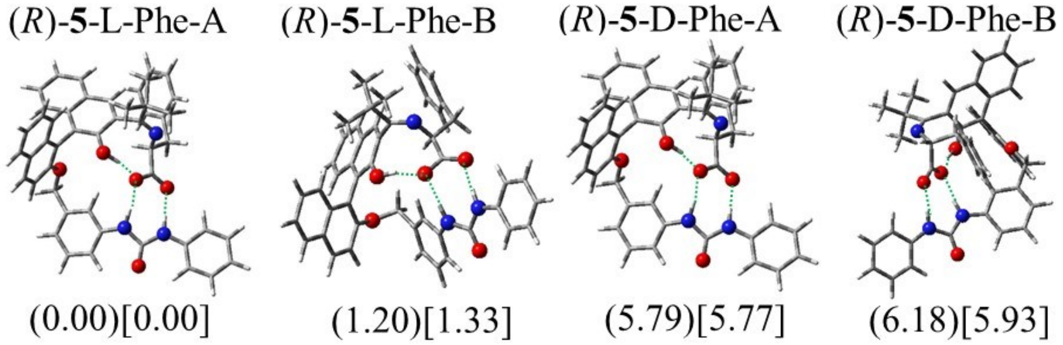

Supplementary Figure 48. Geometries of the two lowest energy conformers of (*R*)-**5**-L-Phe and the two lowest energy conformer of (*R*)-**5**-D-Phe optimized at the BLYP-D3/6-311G* level of theory. The third lowest conformer of (*R*)-**5**-L-Phe has δE_e and δE_0 values higher by 3.69 and 3.37 kcal/mol than those of (*R*)-**5**-L-Phe-A and is not shown. The third lowest conformer of (*R*)-**5**-D-Phe has δE_e and δE_0 values higher by 6.43 and 6.93 kcal/mol than those of (*R*)-**5**-L-Phe-A and is not shown.

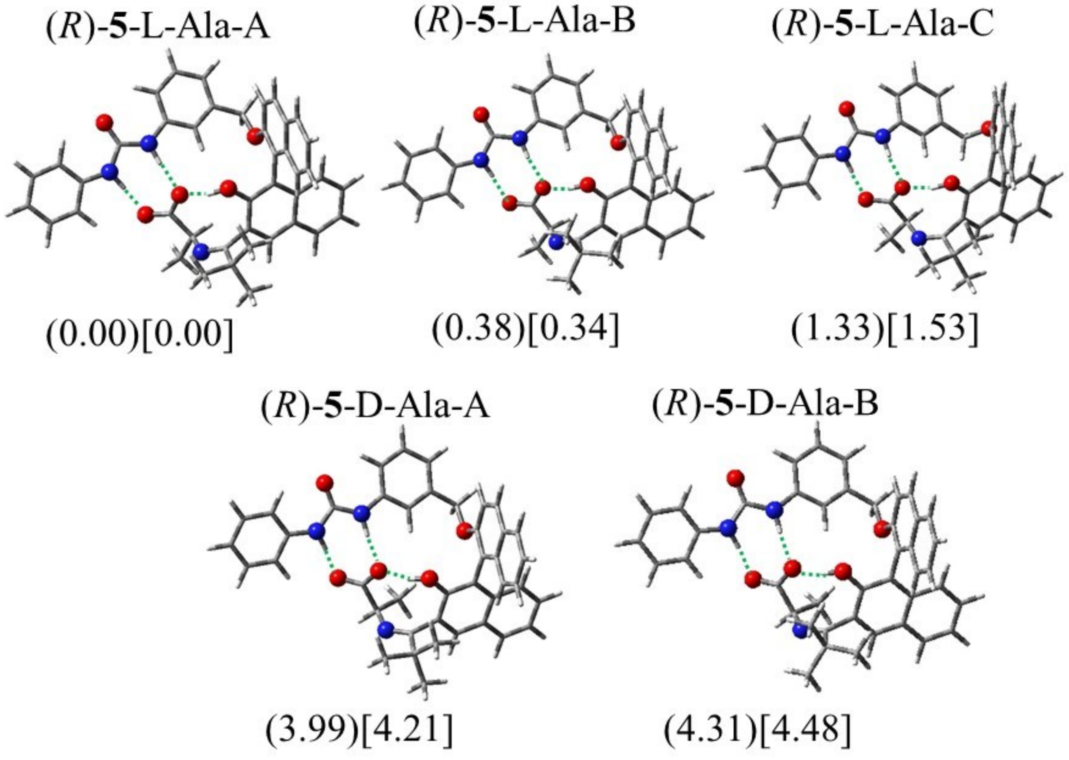

Supplementary Figure 49. Geometries of the three lowest energy conformers of (*R*)-**5**-L-Ala and the two lowest energy conformer of (*R*)-**5**-D-Ala.

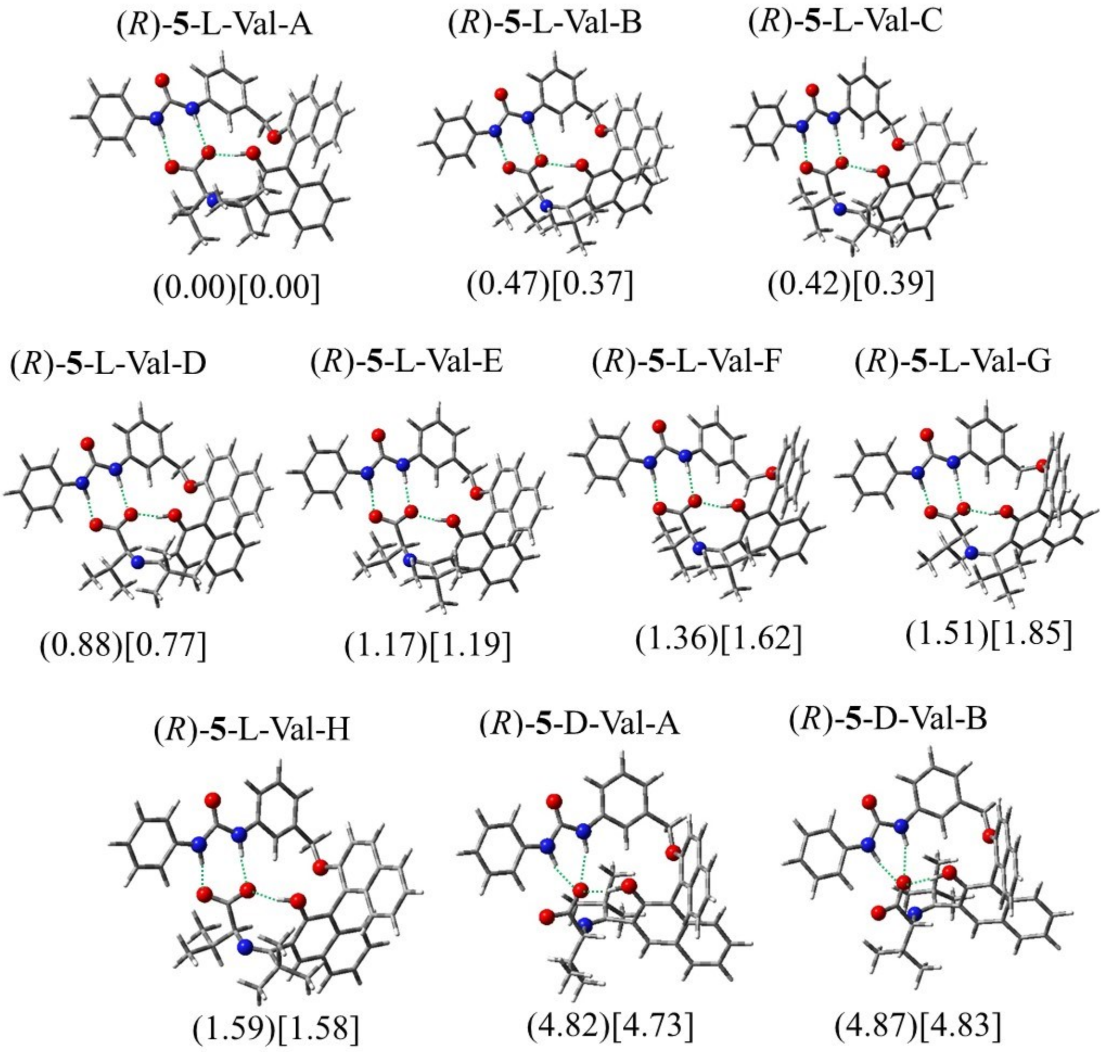

Supplementary Figure 50. Geometries of the eight lowest energy conformers of (*R*)-**5**-L-Val and the two lowest energy conformers of (*R*)-**5**-D-Val.

In Supplementary Table 4, the values for the enthalpy (H) and the free energy (G) at 298 K as well as E_e and E_0 for all the conformers shown in Supplementary Figures 48 through 50, obtained from the frequency analysis, are also listed. Here, the symbol δ implies the relative value and Δ represents the change over the imine formation reaction:

$$(R)\text{-n} + \text{amino acid anion} \rightarrow \text{imine anion} + H_2O$$

For example, $\Delta E_e = E_e\,(\text{imine anion}) + E_e\,(H_2O) - E_e\,((R)\text{-n}) - E_e\,(\text{amino acid anion})$.

In addition, the values of the two conformers labelled as X are included in Supplementary Table 4. The label X means that the corresponding conformer is in category I, while the other low energy ones are all in category II, and is not ranked

since the pre-optimized geometry is derived by replacing three H's in a stable geometry of (*R*)-**1**-Phe. The energies of the X conformers are very high because of the steric hindrance between the *tert*-butyl group of (*R*)-**5** and the side group of the amino acid moiety.

Supplementary Table 4. Energies (E_e and E_0) at 0 K, enthalpy (H) and free energy (G) at 298 K for (*R*)-**5**-amino acid conformers calculated at the BLYP-D3/6-311G* level of theory.[a]

Imine conformers		δE_e	δE_0	δG	$e^{-\delta E_0/RT}$	$e^{-\delta G/RT}$	ΔE_e	ΔE_0	ΔG	[b]
(*R*)-**5**-L-Phe	A	0.00	0.00	0.00	1.00	1.00	-31.49	-33.30	-28.63	II
	B	1.20	1.33	1.90	0.11	0.04	-30.29	-31.96	-26.73	II
	X	7.92	7.91	9.29	2×10^{-6}	2×10^{-7}	-23.57	-25.39	-26.67	I
(*R*)-**5**-D-Phe	A	5.79	5.77	6.32	6×10^{-5}	2×10^{-5}	-25.70	-27.52	-22.31	II
	B	6.18	5.93	5.13	4×10^{-5}	2×10^{-4}	-25.31	-27.37	-23.50	II
	X	19.0	19.0	19.1	9×10^{-14}	9×10^{-15}	-12.51	-14.31	-9.51	I
(*R*)-**5**-L-Val	A	0.00	0.00	0.00	1.00	1.00	-30.10	-32.49	-29.40	II
	B	0.47	0.37	0.03	0.53	0.96	-29.63	-32.12	-29.38	II
	C	0.42	0.39	0.44	0.52	0.48	-29.68	-32.11	-28.97	II
	D	0.88	0.77	0.65	0.27	0.34	-29.22	-31.72	-28.76	II
	E	1.17	1.19	1.08	0.13	0.16	-28.93	-31.30	-28.32	II
	F	1.36	1.62	2.27	0.06	0.02	-28.74	-30.87	-27.13	II
	G	1.51	1.85	2.42	0.04	0.02	-28.59	-30.64	-26.98	II
	H	1.59	1.58	1.66	0.07	0.06	-28.51	-30.91	-27.74	II
	X	10.5	11.3	13.2	6×10^{-9}	2×10^{-10}	-19.56	-21.24	-16.19	I
(*R*)-**5**-D-Val	A	4.82	4.73	5.12	3×10^{-4}	2×10^{-4}	-25.28	-27.76	-24.28	II
	B	4.87	4.83	5.14	3×10^{-4}	2×10^{-4}	-25.23	-27.66	-24.26	II
	X	16.3	16.8	18.1	5×10^{-13}	5×10^{-14}	-13.78	-15.68	-11.26	I
(*R*)-**5**-L-Ala	A	0.00	0.00	0.00	1.00	1.00	-31.59	-33.92	-31.03	II
	B	0.38	0.34	0.50	0.57	0.43	-31.22	-33.58	-30.53	II
	C	1.33	1.53	2.35	0.08	0.02	-30.26	-32.39	-28.63	II
	X	10.8	11.5	13.4	4×10^{-9}	2×10^{-10}	-20.80	-22.42	-17.66	I
(*R*)-**5**-D-Ala	A	3.99	4.21	4.31	8×10^{-4}	7×10^{-4}	-27.60	-29.71	-26.72	II
	B	4.32	4.48	4.79	5×10^{-4}	3×10^{-4}	-27.28	-29.44	-26.24	II
	X	14.8	15.1	16.1	8×10^{-12}	1×10^{-12}	-16.82	-18.83	-14.89	I

[a]: The relative values are denoted with δ, and the changes during the imine formation are denoted with Δ. [b]: This column shows the category of the conformer geometry. All the low energy conformers are in category II, as the lowest energy conformers in category I, labelled as X, are very high in energies.

Geometries and energies for (*R*)-n-Phe (n=1-4)

From Supplementary Figures 51 through 54, the geometries for the (*R*)-n-Phe (n=**1**-**4**) anion conformers are shown. Notice that in all the conformers the two uryl hydrogens are oriented anti-anti to the uryl C=O and make double hydrogen bonds with the two carboxylic acids of the amino acid moiety. The details of the double hydrogen bonding structure are classified into two, depending on whether the RABH structure is maintained (category I) or not (category II). In category II, the alcoholic hydrogen points toward the carboxyl oxygen to makes an extra hydrogen bond. The trend is that the category I geometries are dominated for (*R*)-**1**-L-Phe, the two category geometries are similar in energies for (*R*)-**2**-L-Phe and (*R*)-**3**-L-Phe, the category II geometries are more important for (*R*)-**4**-L-Phe and (*R*)-**5**-L-Phe. In contrast, the category II geometries are dominant for all (*R*)-n-L-Phe (n=**1**-**5**). As the result, the energy difference between the imine anions with L-Phe and D-Phe is small for n=**1**-**4** but is large for n=**5**.

In Supplementary Table 5, the relative values for the enthalpy (H) and the free energy (G) at 298 K as well as E_e and E_0 for all the conformers shown in Supplementary 51 through 54 and 48 are listed. Here, the symbol δ implies the relative value and Δ represents the change over the imine formation reaction. Especially interesting are the Δ values since they may be related to the product yield. The Δ values for n=**5** and n=**1** are much lower than those for others. Here we also list the values for the hypothetical unranked conformers X and Y. They are the category I and II conformers, respectively, optimized from nonstandard routes in which preoptimization geometries were obtained by replacing hydrogens of (*R*)-1-Phe with methyl groups successively.

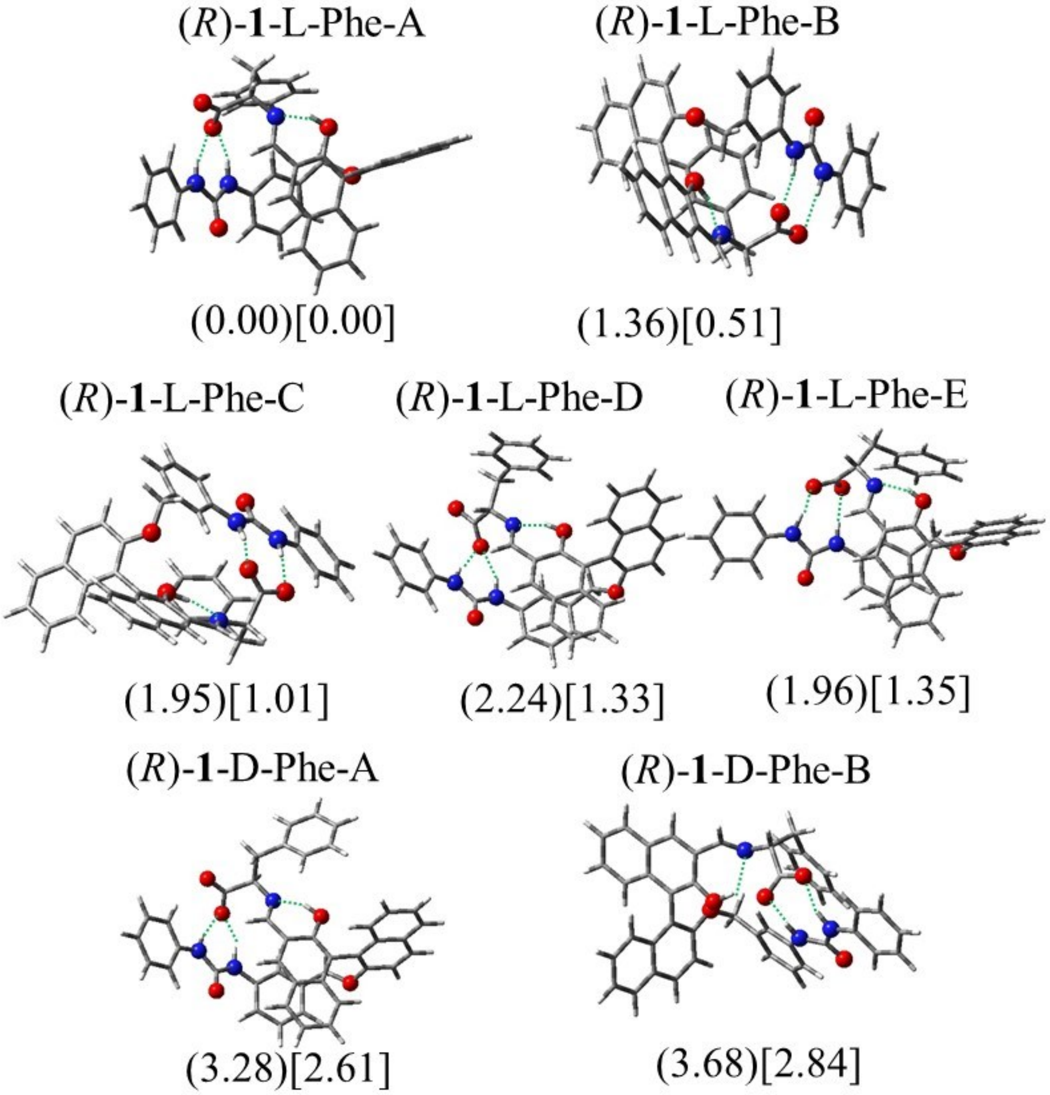

Supplementary Figure 51. Geometries of the five lowest energy conformers of (*R*)-**1**-L-Phe and the two lowest energy conformers of (*R*)-**1**-D-Phe.

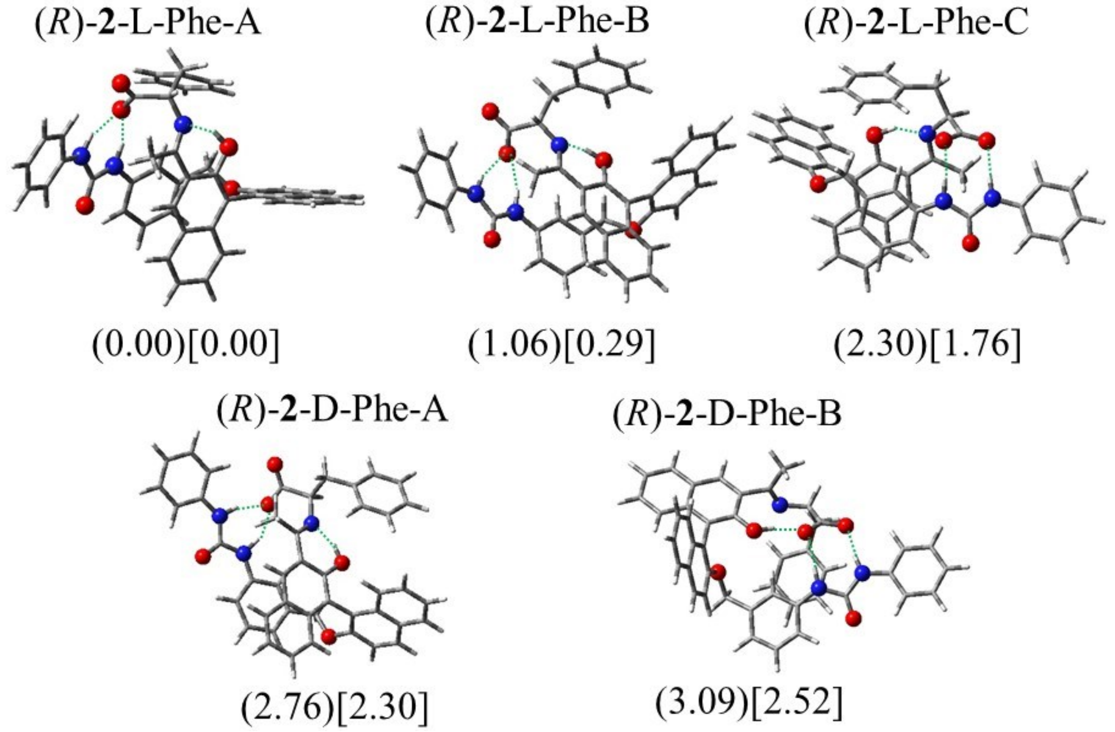

Supplementary Figure 52. Geometries of the three lowest energy conformers of (*R*)-**2**-L-Phe and the two lowest energy conformer of (*R*)-**2**-D-Phe.

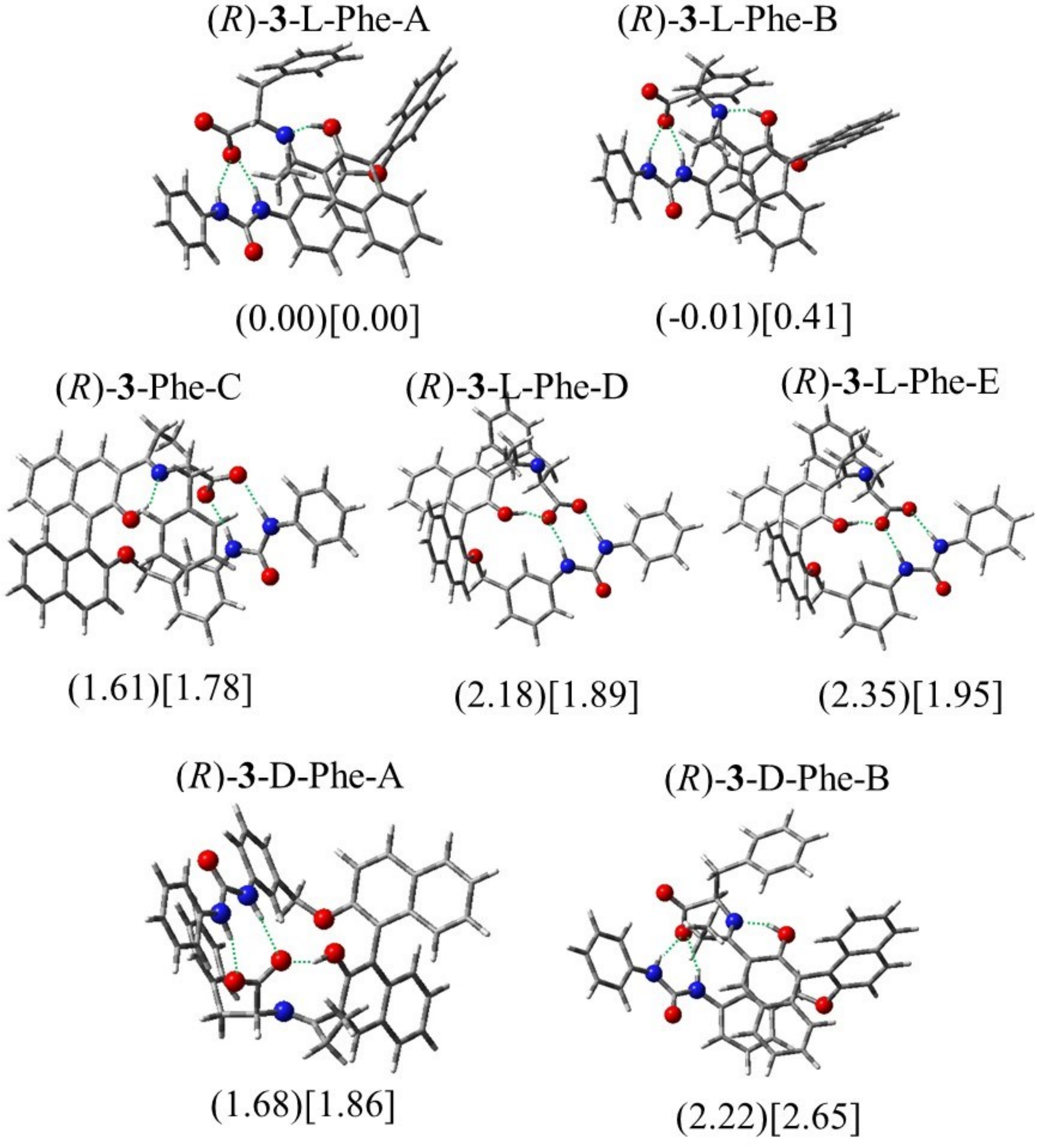

Supplementary Figure 53. Geometries of the five lowest energy conformers of (*R*)-**3**-L-Phe and the two lowest energy conformers of (*R*)-**3**-D-Phe.

`

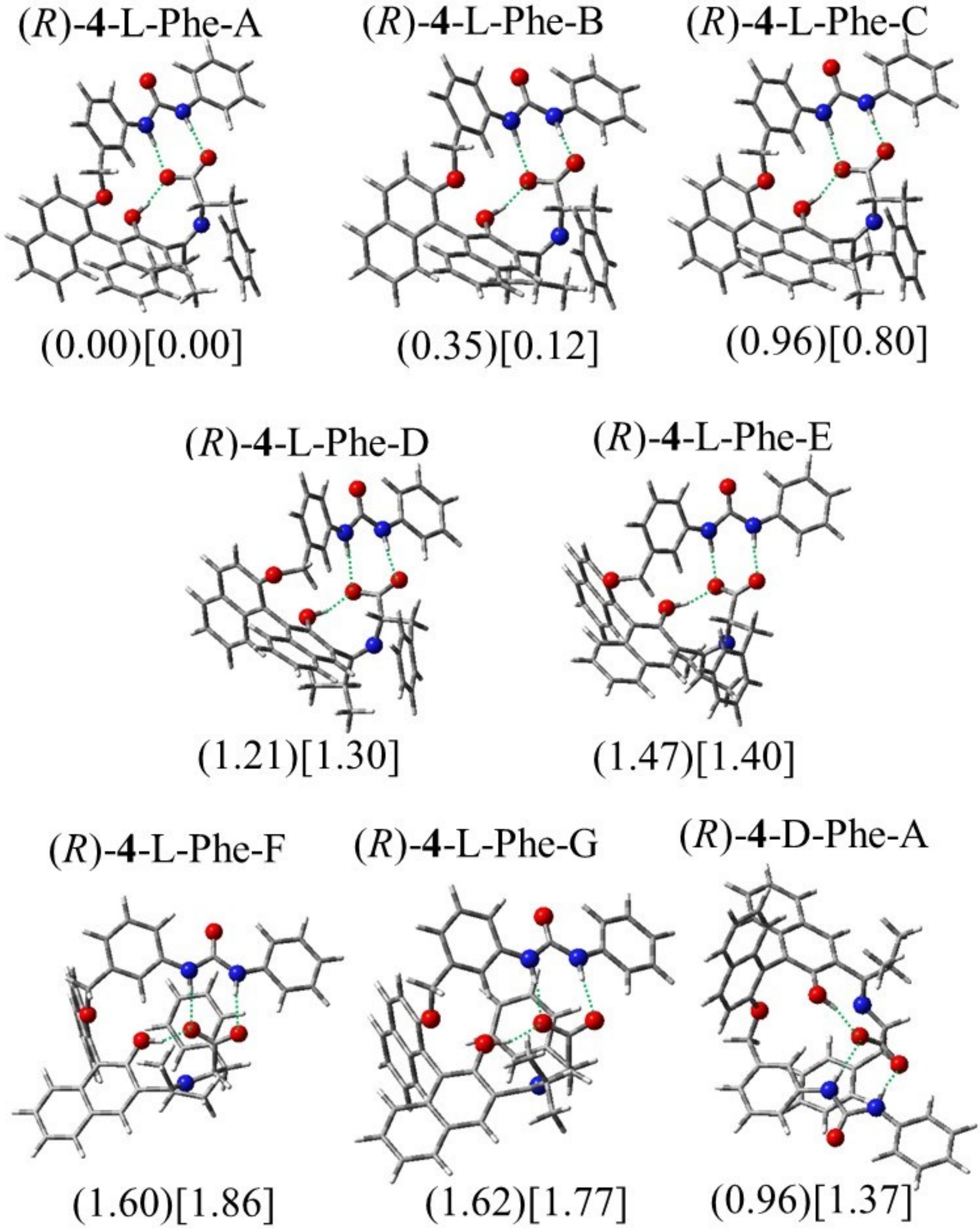

Supplementary Figure 54. Geometries of the seven lowest energy conformers of (*R*)-**4**-L-Phe and the lowest energy conformer of (*R*)-**4**-D-Phe.

Supplementary Table 5. Energies (E_e and E_0) at 0 K, enthalpy (H) and free energy (G) at 298 K for (*R*)-n-amino acid (n = **1**-**5**) conformers calculated at the BLYP-D3/6-311G* level of theory.[a]

Imine conformers[c]		δE_e	δE_0	δG	$e^{-\delta E_0/RT}$	$e^{-\delta G/RT}$	ΔE_e	ΔE_0	ΔG	[b]
(*R*)-**5**-L-Phe	A	0.00	0.00	0.00	1.00	1.00	-31.49	-33.30	-28.63	II
	B	1.20	1.33	1.90	0.11	0.04	-30.29	-31.96	-26.73	II
	X	7.92	7.91	9.30	2×10^{-6}	2×10^{-7}	-23.57	-25.39	-19.33	I
(*R*)-**5**-D-Phe	A	5.79	5.77	6.32	6×10^{-5}	2×10^{-5}	-25.70	-27.52	-22.31	II
	B	6.18	5.93	5.13	4×10^{-5}	2×10^{-4}	-25.31	-27.37	-23.50	II
	X	19.0	19.0	19.1	9×10^{-14}	9×10^{-15}	-12.51	-14.31	-9.51	I
(*R*)-**1**-L-Phe	A	0.00	0.00	0.00	1.00	1.00	-35.33	-36.30	-30.01	I
	B	1.36	0.51	-0.29	0.42	1.64	-33.97	-35.79	-30.31	I
	C	1.95	1.01	-0.02	0.18	1.04	-33.38	-35.28	-30.04	I
	D	2.24	1.33	-0.31	0.10	1.68	-33.09	-34.96	-30.32	I
	E	1.96	1.35	0.37	0.10	0.53	-33.38	-34.94	-29.64	I
	Y	6.29	5.62	5.01	8×10^{-5}	2×10^{-4}	-29.05	-30.68	-25.01	II
(*R*)-**1**-D-Phe	A	3.28	2.61	0.74	0.01	0.29	-32.05	-33.68	-29.28	I
	B	3.68	2.84	2.33	8×10^{-3}	0.02	-31.66	-33.45	-27.69	I
	Y	10.5	9.69	7.89	8×10^{-8}	2×10^{-6}	-24.80	-26.60	-22.12	II
(*R*)-**2**-L-Phe	A	0.00	0.00	0.00	1.00	1.00	-31.33	-32.77	-26.83	I
	B	1.06	0.29	-1.17	0.62	7.18	-30.27	-32.48	-27.99	I
	C	2.30	1.76	0.85	0.05	0.87	-29.03	-31.01	-25.98	I
	Y	3.09	2.26	0.38	0.02	0.05	-28.24	-30.51	-26.45	II
(*R*)-**2**-D-Phe	A	2.76	2.30	1.28	0.02	0.12	-28.57	-30.47	-25.55	I
	B	3.08	2.52	1.00	0.01	0.18	-28.24	-30.25	-25.83	II
(*R*)-**3**-L-Phe	A	0.00	0.00	0.00	1.00	1.00	-30.04	-31.83	-27.10	I
	B	-0.01	0.41	1.33	0.50	0.11	-30.05	-31.42	-25.77	I
	C	1.61	1.79	1.58	0.05	0.07	-28.43	-30.04	-25.53	I
	D	2.18	1.88	1.04	0.04	0.17	-27.86	-29.94	-26.07	II
	E	2.35	1.95	0.97	0.04	0.20	-27.69	-29.88	-26.14	II
(*R*)-**3**-D-Phe	A	1.68	1.86	2.12	0.04	0.03	-28.36	-29.97	-24.99	II
	B	2.22	2.65	3.76	0.01	2×10^{-3}	-27.82	-29.18	-23.34	I
(*R*)-**4**-L-Phe	A	0.00	0.00	0.00	1.00	1.00	-29.23	-30.93	-25.98	II
	B	0.35	0.12	-0.37	0.81	1.88	-28.88	-30.81	-26.35	II
	C	0.96	0.80	0.17	0.26	0.75	-28.27	-30.14	-25.81	II
	D	1.21	1.30	1.77	0.11	0.05	-28.02	-29.63	-24.21	II
	E	1.47	1.40	1.41	0.09	0.09	-27.76	-29.53	-24.57	II
	F	1.60	1.86	3.13	0.04	0.01	-27.63	-29.07	-22.85	II
	G	1.62	1.77	2.75	0.05	0.01	-27.61	-29.16	-23.23	II
	X	3.71	3.78	4.41	2×10^{-3}	6×10^{-4}	-25.51	-27.15	-21.56	I
(*R*)-**4**-D-Phe	A	0.96	1.37	2.06	0.10	0.03	-28.27	-29.56	-23.92	I
	Y	5.89	6.08	7.04	3×10^{-5}	7×10^{-6}	-23.34	-24.85	-18.94	II

[a]: The relative values are denoted with δ. The values of change during the imine formation are denoted with Δ. [b]: This column shows the category of the conformer geometry. [c]: Conformers are labelled in alphabetic order in the order of increasing δE_0, except conformers X and Y.

Supplementary References

[1] Park, H.; Kim, K. M.; Lee, A.; Ham, S.; Nam, W.; Chin, J. *J. Am. Chem. Soc.* **2007**, *129*, 1518.

[2] Huang, H.; Nandhakumar, R.; Choi, M.; Su, Z.; Kim, K. M. *J. Am. Chem. Soc.* **2013**, *135*, 2653.

[3] Grimme, S.; Antony, J.; Ehrlich, S.; Krieg, H. A *J. Chem. Phys.* **2010***, 132*, 154104.

[4] Becke, A. D. *Phys. Rev. A.* **1988**, *38*, 3098.

[5] Lee, C.; Yang, W.; Parr, R. G. *Phys. Rev. B.* **1988**, *37*, 785.

[6] Elstner, M.; Porezag, D.; Jungnickel, G.; Elsner, J.; Haugk, M.; Frauenheim, T.; Suhai, S.; Seifert, G. *Phys. Rev. B* **1998**, *58*, 7260-7268.
